# Effect of Prophylactic Tropisetron on Post-Operative Nausea and Vomiting in Patients Undergoing General Anesthesia: Systematic Review and Meta-Analysis with Trial Sequential Analysis

**DOI:** 10.3390/jpm14080797

**Published:** 2024-07-27

**Authors:** In Jung Kim, Geun Joo Choi, Hyeon Joung Hwang, Hyun Kang

**Affiliations:** 1 Department of Anesthesiology and Pain Medicine, Chung-Ang University Hospital, 102 Heukseok-ro, Dongjak-gu, Seoul 06973, Republic of Korea; injung91@cau.ac.kr (I.J.K.); pistis23@cau.ac.kr (G.J.C.); 2 Department of Anesthesiology and Pain Medicine, Chung-Ang University College of Medicine, 84 Heukseok-ro, Dongjak-gu, Seoul 06911, Republic of Korea; hhj515@cau.ac.kr

**Keywords:** anesthesia, general, tropisetron, systematic review, randomized controlled trial, PONV

## Abstract

This systematic review and meta-analysis of randomized controlled trials (RCTs) with trial sequential analysis (TSA) aimed to comprehensively evaluate and compare the efficacy of the prophylactic administration of tropisetron in the prevention of the incidence of post-operative nausea and vomiting (PONV) in patients undergoing surgery under general anesthesia. This study was registered with PROSPERO (CRD42024372692). RCTs comparing the efficacy of the perioperative administration of tropisetron with that of a placebo, other anti-emetic agents, or a combination of anti-emetic injections were retrieved from the databases of Ovid-MEDLINE, Ovid-EMBASE, the Cochrane Central Register of Controlled Trials, and Google Scholar. The frequency of rescue anti-emetic use (RA) and the incidence of PON, POV, and PONV (relative risk [RR]: 0.718; 95% confidence interval [CI] 0.652–0.790; I^2^ = 0.0, RR: 0.587; 95% CI 0.455–0.757; I^2^ = 63.32, RR: 0.655; 95% CI 0.532–0.806; I^2^ = 49.09, and RR: 0.622; 95% CI 0.552–0.700; I^2^ = 0.00, respectively) in the tropisetron group were lower than those in the control group; however, the incidence of complete response (CR) was higher in the tropisetron group (RR: 1.517;95% CI 1.222–1.885; I^2^ = 44.14). TSA showed the cumulative Z-curve exceeded both the conventional test and trial sequential monitoring boundaries for RA, PON, POV, and PONV between the tropisetron group and the control group. Thus, the prophylactic administration of tropisetron exhibited superior efficacy in the prevention of PON, POV, and PONV. Furthermore, a lower incidence of RA and a higher incidence of CR were observed with its use.

## 1. Introduction

Post-operative nausea and vomiting (PONV) are frequent and uncomfortable side effects of surgery that affect patient well-being, recovery processes, and healthcare expenses [1]. PONV continues to affect 20–30% of the general surgical population and up to 80% of high-risk individuals despite the advances in the field of anesthesia and perioperative care [2,3].

Selective serotonin 5-HT_3_ receptor antagonists are the cornerstone of pharmacological interventions for the management of PONV [4,5]. Tropisetron, a selective serotonin 5-HT_3_ antagonist with minimal affinity for 5-HT_4_ receptors, is a promising anti-emetic agent owing to its efficacy and favorable safety profile [6,7]. Tropisetron exhibits a prolonged duration of action, with an elimination half-life of 7 to 8 h and 30 to over 40 h in rapid and slow metabolizers, respectively. This prolonged duration of action distinguishes it from other agents in its class and renders it suitable for use in single-dose PONV prophylaxis [8,9]. Furthermore, tropisetron exhibits partial agonistic activity at the α_7_-nicotinic receptors, suggesting potential modulatory effects other than serotonin receptor blockade [10,11,12].

Various clinical guidelines for the prevention and management of PONV, such as the First Consensus Guidelines for the Management of PONV [13] and the subsequent revisions (including the most recent fourth consensus guidelines issued in 2020), recommend the administration of tropisetron [1]. Although evidence has been accumulated to support the inclusion of tropisetron, systematic reviews and meta-analyses exploring its efficacy have reported conflicting and variable results [14,15,16]. Furthermore, the absence of updated references within these guidelines, particularly in those of systematic reviews and meta-analyses, underscores the necessity for a thorough re-evaluation of the available data to ascertain the efficacy of tropisetron.

This meta-analysis aimed to systematically examine and evaluate randomized controlled trials (RCTs) that evaluated the prophylactic efficacy of tropisetron in the prevention of PONV in patients who underwent surgery under general anesthesia. Trial sequential analysis (TSA) was used in this study to enhance statistical robustness.

## 2. Materials and Methods

### 2.1. Protocol and Registration

The methodological framework of this systematic review and meta-analysis with TSA was designed following recommendations from the Preferred Reporting Items for Systematic Review and Meta-Analysis Protocols (PRISMA-P). The study protocol was registered in the PROSPERO network (registration number: CRD42024372692; www.crd.york.ac.uk/Prospero (accessed on 26 April 2024)). This study was performed following the protocol recommended by the Cochrane Collaboration [17,18] and adhering to the PRISMA statement guidelines [19].

### 2.2. Inclusion and Exclusion Criteria

The inclusion and exclusion criteria were determined before commencing the study. RCTs that compared the efficacy profiles of tropisetron with those of controls, other anti-emetic agents, or a combination of anti-emetic agents were eligible for inclusion. The PICO criteria of this study were as follows:

1. Patients (P): Adult patients undergoing surgery under general anesthesia.
2. Intervention (I): Tropisetron injection to prevent the incidence of PONV.
3. Comparison (C): A placebo or other anti-emetic agents, such as HT3 receptor blockers, NK receptor blockers, antihistamines, anticholinergics, and steroids, or a combination of anti-emetic injections applied equally to both groups.
4. Outcome measurements (O): The incidence of post-operative nausea (PON), post-operative vomiting (POV), and PONV; use of rescue anti-emetic agents (RA); and complete response (CR).
5. Outcomes recorded during all post-operative phases were included [20]. The data from the first time point were selected as the outcome of interest if data were collected at multiple time points. For instance, the data collected at 0 h were selected if the study reported data collected at 0, 2, 4, 6, and 24 h post-operatively. Data from studies that did not mention a specific time point were included to maximize the number of studies.
6. Study design (SD): RCTs. Studies that satisfied the following criteria were excluded: (1) studies that included pediatric patients; (2) studies that included patients who did not receive general anesthesia (for instance, studies that included patients who received spinal or epidural anesthesia); (3) studies that investigated the treatment effect of tropisetron; and (4) case reports, case series, editorials or letters to the editor, reviews, and animal or laboratory studies.

### 2.3. Systematic Search

Two researchers (KH and HHJ) performed an independent systematic literature search in April 2024. The databases of Ovid-MEDLINE, Ovid-EMBASE, the Cochrane Central Register of Controlled Trials (CENTRAL), and Google Scholar were searched to retrieve relevant articles. The Supplementary Materials provides a detailed overview of the search strategy, which incorporates free text, Medical Subject Headings (MESH), and EMTREE terms. In addition, clinical trial registries were searched to identify completed RCTs that have not been published yet. Open SIGLE was used to retrieve gray literature. The reference lists were imported into Endnote software 9.3 (Thompson Reuters, Los Angeles, CA, USA), and duplicate articles were excluded. The reference lists of the selected original articles were thoroughly reviewed to retrieve additional articles until no further relevant references could be identified. Language- or publication date-related constraints were not applied. The Supplementary File lists the search terms.

### 2.4. Study Selection

Two investigators (KIJ and CGJ) independently reviewed the titles and abstracts identified using the search strategy. The full text of the article was obtained and screened if the title or abstract was deemed suitable for inclusion. The full text of relevant articles selected by at least one author was procured and assessed. Papers published by the same authors, organizations, or countries were compared to minimize data duplication. Two investigators independently evaluated the articles that met the inclusion criteria. Any disagreements between the investigators were resolved by reaching a consensus via discussion. A third investigator (KH) was consulted if a consensus could not be reached.

### 2.5. Data Extraction

Two researchers (KIJ and HHJ) independently retrieved all inter-related data from the included studies using a pre-defined, standardized data collection form and cross-checked the findings. Any disagreements between the investigators were resolved by reaching a consensus via discussion. A third investigator (KH) was consulted if a consensus could not be reached. The spreadsheet for data extraction included the following items: (1) title; (2) name of the first author; (3) name of the journal; (4) year of publication; (5) study design; (6) clinical trial registration number; (7) country; (8) language; (9) risk of bias; (10) conflict of interests; (11) number of patients included; (12) sex of the patients; (13) age of the patients; (14) height of the patients; (15) weight of the patients; (16) duration of anesthesia or surgery; (17) physical status according to the American Society of Anesthesiologists (ASA) classification; (18) inclusion criteria; (19) exclusion criteria; (20) type of anesthesia; (21) type of surgery; (22) the agent used for anesthetic induction; (23) the agent used for the maintenance of anesthesia; (24) the type and dose of experimental drug (tropisetron or other anti-emetic agents or combination of anti-emetic agents); (28) rescue analgesic agents; (29) definitions of nausea, vomiting, and retching; (30) number of cases with PON, POV, and PONV; (31) the frequency of administering RA; and (32) CR.

The data were first extracted from tables or text. The corresponding authors of studies with incomplete or missing data were contacted to obtain the necessary information. Plot Digitizer (version 2.6.8; http://plotdigitizer.sourceforge.net (accessed on 29 March 2024)), an open-source software program, was used to extract the missing data from the available data if attempts to contact the corresponding author were unsuccessful.

### 2.6. Risk of Bias Assessment

Two independent investigators (CGJ and HHJ) critically appraised the quality of each study using the revised Cochrane risk of bias tool for randomized trials (RoB 2.0 version) [21]. Each domain of the included studies was evaluated and rated as follows: D1, bias resulting from the randomization process; D2, bias resulting from deviations from the intended interventions; D3, bias resulting from missing outcome data; D4, bias resulting from the measurement of the outcome; and D5, bias resulting from the selection of reported results. The overall risk of bias was classified as follows: (1) low risk, the risk of bias for all domains is low; (2) high risk, the risk of bias for at least one domain is high or the risk of bias for multiple domains is of some concern; and (3) some concern, the overall judgment is neither low nor high. Any disagreements between the investigators were resolved by reaching a consensus via discussion. A third investigator (KH) was consulted if a consensus could not be reached.

### 2.7. Statistical Analysis

#### 2.7.1. Conventional Meta-Analysis

Comprehensive Meta-Analysis software (version 2.0; Englewood, NJ, USA, 2008) was used to conduct all meta-analyses. Two authors (KIJ and HHJ) independently entered all the data into the software and calculated the pooled risk ratios (RRs) and 95% confidence intervals (Cis) for each outcome. Cochran’s Q test, Higgins’ I^2^, τ using the DerSimonian–Laird estimator, and the prediction interval (PI) method were used to assess heterogeneity. The PI was not calculated if τ = 0.0 [22]. A significance level of 10% (*p* < 0.1) in Cochran’s Q test or an I^2^ value of >50% indicated considerable heterogeneity. A fixed effects model was selected if the significance level in Cochran’s Q test was >0.10 and the I^2^ value was <50%. A random effects model was selected if the I^2^ value was >50% [23]. A sensitivity analysis was performed in the case of heterogeneous outcomes by removing one study at a time to detect changes in the results. The mean and standard deviation were calculated if the data were reported as median (P_25_–P_75_), median (range), or mean (standard error of the mean) [17,24]. The number needed to treat (NNT) was calculated based on the absolute risk reduction to estimate the overall clinical impact of the intervention.

Begg’s funnel plot and Egger’s linear regression test were used to assess publication bias. The asymmetry of the funnel plot or a *p*-value of <0.1 in Egger’s test indicated the presence of publication bias. A trim-and-fill analysis was performed in such cases. The presence of publication bias was not assessed if <10 studies were included [25].

#### 2.7.2. Trial Sequential Analysis

TSA was performed to determine the required information size (RIS) and assess whether the results were conclusive [26]. The cumulative Z-curve was constructed using a fixed or DerSimonian and Laird approach (*DL*) random effects model. TSA was conducted such that the overall risk of type I errors was maintained at 5%.

A sufficient level of evidence to accept or reject the anticipated intervention effect was considered to have been reached if the cumulative Z-curve crossed the trial sequential monitoring boundary or entered the futility area; no further studies were required thereafter. The evidence to conclude was considered to be insufficient if the Z-curve did not cross any boundaries and RIS was not reached, indicating the requirement for further studies.

RIS was estimated based on the proportion of patients with an outcome in the control or other anti-emetic group (the cumulative proportion of patients with an event relative to all patients in the control or other anti-emetic group), a relative risk reduction of 20% in the tropisetron group, an alpha of 5% for all outcomes, a beta of 20%, and the observed diversity, as suggested by the trials in TSA.

### 2.8. Quality of Evidence

The quality of the evidence was evaluated using the Grading of Recommendations, Assessment, Development, and Evaluation (GRADE) system. The quality of the evidence was assessed sequentially, the risk–benefit balance was evaluated, and the strength of the recommendations was appraised subsequently [27]. The quality of the evidence was classified into the following categories: (1) high, the confidence in the effect estimate is unlikely to change with further research; (2) moderate, additional research is likely to result in significant changes in the confidence in the effect estimate and alter the estimate; (3) low, additional study is likely to result in significant changes in the confidence in the effect estimate and alter the estimate; and (4) very low, no effect estimate is certain.

## 3. Results

### 3.1. Literature Search and Study Selection

**Figure 1:**
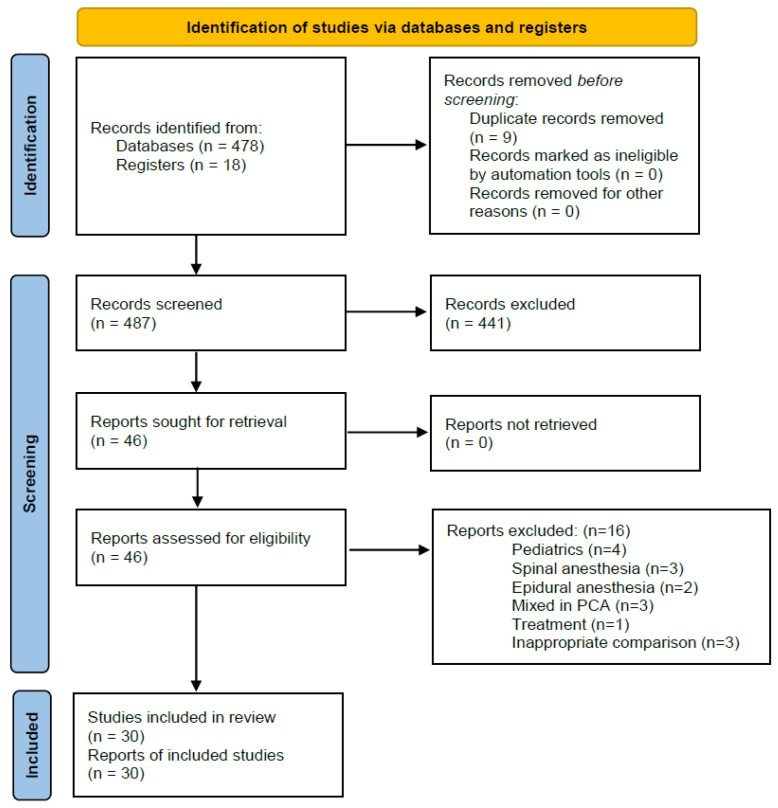
PRISMA flow diagram of the search for randomized controlled trials and the inclusion and exclusion criteria.

A total of 478 articles published until 15 April 2024 were retrieved by searching the databases of Ovid-MEDLINE, OVID-EMBASE, CENTRAL, and Google Scholar. A manual search yielded an additional 18 articles. Among the 487 articles retained after the exclusion of duplicate articles (n = 9), 441 articles that were judged to be unsuitable after reviewing the titles and abstracts were excluded. The kappa value between the two investigators for literature selection was 0.876 at this stage. A detailed review of the full texts of the 44 remaining articles resulted in the exclusion of 16 articles. The Supplementary File lists the reasons for the exclusion of these articles. The kappa value between the two investigators for article selection was 0.851 at this stage. Thirty studies involving a total of 5175 patients were included in the systematic review and meta-analysis with TSA (Figure 1).

### 3.2. Study Characteristics

Table 1 describes the characteristics of the 30 studies that satisfied the inclusion criteria.

### 3.3. Risk of Bias

Table 2 describes the findings of the risk of bias assessment performed using the Cochrane tool for the included studies. The studies were judged to have low risk [31,32,33,35,37,38,40,41,44,50,51,52,54,55], some concerns [28,30,34,36,39,42,43,45,46,47,48,49], or high risk [29,56,57].

Bias resulting from deviations from the intended intervention, missing outcome data, or the selection of the reported results was assessed as ‘low risk’ in all studies. Bias arising from the randomization process and measurement of the outcome data was assessed as ‘some concern’ in 13 [28,29,30,34,39,42,43,45,46,47,48,49] and four [29,36,56,57] studies, respectively.

### 3.4. Quantitative Meta-Analysis

#### 3.4.1. Post-Operative Nausea

**Figure 2:**
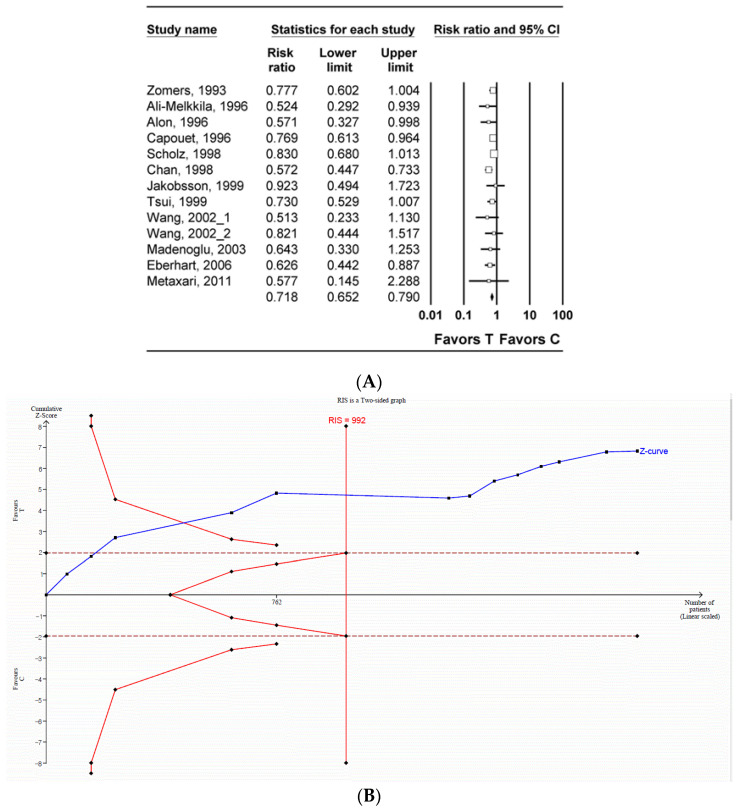
Incidence of post-operative nausea in the tropisetron and control groups. (**A**) Forest plot. The figure depicts individual trials as filled squares, with the relative sample size and 95% confidence interval (CI) of the difference represented as a solid line. The diamond shape represents the pooled estimate and uncertainty for the combined effect. The pooled estimate indicates that the incidence of post-operative nausea in the tropisetron group is lower than that in the control group [29,30,32,33,35,40,42,48,49,50,51,56,57]. (**B**) Trial sequential analysis plot. The uppermost and lowermost complete red curves represent the trial sequential monitoring boundary lines for benefit and harm, respectively. The horizontal dotted red line represents the conventional boundaries for statistical significance. The triangular red lines on the right side represent the futility boundaries. The blue solid line represents the cumulative z-curve. The number on the *x*-axis indicates the required information size (n = 992). The cumulative Z-curve exceeds the conventional and trial sequential monitoring boundaries, favoring the use of tropisetron over the use of the control for the prevention of post-operative nausea.

The incidence of PON was investigated in 13 studies (1955 patients) [29,30,32,33,35,40,42,48,49,50,51,56,57]. The incidence of PON in the tropisetron group was significantly lower than that in the control group (n = 12, relative risk [RR]: 0.718; 95% CI 0.652–0.790; I^2^ = 0.0; P_chi_^2^ = 0.612; τ = 0.0; number needed to treat benefit [NNTB]: 8; 95% CI NNTB 6 to NNTB 12) (Figure 2A, Table 3). The incidence of PON in the tropisetron group continued to be significantly lower than that in the control group even after the inclusion of studies [35,52,54] that compared dexamethasone to tropisetron combined with dexamethasone (n = 15, RR: 0.814; 95% CI 0.683–0.971; I^2^ = 65.38; P_chi_^2^ < 0.001; τ = 0.070; 95% PI 0.625–1.060; NNTB: 7; 95% CI NNTB 6 to NNTB 10) (Supplementary Figure S1, Table 3) [29,30,32,33,35,40,42,48,49,50,51,52,54,56,57].

TSA indicated that the number of enrolled patients exceeded the RIS for comparing the incidence of PON between the tropisetron group and the control group (1754 of 992 patients) (Figure 2B, Table 3) [29,30,32,33,35,40,42,48,49,50,51,56,57] when including studies comparing dexamethasone to tropisetron combined with dexamethasone (2312 of 801 patients) (Supplementary Figure S2, Table 3) [35,52,54]. The cumulative Z-curve exceeded both the conventional test and trial sequential monitoring boundaries for comparing the incidence of PON between the tropisetron group and the control group (Figure 2B, Table 3) [29,30,32,33,35,40,42,48,49,50,51,56,57] when including studies comparing dexamethasone to tropisetron combined with dexamethasone [35,52,54] (Supplementary Figure S2, Table 3) [29,30,32,33,35,40,42,48,49,50,51,52,54,56,57].

No significant difference in terms of the incidence of PON was observed between tropisetron and metoclopramide (n = 1, RR: 0.786; 95% CI 0.407–1.516; NNTB: 13; 95% CI NNTH 8 to ∞ to NNTB 4) (Supplementary Figure S3, Table 3) [29], ondansetron (n = 4, RR: 0.981; 95% CI 0.828–1.163; I^2^ = 0.00; P_chi_^2^ = 0.684; τ = 0.00, NNTH: 552; 95% CI NNTH 17 to ∞ to NNTB 18) (Supplementary Figure S4, Table 3) [42,47,48,49], ondansetron when including studies comparing tropisetron combined with droperidol to ondansetron combined with droperidol [39] (n = 5, RR: 1.016; 95% CI 0.865–1.194; I^2^ = 0.00; P_chi_^2^ = 0.548; τ = 0.00, NNTH: 84; 95% CI NNTH 15 to ∞ to NNTB 23) (Supplementary Figure S5, Table 3) [39,42,47,48,49], dexamethasone (n = 4, RR: 0.994; 95% CI 0.718–1.375; I^2^ = 16.00; P_chi_^2^ = 0.312; τ = 0.137; 95% PI 0.699–2.273; NNTH: 273; 95% CI NNTH 11 to ∞ to NNTB 12) (Supplementary Figure S6, Table 3) [35,50,51,54], droperidol (n = 1, RR: 0.892; 95% CI 0.658–1.208; NNTB: 15; 95% CI NNTH 9 to ∞ to NNTB 4) (Supplementary Figure S7, Table 3) [37], and granisetron (n = 2, RR: 1.296; 95% CI 0.816–2.057; I^2^ = 0.00; P_chi_^2^ = 0.472; τ = 0.00; NNTH: 22; 95% CI NNTH 8 to ∞ to NNTB 29) (Supplementary Figure S8, Table 3) [42,47].

TSA indicated that only 63.1% (984 of 1559 patients), 69.7% (1072 of 1538 patients), 20.2% (408 of 2023 patients), and 8.2% (338 of 4106 patients) of the RIS was accrued for comparing the incidence of PON between tropisetron and ondansetron (Supplementary Figure S9, Table 3), ondansetron when including studies comparing tropisetron combined with droperidol to ondansetron combined with droperidol (Koivuranta, 1999) (Supplementary Figure S10, Table 3), dexamethasone (Supplementary Figure S11, Table 3), and granisetron (Supplementary Figure S12, Table 3) [42,47], respectively.

The cumulative Z-curve (represented by the complete blue curve) exceeded the futility boundary (represented by the complete red curve on the right side) for comparing the incidence of PON between tropisetron and ondansetron (Supplementary Figure S9, Table 3) [42,47,48,49] and ondansetron when including studies comparing tropisetron combined with droperidol to ondansetron combined with droperidol [39] (Supplementary Figure S10, Table 3) [39,42,47,48,49]. However, the cumulative Z-curve did not exceed the conventional test boundary (represented by the dotted red line) or trial sequential monitoring boundary (represented by the complete red curve) for comparing the incidence of PON between tropisetron and dexamethasone (Supplementary Figure S11, Table 3) [35,50,51,54] and granisetron (Supplementary Figure S12, Table 3) [42,47].

#### 3.4.2. Post-Operative Vomiting

**Figure 3:**
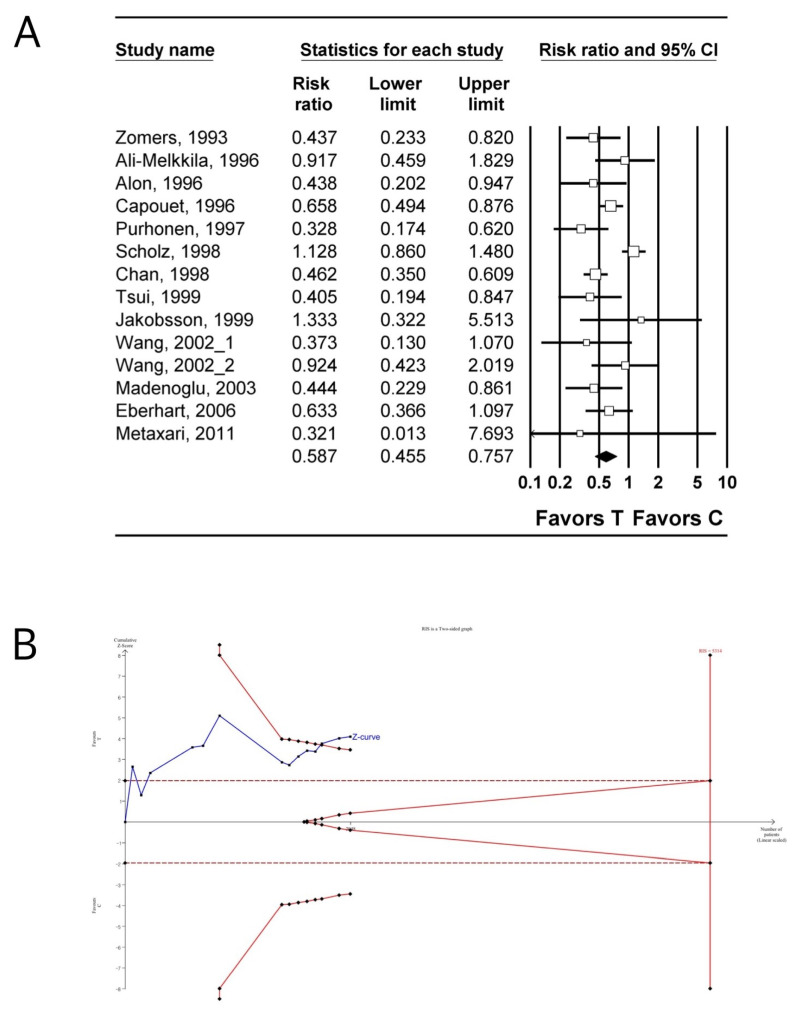
Incidence of post-operative vomiting in the tropisetron and control groups. (**A**) Forest plot. The figure depicts individual trials as filled squares, with the relative sample size and 95% confidence interval (CI) of the difference represented as a solid line. The diamond shape represents the pooled estimate and uncertainty for the combined effect. The pooled estimate indicates that the incidence of post-operative vomiting in the tropisetron group is lower than that in the control group [29,30,32,33,35,40,42,46,48,49,50,51,56,57]. (**B**) Trial sequential analysis plot. The uppermost and lowermost complete red curves represent the trial sequential monitoring boundary lines for benefit and harm, respectively. The horizontal dotted red line represents the conventional boundaries for statistical significance. The triangular red lines on the right side represent the futility boundaries. The blue solid line represents the cumulative z-curve. The number on the *x*-axis indicates the required information size (n = 5314). The cumulative Z-curve exceeds the conventional and trial sequential monitoring boundaries, indicating a statistically significant difference favoring the use of tropisetron to reduce the incidence of post-operative vomiting before reaching the required information size.

The incidence of POV was investigated in 14 studies (2048 patients) [29,30,32,33,35,40,42,46,48,49,50,51,56,57]. The incidence of POV in the tropisetron group was significantly lower than that in the control group (n = 14, RR: 0.587; 95% CI 0.455–0.757; I^2^ = 63.32; P_chi_^2^ = 0.001; τ = 0.346, 95% PI 0.415–0.829; NNTB: 11; 95% CI NNTB 7 to NNTB 19) (Figure 3A, Table 3). The incidence of POV in the tropisetron group continued to be significantly lower than that in the control group even after the inclusion of studies that compared droperidol to tropisetron combined with droperidol [55] and those that compared dexamethasone to tropisetron combined with dexamethasone [35,52,54] (n = 18, RR: 0.592; 95% CI 0.479–0.732; I^2^ = 53.41; P_chi_^2^ = 0.004; τ = 0.300, 95% PI 0.439–0.799; NNTB: 10 95% CI NNTB 7 to NNTB 15) (Supplementary Figure S13, Table 3) [29,30,32,33,35,40,42,46,48,49,50,51,52,54,55,56,57].

TSA indicated that only 38.5% (2048 of 5314 patients) and 63.0% (2487 of 3947 patients) of the RIS was accrued for comparing the incidence of POV between tropisetron and control groups (Figure 3B, Table 3) [29,30,32,33,35,40,42,46,48,49,50,51,56,57], when including the studies comparing droperidol to tropisetron combined with droperidol [55], and when comparing dexamethasone to tropisetron combined with dexamethasone [35,52,54] (Supplementary Figure S14, Table 3) [29,30,32,33,35,40,42,46,48,49,50,51,52,54,55,56,57], respectively. The cumulative Z-curve exceeded both the conventional test and trial sequential monitoring boundaries for comparing the incidence of POV between tropisetron and control groups (Figure 3B, Table 3) when including studies comparing droperidol to tropisetron combined with droperidol [55] and dexamethasone to tropisetron combined with dexamethasone [35,52,54]) (Supplementary Figure S14, Table 3) [29,30,32,33,35,40,42,46,48,49,50,51,52,54,55,56,57].

The incidence of POV in the tropisetron group was lower than that in the droperidol group (n = 2, RR: 0.400; 95% CI 0.260–0.614; I^2^ = 0.00; P_chi_^2^ = 0.864; τ = 0.00; NNTB: 4; 95% CI NNTB 3 to NNTB 9) (Supplementary Figure S15, Table 3) [37,46]. However, no statistically significant difference was observed between tropisetron and metoclopramide (n = 1, RR: 1.833; 95% CI 0.751–4.478; NNTH: 8; 95% CI NNTH 3 to ∞ to NNTB 19) (Supplementary Figure S16, Table 3) [29], ondansetron (n = 6, RR: 0.940; 95% CI 0.649–1.361; I^2^ = 25.32; P_chi_^2^ = 0.244; τ = 0.233, 95% PI 0.611–1.636; NNTB: 2405; 95% CI NNTH 17 to ∞ to NNTB 16) (Supplementary Figure S17, Table 3) [31,42,47,48,49,50], ondansetron when including studies comparing tropisetron combined with droperidol to ondansetron combined with droperidol [39] (n = 7, RR: 0.989; 95% CI 0.742–1.319; I^2^ = 10.40; P_chi_^2^ = 0.350; τ = 0.138, 95% PI 0.136–7.166; NNTH: 161; 95% CI NNTH 20 to ∞ to NNTB 26) (Supplementary Figure S18, Table 3) [31,39,42,47,48,49,50], dexamethasone (n = 3, RR: 1.286; 95% CI 0.803–2.058; I^2^ = 16.30; Pchi^2^ = 0.303; τ = 0.173, 95% PI 0.776–2.13; NNTH: 18; 95% CI NNTH 7 to ∞ to NNTB 30) (Supplementary Figure S19, Table 3) [35,51,54], granisetron (n = 2, RR: 1.594; 95% CI 0.757–3.358; I^2^ = 0.00; Pchi^2^ = 0.910; τ = 0.00; NNTH: 29; 95% CI NNTH 11 to ∞ to NNTB 45) (Supplementary Figure S20, Table 3) [42,47], and granisetron when including studies comparing tropisetron combined with droperidol to granisetron combined with droperidol [55] (n = 3, RR: 1.404; 95% CI 0.830–2.376; I^2^ = 0.00; Pchi^2^ = 0.0.889; τ = 0.00; NNTB: 3; 95% CI NNTB 2 to NNTB 6) (Supplementary Figure S21, Table 3) [42,47,55].

TSA indicated that only 19.8% (1120 of 5659 patients), 24.4% (1208 of 4955 patients), 8.1% (332 of 4120 patients), 26.5% (217 of 818 patients), 6.8% (338 of 4955 patients), and 6.1% (423 of 6880 patients) of the RIS was accrued for comparing the incidence of POV between tropisetron and ondansetron (Supplementary Figure S22, Table 3) [31,42,47,48,49,50], ondansetron when including studies comparing tropisetron combined with droperidol to ondansetron combined with droperidol [39] (Supplementary Figure S23, Table 3) [31,39,42,47,48,49,50], dexamethasone (Supplementary Figure S24, Table 3) [35,51,54], droperidol (Supplementary Figure S25, Table 3) [37,46], granisetron (Supplementary Figure S26, Table 3) [42,47], and granisetron when including studies comparing tropisetron combined with droperidol to granisetron combined with droperidol [55] (Supplementary Figure S27, Table 3) [42,47,55], respectively.

The cumulative Z-curve (represented by the complete blue curve) did not exceed the conventional test (represented by the dotted red line) or trial sequential monitoring (represented by the complete red curve) boundaries for comparing the incidence of POV between tropisetron and ondansetron (Supplementary Figure S22, Table 3) [31,42,47,48,49,50], ondansetron when including studies comparing tropisetron combined with droperidol to of ondansetron combined with droperidol [39] (Supplementary Figure S23, Table 3) [31,39,42,47,48,49,50], dexamethasone (Supplementary Figure S24, Table 3) [35,51,54], granisetron (Supplementary Figure S26, Table 3) [42,47], and granisetron when including studies comparing tropisetron combined with droperidol to granisetron combined with droperidol [55] (Supplementary Figure S27, Table 3) [42,47,55].

However, when comparing between tropisetron and droperidol, the cumulative Z-curve for the incidence of POV (represented as the complete blue curve) exceeded the conventional test boundary (represented by the dotted red line) and met the trial sequential monitoring boundary (represented by the complete red curve) (Supplementary Figure S25, Table 3) [31,42,47,48,49,50].

#### 3.4.3. Post-Operative Nausea and Vomiting

**Figure 4:**
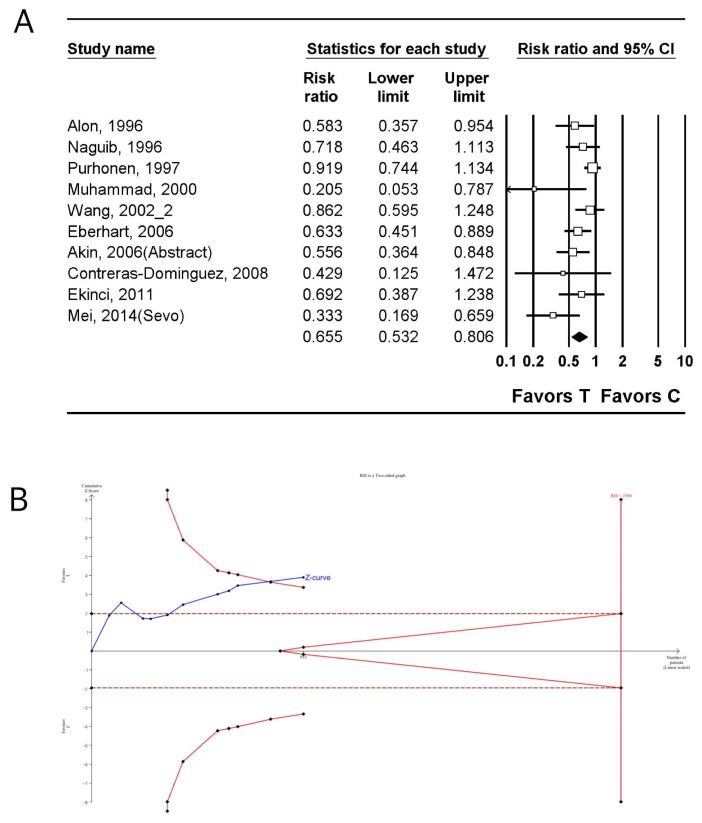
Incidence of post-operative nausea and vomiting in the tropisetron and control groups. (**A**) Forest plot. The figure depicts individual trials as filled squares, with the relative sample size and 95% confidence interval (CI) of the difference represented as a solid line. The diamond shape represents the pooled estimate and uncertainty for the combined effect. The pooled estimate indicates that the incidence of post-operative nausea and vomiting in the tropisetron group is lower than that in the control group [28,30,34,35,36,41,43,44,46,51]. (**B**) Trial sequential analysis plot. The uppermost and lowermost complete red curves represent the trial sequential monitoring boundary lines for benefit and harm, respectively. The horizontal dotted red line represents the conventional boundaries for statistical significance. The triangular red lines on the right side represent the futility boundaries. The blue solid line represents the cumulative z-curve. The number on the *x*-axis indicates the required information size (n = 2380). The cumulative Z-curve exceeds the conventional and trial sequential monitoring boundaries, favoring the use of tropisetron over that of the control for the prevention of the incidence of post-operative nausea and vomiting before reaching the required information size.

The incidence of PONV was investigated in 10 studies (953 patients) [28,30,32,35,36,41,43,44,46,51]. The incidence of PONV in the tropisetron group was significantly lower than that in the control group (n = 11, RR: 0.655; 95% CI 0.532–0.806; I^2^ = 49.09; τ = 0.218, 95% PI 0.440–0.975; NNTB: 5; 95% CI NNTB 4 to NNTB 7) (Figure 4A, Table 3). The incidence of PONV in the tropisetron group continued to be significantly lower than that in the control group (n = 15, RR: 0.614; 95% CI 0.504–0.749; I^2^ = 59.21; Pchi^2^ = 0.002; τ = 0.280, 95% PI 0.375–1.004; NNTB: 5 95% CI NNTB 4 to NNTB 7) (Supplementary Figure S28, Table 3), even after the inclusion of studies that compared dexamethasone to tropisetron combined with dexamethasone [35,52,53] and those that compared metoclopramide to tropisetron combined with metoclopramide [28,30,32,35,36,41,43,44,45,46,51,52,53].

TSA indicated that only 40.0% (953 of 2380 patients) and 69.7% (1428 of 2048 patients) of the RIS was accrued for comparing the incidence of PONV between tropisetron and control groups (Figure 4B, Table 3) [28,30,32,35,36,41,43,44,46,51] and tropisetron and control group when including studies comparing dexamethasone to tropisetron combined with dexamethasone [35,52,53] and metoclopramide to tropisetron combined with metoclopramide [45] (Supplementary Figure S29, Table 3) [28,30,32,35,36,41,43,44,45,46,51,52,53], respectively. The cumulative Z-curve exceeded both the conventional test and trial sequential monitoring boundaries for comparing the incidence of PONV between tropisetron and control group (Figure 4B, Table 3) [28,30,32,35,36,41,43,44,46,51] and tropisetron and control group when including studies comparing to tropisetron combined with dexamethasone [35,52,53], and metoclopramide to tropisetron combined with metoclopramide [45] (Supplementary Figure S29, Table 3) [28,30,32,35,36,41,43,44,45,46,51,52,53].

The incidence of PONV in the tropisetron group was lower than that in the metoclopramide group (n = 5; RR: 0.770; 95% CI 0.624–0.950; I^2^ = 0.00; Pchi^2^ = 0.419; τ = 0.00; 95% PI 0.440–0.975; NNTB: 7; 95% CI NNTB 4 to NNTB 32) (Supplementary Figure S30, Table 3) [34,36,38,43,44]. However, no statistically significant difference was observed between tropisetron and ondansetron (n = 3; RR: 1.063; 95% CI 0.727–1.553; I^2^ = 38.38; τ = 0.209; Pchi^2^ = 0.047; 95% PI 0.564–2.001; NNTH: 93; 95% CI NNTH 7 to ∞ to NNTB 8) (Supplementary Figure S31, Table 3) [36,38,44], dexamethasone (n = 3; RR: 1.129; 95% CI 0.552–2.306; I^2^ = 70.56; τ = 0.514, Pchi^2^ = 0.033; 95% PI 0.252–5.061; NNTH: 21; 95% CI NNTH 6 to ∞ to NNTB 15) (Supplementary Figure S32, Table 3) [34,35,51], droperidol (n = 3; RR: 0.970; 95% CI 0.787–1.196; I^2^ = 0.0; Pchi^2^ = 0.586; τ = 0.0, NNTB: 194; 95% CI NNTH 7 to ∞ to NNTB 7) (Supplementary Figure S33, Table 3) [34,36,46], droperidol when including studies comparing tropisetron combined with dexamethasone to droperidol combined with dexamethasone [53] (n = 4; RR: 0.981; 95% CI 0.812–1.185; I^2^ = 0.0; Pchi^2^ = 0.771; τ = 0.0, NNTB: 294; 95% CI NNTH 9 to ∞ to NNTB 8) (Supplementary Figure S34, Table 3) [34,36,46,53], and granisetron (n = 2; RR: 1.073; 95% CI 0.637–1.806; I^2^ = 0.0; Pchi^2^ = 0.922; τ = 0.0, NNTH: 50; 95% CI NNTH 5 to ∞ to NNTB 6) (Supplementary Figure S35, Table 3) [34,44].

TSA indicated that only 46.9% (290 of 618 patients), 4.7% (214 of 4576 patients), 6.1% (282 of 7432 patients), 25.9% (187 of 721 patients), 36.1% (285 of 789 patients), and 12.7% (100 of 1719 patients) of the RIS was accrued for comparing the incidence of PONV between tropisetron and metoclopramide (Supplementary Figure S36, Table 3) [34,36,38,43,44], ondansetron (Supplementary Figure S37, Table 3) [36,38,44], dexamethasone (Supplementary Figure S38, Table 3) [34,35,51], droperidol (Supplementary Figure S39, Table 3) [34,36,46], droperidol when including studies comparing tropisetron combined with dexamethasone to droperidol combined with dexamethasone [53] (Supplementary Figure S40, Table 3) [34,36,46,53], and granisetron (Supplementary Figure S41, Table 3) [34,44], respectively.

The cumulative Z-curve (represented by the complete blue curve) exceeded the conventional test boundary (represented by the dotted red line) but not the trial sequential monitoring boundary (represented by the complete red curve) for comparing the incidence of PONV between tropisetron and metoclopramide (Supplementary Figure S36, Table 3) [34,36,38,43,44].

However, the cumulative Z-curve (represented by the complete blue curve) did not exceed the conventional test (represented by the dotted red line) or trial sequential monitoring (represented by the complete red curve) boundaries for comparing the incidence of PONV between tropisetron and ondansetron (Supplementary Figure S37, Table 3) [36,38,44], dexamethasone (Supplementary Figure S38, Table 3) [34,35,51], droperidol (Supplementary Figure S39, Table 3) [34,36,46], droperidol when including studies comparing tropisetron combined with dexamethasone to droperidol combined with dexamethasone [53] (Supplementary Figure S40, Table 3) [34,36,46,53], and granisetron (Supplementary Figure S41, Table 3) [34,44].

#### 3.4.4. Use of Rescue Anti-Emetic Agents

**Figure 5:**
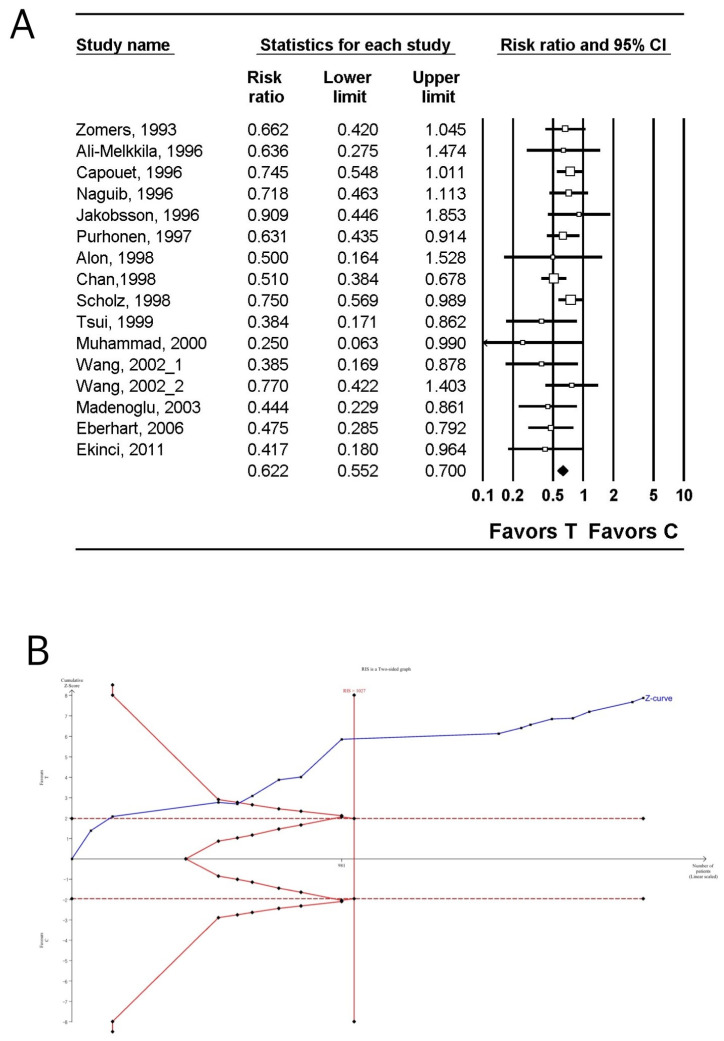
Use of rescue anti-emetic agents in the tropisetron and control groups. (**A**) Forest plot. The figure depicts individual trials as filled squares, with the relative sample size and 95% confidence interval (CI) of the difference represented as a solid line. The diamond shape represents the pooled estimate and uncertainty for the combined effect. The pooled estimate indicates that the use of rescue anti-emetic agents in the tropisetron group is lower than that in the control group [29,30,32,33,35,36,40,43,44,46,48,49,50,51,56,57]. (**B**) Trial sequential analysis plot. The uppermost and lowermost complete red curves represent the trial sequential monitoring boundary lines for benefit and harm, respectively. The horizontal dotted red line represents the conventional boundaries for statistical significance. The triangular red lines on the right side represent the futility boundaries. The blue solid line represents the cumulative z-curve. The number on the *x*-axis indicates the required information size (n = 1027). The cumulative Z-curve exceeded the conventional and trial sequential monitoring boundaries, favoring the use of tropisetron over that of the control to reduce the use of rescue anti-emetic agents.

The incidence of use of RA was investigated in 16 studies (2078 patients) [29,30,32,33,35,36,40,43,44,46,48,49,50,51,56,57]. The incidence of use of RA in the tropisetron group was significantly lower than that in the control group (n = 16, RR: 0.622; 95% CI 0.552–0.700; I^2^ = 0.00; Pchi^2^ = 0.492; τ = 0.00; NNTB: 6; 95% CI NNTB 5 to NNTB 9) (Figure 5A, Table 3) [29,30,32,33,35,36,40,43,44,46,48,49,50,51,56,57]. The incidence of use of RA in the tropisetron group continued to be significantly lower than that in the control group (n = 21, RR: 0.620; 95% CI 0.556–0.692; I^2^ = 0.00_hi_^2^ = 0.456; τ = 0.00; NNTB: 10; 95% CI NNTB 7 to NNTB 16) (Supplementary Figure S42, Table 3) even after the inclusion of studies that compared metoclopramide to tropisetron combined with metoclopramide [45], dexamethasone to tropisetron combined with dexamethasone [35,52,54], and droperidol to tropisetron combined with droperidol [29,30,32,33,35,36,40,43,44,45,46,48,49,50,51,52,54,55,56,57].

TSA indicated that the enrolled patients exceed the RIS for comparing the incidence of use of RA between tropisetron and control groups (2078 of 1027 patients) (Figure 5B, Table 3) [29,30,32,33,35,36,40,43,44,46,48,49,50,51,56,57]. Only 60.8% (2738 of 4506) of the RIS was accrued for comparing the incidence of use RA between tropisetron and control group when including studies comparing metoclopramide to tropisetron combined with metoclopramide [45], dexamethasone to tropisetron combined with dexamethasone [35,52,54], and droperidol to tropisetron combined with droperidol [55] (Supplementary Figure S43, Table 3) [29,30,32,33,35,36,40,43,44,45,46,48,49,50,51,52,54,55,56,57]. The cumulative Z-curve exceeded both the conventional test and trial sequential monitoring boundaries curve for comparing the incidence use of RA between tropisetron and control groups (Figure 5B, Table 3) [29,30,32,33,35,36,40,43,44,46,48,49,50,51,56,57], tropisetron and control groups when including studies comparing metoclopramide to tropisetron combined with metoclopramide [45], dexamethasone to tropisetron combined with dexamethasone [35,52,54], and droperidol to tropisetron coimbined with droperidol [55] (Supplementary Figure S43, Table 3) [29,30,32,33,35,36,40,43,44,45,46,48,49,50,51,52,54,55,56,57].

The incidence of use of RA in the tropisetron group was significantly lower than that in the metoclopramide group (n = 5, RR: 0.707; 95% CI 0.561–0.892; I^2^ = 0.0; Pchi^2^ = 0.618; τ = 0.0, NNTB: 7; 95% CI NNTH 4 to NNTB 24) (Supplementary Figure S44, Table 3) [29,36,38,43,44]. However, no statistically significant difference was observed between tropisetron and ondansetron (n = 6; RR: 0.968; 95% CI 0.759–1.235; I^2^ = 18.02; Pchi^2^ = 0.295; τ = 0.130; 95% PI 0.744–1.256; NNTB: 123; 95% CI NNTH 20 to ∞ to NNTB 15) (Supplementary Figure S45, Table 3) [31,36,38,44,48], ondansetron when including studies comparing tropisetron combined with droperidol to ondansetron combined with droperidol [39] (n = 7; RR: 1.000; 95% CI 0.806–1.242; I^2^ = 13.46; Pchi^2^ = 0.327; τ = 0.130; 95% PI 0.799–1.352; NNTB: 1608; 95% CI NNTH 18 to ∞ to NNTB 18) (Supplementary Figure S46, Table 3) [31,36,38,39,44,48], dexamethasone (n = 4; RR: 0.988; 95% CI 0.594–1.645; I^2^ = 48.78; Pchi^2^ = 0.434; τ = 0.358; 95% PI 0.426–2.293; NNTB: 51; 95% CI NNTH 16 to ∞ to NNTB 10) (Supplementary Figure S47, Table 3) [35,50,51,54], droperidol (n = 3, RR: 0.864; 95% CI 0.654–1.142; I^2^ = 0.00; Pchi^2^ = 0.809; τ = 0.00, NNTB: 17; 95% CI NNTH 16 to ∞ to NNTB 6) (Supplementary Figure S48, Table 3) [36,37,46], granisetron (n = 1; RR: 1.083; 95% CI 0.622–1.888; NNTH: 25; 95% CI NNTH 3 to ∞ to NNTB 4) (Supplementary Figure S49, Table 3) [44], and granisetron when including studies comparing the tropisetron combined with droperidol to granisetron combined with droperidol [55] (n = 2; RR: 1.250; 95% CI 0.871–1.795; I^2^ = 0.00; Pchi^2^ = 0.505; τ = 0.00; NNTH: 25; 95% CI NNTH 3 to ∞ to NNTB 4) (Supplementary Figure S50, Table 3) [44,55].

TSA indicated that only 41.6% (320 of 769 patients), 31.4% (917 of 2925 patients), 34.4% (1005 of 2541 patients), 16.1% (408 of 4644 patients), 28.2% (257 of 910 patients), and 14.8% (135 of 1071 patients) of the RIS was accrued for comparing the incidence of use of RA between tropisetron and metoclopramide (Supplementary Figure S51, Table 3) [29,36,38,43,44], ondansetron (Supplementary Figure S52, Table 3) [31,36,38,44,48], ondansetron when including studies comparing tropisetron combined with droperidol to ondansetron cdroperidol [39] (Supplementary Figure S53, Table 3) [31,36,38,39,44,48], dexamethasone (Supplementary Figure S54, Table 3) [35,50,51,54], droperidol (Supplementary Figure S55, Table 3) [36,37,46], and granisetron when including studies comparing tropisetron combined with droperidol to granisetron combined with droperidol [55] (Supplementary Figure S56, Table 3) [44,55], respectively.

The cumulative Z-curve (represented by the complete blue curve) exceeded the conventional test boundary (indicated by the dotted red line) but not the trial sequential monitoring boundary (represented by the complete red curve) for comparing the incidence of use of RA between tropisetron and metoclopramide (Supplementary Figure S51, Table 3) [29,36,38,43,44]. However, the cumulative Z-curve (represented by the complete blue curve) did not exceed the conventional test (represented by the dotted red line) or trial sequential monitoring (represented by the complete red curve) boundaries for comparing the incidence of use of RA between tropisetron and ondansetron (Supplementary Figure S52, Table 3) [31,36,38,44,48], ondansetron when including studies comparing the combination of tropisetron and droperidol with the combination of ondansetron and droperidol [39] (Supplementary Figure S53, Table 3) [31,36,38,39,44,48], dexamethasone (Supplementary Figure S54, Table 3) [35,50,51,54], droperidol (Supplementary Figure S55, Table 3) [36,37,46], and granisetron when including studies comparing tropisetron combined with droperidol and granisetron combined with droperidol [55] (Supplementary Figure S56, Table 3) [44,55].

#### 3.4.5. Complete Response

**Figure 6:**
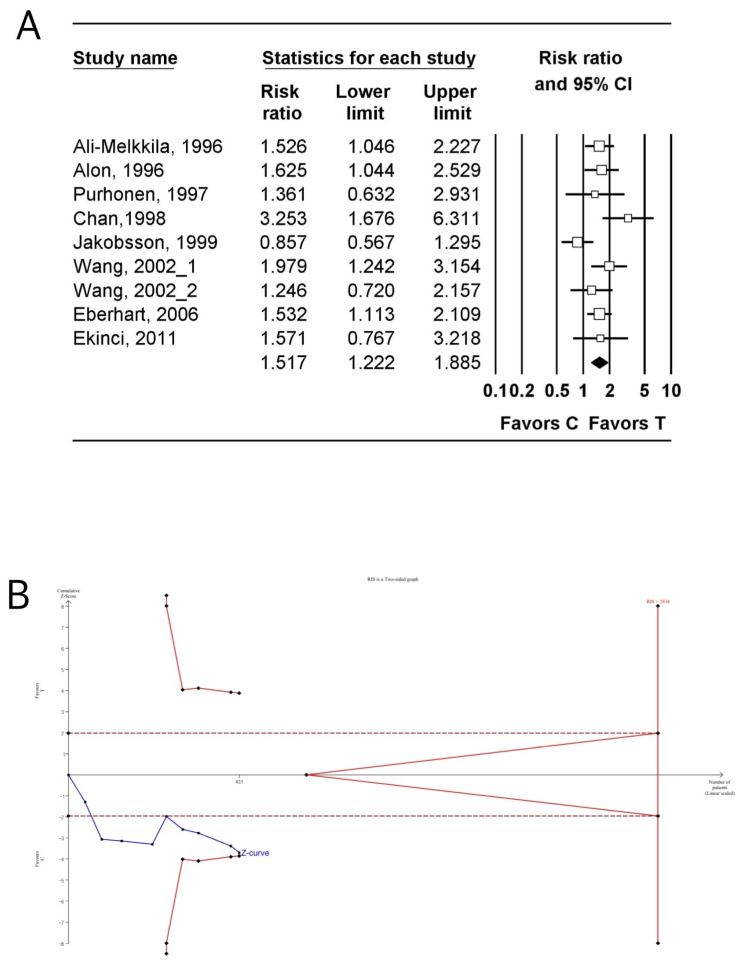
The incidence of complete response in the tropisetron and control groups. (**A**) Forest plot. The figure depicts individual trials as filled squares, with the relative sample size and 95% confidence interval (CI) of the difference as a solid line. The diamond shape represents the pooled estimate and uncertainty for the combined effect. The pooled estimate indicates that the incidence of complete response in the tropisetron group is lower than that in the control group [29,30,33,35,36,46,50,51,56]. (**B**) Trial sequential analysis plot. The uppermost and lowermost complete red curves represent the trial sequential monitoring boundary lines for benefit and harm, respectively. The horizontal dotted red line represents the conventional boundaries for statistical significance. The triangular red lines on the right side represent the futility boundaries. The blue solid line represents the cumulative z-curve. The number on the *x*-axis indicates the required information size (n = 2838). The cumulative Z-curve exceeded the conventional boundary but not the trial sequential monitoring boundary, favoring the use of tropisetron over that of the control for complete response.

The incidence of CR was investigated in nine studies (728 patients) [29,30,33,35,36,46,50,51,56]. The incidence of CR in the tropisetron group was significantly higher than that in the control group (n = 9; RR: 1.517; 95% CI 1.222–1.885; I^2^ = 44.14; Pchi^2^ = 0.074; τ = 0.215; 95% PI 1.018–2.260; NNTB: 1; 95% CI NNTB 1 to NNTB 1) (Figure 6A, Table 3) [29,30,33,35,36,46,50,51,56]. The incidence of CR in the tropisetron group continued to be significantly higher than that in the control group (n- = 12, 1.442; 95% CI 1.250–1.663; I^2^ = 30.00; Pchi^2^ = 0.152; τ = 0.133; 95% PI 1.133–1.834; NNTB: 5 95% CI NNTB 4 to NNTB 8) (Supplementary Figure S57, Table 3) even after the inclusion of studies comparing dexamethasone to tropisetron combined with dexamethasone [29,30,33,35,36,46,50,51,53,54,56].

TSA indicated that only 29.0% (823 of 2838 patients) and 63.0% (1175 of 1864 patients) of the RIS was accrued for comparing the incidence of CR between tropisetron and control groups (Figure 6B, Table 3) [29,30,33,35,36,46,50,51,56] and tropisetron and control groups when including studies comparing dexamethasone to tropisetron combined with dexamethasone [35,53,54] (Supplementary Figure S58, Table 3) [29,30,33,35,36,46,50,51,53,54,56], respectively. The cumulative Z-curve exceeded the conventional test boundary but not the trial sequential monitoring boundary for comparing the incidence of CR between tropisetron and control groups (Figure 6B, Table 3) [29,30,33,35,36,46,50,51,56]. The cumulative Z-curve exceeded both the conventional test and trial sequential monitoring boundaries for comparing the incidence of CR between tropisetron and control groups when including studies comparing dexamethasone to tropisetron combined with dexamethasone [35,53,54] (Supplementary Figure S58, Table 3) [29,30,33,35,36,46,50,51,53,54,56].

No statistically significant difference in terms of the incidence of CR was observed between tropisetron and metoclopramide (n = 3; RR: 1.180; 95% CI 0.933–1.493; I^2^ = 0.00; Pchi^2^ = 0.377; τ = 0.00; NNTB: 8; 95% CI NNTH 85 to ∞ to NNTB 4) (Supplementary Figure S59, Table 3) [29,36,38], ondansetron (n = 4; RR: 0.928; 95% CI 0.726–1.185; I^2^ = 41.88; Pchi^2^ = 0.160; τ = 0.162; 95% PI 0.635–1.356; NNTH: 41; 95% CI NNTH 9 to ∞ to NNTB 15) (Supplementary Figure S60, Table 3) [36,38,44,47], dexamethasone (n = 4; RR: 0.939 CI 0.747–1.181; I^2^ = 56.21; Pchi^2^ = 0.077; τ = 0.174; 95% PI 0.625–1.412; NNTH: 31; 95% CI NNTH 8 to ∞ to NNTB 16) (Supplementary Figure S61, Table 3) [35,50,51,54], droperidol (n = 3; RR: 1.142; 95% CI 0.836–1.558; I^2^ = 0.00; Pchi^2^ = 0.982; τ = 0.00; NNTB: 20; 95% CI NNTH 15 to ∞ to NNTB 6) (Supplementary Figure S62, Table 3) [36,37,46], droperidol when including studies comparing tropisetron combined with dexamethasone to droperidol combined with dexamethasone [53] (n = 4; RR: 1.075;95% CI 0.850–1.359; I^2^ = 0.00; Pchi^2^ = 0.946; τ = 0.00; NNTB: 26; 95% CI NNTH 16 to ∞ to NNTB 7) (Supplementary Figure S63, Table 3) [36,37,46,53], and granisetron (n = 2; RR: 0.934;95% CI 0.798–1.093; I^2^ = 0.00; Pchi^2^ = 0.742; τ = 0.00; NNTH: 20; 95% CI NNTH 6 to ∞ to NNTB 17) (Supplementary Figure S64, Table 3) [44,47].

TSA indicated that only 21.9% (239 of 1093 patients), 29.1% (452 of 1551 patients), 42.0% (408 of 971 patients), 17.9% (257 of 1436 patients), 24.7% (355 of 1140 patients), and 39.3% (286 of 728 patients) of the RIS was accrued for comparing the incidence of CR between tropisetron and metoclopramide (Supplementary Figure S65, Table 3) [29,36,38], ondansetron (Supplementary Figure S66, Table 3) [36,38,44,47], dexamethasone (Supplementary Figure S67, Table 3) [35,50,51,54], droperidol (Supplementary Figure S68, Table 3) [36,37,46], droperidol when including studies comparing tropisetron combined with dexamethasone to droperidol combined with dexamethasone [53] (Supplementary Figure S69, Table 3) [36,37,46,53], and granisetron (Supplementary Figure S70, Table 3) [44,47].

The cumulative Z-curve (represented by the complete blue curve) did not exceed the conventional test (represented by the dotted red line) or trial sequential monitoring (represented by the complete red curve) boundaries for comparing the incidence of CR between tropisetron and metoclopramide (Supplementary Figure S65, Table 3) [29,36,38], ondansetron (Supplementary Figure S66, Table 3) [36,38,44,47], dexamethasone (Supplementary Figure S67, Table 3) [35,50,51,54], droperidol (Supplementary Figure S68, Table 3) [36,37,46], droperidol when including studies comparing tropisetron combined with dexamethasone to droperidol combined with dexamethasone [53] (Supplementary Figure S69, Table 3) [36,37,46,53], and granisetron (Supplementary Figure S70, Table 3) [44,47].

### 3.5. Sensitivity Analysis

Sensitivity analysis, performed by removing one study at a time, revealed no statistically significant difference between tropisetron and control groups in terms of the incidence of PON, POV, PONV, use of RA, and CR. However, the exclusion of the studies conducted by Capouet et al. [32], Chan et al. [33], and Ebehart et al. [35] resulted in a statistically significant difference between the tropisetron and control groups in terms of the incidence of PON when including studies [35,52,54] comparing dexamethasone to tropisetron combined with dexamethasone [29,30,32,33,35,40,42,48,49,50,51,52,54,56,57] (Table 3). Furthermore, there was no statistically significant difference in terms of the incidence of PON, POV, PONV, use of RA, and CR between tropisetron and other anti-emetic agents. However, statistically significant difference in terms of the incidence of PONV was observed between tropisetron and metoclopramide when excluding studies conducted by Naguib et al. [44] and Jokela et al. [34,36,38,43,44] (Table 3).

### 3.6. Publication Bias

Begg’s funnel plot and Egger’s linear regression test revealed no evidence of publication bias for the following outcomes: the incidence of PON compared with that in the control group (*p* = 0.21605, Supplementary Figure S71) and with that in the control group when including studies comparing dexamethasone to tropisetron combined with dexamethasone (*p* = 0.48744, Supplementary Figure S72); the incidence of POV compared with that in the control group (*p* = 0.62222, Supplementary Figure S73) and with that in the control group when including studies comparing droperidol to tropisetron combined with droperidol and those comparing dexamethasone to tropisetron combined with dexamethasone (*p* = 0.27420, Supplementary Figure S74); and the incidence of CR compared with that in the control group when including studies comparing dexamethasone to tropisetron combined with dexamethasone (*p* = 0.27420, Supplementary Figure S75).

Begg’s funnel plot and Egger’s linear regression test indicated the possibility of the inclusion of publication bias for the following outcomes: (1) the incidence of PONV compared with that in the control group (*p* = 0.00312, Supplementary Figure S76) and with that in the control group when including studies comparing dexamethasone to tropisetron combined with dexamethasone and those comparing metoclopramide to tropisetron combined with metoclopramide (*p* = 0.00002, Supplementary Figure S77); (2) the incidence of use of RA compared with that in the control group (*p* = 0.06786, Supplementary Figure S78) and with that in the control group when including studies comparing metoclopramide to tropisetron combined with metoclopramide, those comparing dexamethasone to tropisetron combined with dexamethasone, and those comparing droperidol to tropisetron combined with droperidol (*p* = 0.02875, Supplementary Figure S79). A subsequent trim-and-fill analysis to assess publication bias, however, did not reveal any significant changes in the findings.

### 3.7. Quality of the Evidence

Twenty-eight outcomes were evaluated using the GRADE system (Table 4). The quality of the pooled analysis was high for the following outcomes: the incidence of PON compared with that in the control group, the incidence of PONV compared with that in the control group, the incidence of use of RA compared with that in the control group and with that in the control group when including studies comparing other anti-emetic agents to tropisetron combined with other anti-emetic agents, and the incidence of CR compared with that in the control group and with that in the control group when including studies comparing other anti-emetic agents to tropisetron combined with other anti-emetic agents. However, the quality of the pooled analysis was low for comparing the incidence of PONV and CR between tropisetron and dexamethasone. For the rest of the outcomes, the quality of the pooled analysis was of moderate quality.

## 4. Discussion

This systematic review, which included 30 RCTs involving a total of 5175 patients, demonstrated that the prophylactic administration of tropisetron reduced the incidence of PON (GRADE: High), POV (GRADE: Moderate) and PONV (GRADE: High) and the requirement of RA (GRADE: High) and increased the incidence of CR (GRADE: High).

Conventional meta-analysis showed that the prophylactic administration of tropisetron resulted in a significant reduction in the incidence of PON, POV, and PONV, as well as the incidence of use of RA. The cumulative Z-curves for these outcomes exceeded the conventional test and trial sequential monitoring boundaries, indicating that the TSA results had achieved a sufficient level of evidence and were conclusive. Furthermore, the findings of the current meta-analysis indicated that compared with the administration of placebo agents, the administration of tropisetron resulted in a higher incidence of CR. The cumulative Z-curve for CR exceeded the conventional test boundary; however, it did not exceed the trial sequential monitoring boundary owing to the sparsity of the data. The inclusion of studies comparing other anti-emetic agents and the combination of tropisetron and other anti-emetic agents resulted in no changes (except that the cumulative Z-curve for CR exceeded the trial sequential monitoring boundary).

The effects of other anti-emetic agents did not differ from those of a combination of tropisetron and other anti-emetic agents or a combination of other anti-emetic agents. However, the incidence of POV in the tropisetron group was lower than that in the droperidol group. Furthermore, the incidence of PONV and the incidence of use of RA in the tropisetron group were lower than those in the metoclopramide group in the conventional meta-analysis.

The complex pathophysiology of PONV, which involves numerous pathways and receptors, remains unclear. However, its etiology is considered to be multifactorial and various anti-emetic agents have been used to prevent and treat PONV [1,58,59,60,61]. The area postrema, which serves as the vomiting center and orchestrates emetic responses, is located on the dorsal surface of the medulla oblongata at the caudal end of the fourth ventricle. Diverse pathways, including vagal afferent fibers in the gastrointestinal tract, input from the vestibular system, activation of the chemoreceptor trigger zone, and signaling from the forebrain, can initiate emesis [59].

The 5-HT3 receptors present in the central nervous system, peripheral nervous system, and intestinal tissues play a pivotal role in coordinating emetic processes [62,63]. Therefore, 5-HT3 receptor antagonists which serve as anti-emetic agents by selectively and competitively binding to these receptors to block emetogenic signals are most frequently used anti-emetics to manage PONV. Competitive antagonists targeting serotonin 5-HT_3_ receptors, which were initially used for the prevention of chemotherapy-induced emesis [64], are employed prophylactically and as an RA in the management of PONV. Not only that, but the role of 5-HT_3_ receptor antagonists is expanding as recent evidence show their potential therapeutic effects on neuropsychiatric and gastrointestinal disorders [65,66,67].

Selective 5-HT3 receptor antagonists available in the United States and Europe include dolasetron, granisetron, ondansetron, palonosetron, and tropisetron [68]. Among these, ondansetron was first introduced as a 5-HT3 receptor antagonist. Tropisetron, which was introduced relatively later, shares many similar mechanisms of action. Nevertheless, pharmacokinetic and pharmacodynamic variations contribute to its distinct properties and drug efficacy [69].

Tropisetron exerts additional antagonist effects on the 5-HT_4_ receptors, albeit with low affinity, in addition to exhibiting 5-HT_3_ antagonism; thus, it possesses theoretical advantages as an anti-emetic agent [6]. Certain animal models have implicated the involvement of 5-HT_4_ receptors in emetic mechanisms [70,71]. Tropisetron also exhibits a longer elimination half-life of 6 to 8 h as well as an extended duration of action [72,73], which facilitates prolonged anti-emetic coverage post-operatively. This feature sets it apart from other agents in its class and renders it suitable for use in single-dose PONV prophylaxis regimens [9]. Furthermore, tropisetron can mitigate post-operative cognitive dysfunction [74], chemotherapy-induced peripheral neuropathy [75], and fibromyalgia-associated pain [76].

Numerous systematic reviews and meta-analyses have demonstrated the efficacy of tropisetron in the prevention of the incidence of PONV [14,77,78]. This has led to the inclusion of tropisentron in various clinical guidelines. However, the findings of previous studies are dated and inconsistent, indicating the requirement for updated evidence. This systematic review incorporated recent data and revealed conclusive evidence supporting the efficacy of tropisetron in the prevention of PON, POV, PONV, and RA use compared with that of a placebo. Furthermore, the effects of tropisetron were not inferior to those of other 5-HT_3_ receptor antagonists, such as ondansetron and granisetron, and alternative anti-emetic agents with distinct mechanisms of action. The efficacy of tropisetron surpasses that of droperidol in the prevention of POV and that of metoclopramide in reducing the incidence of PONV and the use of RAs.

This meta-analysis has certain limitations. First, the studies included in the current meta-analysis involved patients who underwent different surgeries of varying durations. Furthermore, the use, type, and dose of anesthetics and analgesics varied across the studies. These variations may have increased the between-study heterogeneity. Second, the cost-effectiveness and major side effects, including headaches, QT prolongation, and clinical outcomes beyond the observed time period (such as readmission due to PONV), could not be analyzed owing to the unavailability of data. Further studies on these outcomes are warranted. Lastly, studies that included pediatric patients were excluded to limit heterogeneity. However, in contrast to adult patients, pediatric patients possess unique characteristics that vary significantly even within the pediatric population. Therefore, further studies must be conducted to confirm the efficacy of tropisetron in the prevention of PONV in pediatric patients. Nevertheless, the findings of this systematic review and meta-analysis with TSA yielded convincing evidence indicating the superior effect of tropisetron in the prevention of episodes of vomiting.

## 5. Conclusions

This systematic review and meta-analysis with trial sequential analysis provides evidence to support that the prophylactic administration of tropisetron is effective in preventing the incidence of PON and PONV, reducing the requirement for the use of RAs, increasing the incidence of CR with high evidence certainty, and preventing POV with moderate evidence certainty, compared to controls. These findings are also supported by TSA.

## Figures and Tables

**Table 1:** Study characteristics of included studies.

Source	No. of Cases (Total)	Age (yrs) Range	Sex (M/F)	Weight (kg)			Height (cm)	ASA-PS	Type of Anesthesia	Duration of Anesthesia (min)		Type of Surgery
Akin, 2006 [28]	35 (105)	19–68	NR	NR			NR	NR	Desflurane with N₂O	NR		Thyroidectomy
Ali-Melkkilä, 1996 [29]	40 (120)	18–75	70/50	P		70.9 ± 14.3	168.2 ± 10.6	I–III	Isoflurane with N₂O	1.82 ± 0.90		Elective ophthalmic surgery
				T		73.4 ± 17.6	170.6 ± 9.9			1.58 ± 0.77		
				M		73.2 ± 12.6	170.9 ± 10.2			1.78 ± 1.66		
Alon, 1996 [30]	40 (80)	17–72	0/80	P		63 [42–120]	161 [146–176]	I–II	Enflurane with N₂O	45 [20–249]		Gynecologic surgery
				T		62 [41–101]	165 [148–181]			37 [20–300]		
Argiriadou, 2002 [31]	31 (87)	NR	21/66	P		68.5 ± 8.3	NR	I–II	Sevoflurane	69 ± 22		Laparoscopic cholecystectomy
				T		73.4 ± 13				75 ± 23		
				O		71.5 ± 14.6				67.4 ± 15		
Capouet, 1996 [32]	97/95/96 (385)	18–75	0/385	P		66 ± 13	163 ± 7	I–II	Isoflurane with N₂O	74 [17–229]		Gynecologic surgery (one breast surgery)
				T 0.5 mg		63 ± 11	164 ± 6			76 [21–177]		
				T 2 mg		66 ± 15	164 ± 6			80 [24–281]		
				T 5 mg		65 ± 12	163 ± 7			78 [15–207]		
Chan, 1998 [33]	49/49 (148)	18–75	0/148	P		55 [40–67]	158 [148–165]	I–II	Isoflurane with N₂O	156 [102–270]		Breast surgery
				T 2 mg		55 [35–73]	157 [146–165]			162 [72–258]		
				T 5 mg		53 [36–77]	157 [143–169]			156 [90–216]		
Contreras-Dominguez, 2008 [34]	25 (150)	18–65	76/74	NR			NR	I–II	Isoflurane with N₂O	P	47.2 ± 5.4 ^a^	Appendectomy
										D	43.7 ± 6.1 ^a^	
										M	56.8 ± 6.5 ^a^	
										T	59.2 ± 2.6 ^a^	
										G	48.7 ± 7.2 ^a^	
										Dex	55.3 ± 3.8 ^a^	
Eberhart, 2006 [35]	80 (310)	NR	88/222	P		75 (61–82)	168 (164–175)	I–III	Desflurane	105 (80–145)		Gynecological and ENT surgery
				T		71 (62–81)	170 (162–174)			115 (90–150)		
				Dex		75 (63–84)	170 (164–176)			105 (75–150)		
				T + Dex		70 (64–84)	168 (164–176)			100 (75–150)		
Ekinci, 2011 [36]	20 (100)	20–72	0/100	P		66.75 ± 7.44	NR	I–II	Isoflurane with N₂O	97.508 ± 22.03 ^a^		
				D		66.75 ± 9.32				81.758 ± 20.34 ^a^		
				M		66.85 ± 8.68				90.008 ± 28.38 ^a^		
				T		67.75 ± 8.21				93.008 ± 22.09		
				O		66.05 ± 12.51				89.508 ± 21.51		
Jokela, 1999 [37]	60 (120)	18-	0/120	NR			NR	I–III	Sevoflurane	T	112 [60–213]	Laparoscopic cholecystectomy (five converted to laparotomy)
										D	106 [63–202]	
Jokela, 2002 [38]	60 (179)	NR	0/179	NR			NR	I–III	Sevoflurane	T	108 ± 38 ^a^	Thyroid or parathyroid surgery
										O	123 ± 55 ^a^	
										M	111 ± 47 ^a^	
Koivuranta, 1999 [39]	43 (88)	18-	0/88	NR			NR	I–III	Sevoflurane	P +D	347.0 ± 182.5	Supratentorial craniotomy
										T +D	288.0 ± 86.4	
Madenoglu, 2003 [40]	30 (60)	18–76	29/31	P		74.8 ± 10.4	NR	I–III	Isoflurane with N₂O	P	347.0 ± 182.5	Supratentorial craniotomy
				T		70.2 ± 10.9				T	288.0 ± 86.4	
Mei, 2014 [41]	128 (295)	18–35	0/295	P	PPF	52.8 ± 7.2	160.0 ± 4.8	I–II	Propofol or Sevoflurane	73 (55–102) ^a^		Gynecologic surgery
					Sev	51.8 ± 6.9	159.6 ± 4.3			81 (60–99) ^a^		
				T	PPF	53.5 ± 7.3	160.6 ± 4.3			80 (61–111) ^a^		
					Sev	53.2 ± 8.4	159.2 ± 4.1			78 (57–99) ^a^		
Metaxari, 2011 [42]	52 (203)	20–65	0/203	P		65 ± 8	162 ± 5	I–II	Sevoflurane	113 ± 29 ^a^		Thyroid surgery
				T		64 ± 8	163 ± 5			125 ± 34 ^a^		
				G		68 ± 8	162 ± 5			115 ± 35 ^a^		
				O		66 ± 8	163 ± 5			120 ± 30 ^a^		
Muhammad, 2000 [43]	16 (50)	22–71	NR	NR			NR	I–II	Halothane with N₂O	NR		Mini-laparotomy cholecystectomy
Naguib, 1996 [44]	25 (132)	21–68	24/108	P		69.9 ± 13.5	NR	I–II	Isoflurane with N₂O	129.5 ± 34.5		Laparoscopic cholecystectomy
				T		76.8 ± 13.4				113.4 ± 29.9		
				O		72.2 ± 12.9				128.3 ± 42		
				G		72.4 ± 12.2				112.4 ± 36.5		
				M		71.3 ± 12.3				126.5 ± 36.6		
Papadimitriou, 2001 [45]	63 (120)	27–43	0/120	M		65.5 ± 5.7	NR	I–II	Sevoflurane with N₂O	74.0 ± 7.5		Laparoscopic gynecological surgery
				M + T		65.9 ± 6.0				75.2 ± 7.8		
Purhonen, 1997 [46]	48 (146)	NR	0/146	P		71 ± 2	162 ± 1	I–III	Isoflurane with N₂O	145 ± 7		Elective gynecologic incontinence surgery
				T		73 ± 2	163 ± 1			149 ± 9		
				D		70 ± 2	160 ± 1			144 ± 8		
Quan, 2007 [47]	118 (356)	18–75	NR	NR			NR	I–II	NR	NR		NR
Scholz, 1998 [48]	296 (842)	18–75	220/622	P		70.7 [42–114]	167.5 [150–192]	I–III	Isoflurane with N₂O	NR		Abdominal (504), ENT (201), Eye (14), Breast (121), Others (2)
				T		71.4 [42–120]	168.8 [147–198]					
				O		71.0 [46–110]	168.6 [145–194]					
Tsui, 1999 [49]	37 (121)	-65	0/121	P		55.6 ± 12	NR	I–III	Isoflurane with N₂O	87 ± 60 ^a^		Gynecological laparotomy surgery
				T		59.5 ± 10.3				89 ± 47 ^a^		
				O		55.9 ± 11.6				100 ± 60 ^a^		
Wang, 2002 [50]	38 (115)	NR	43/72	P		58 ± 5	NR	I–II	Isoflurane	82 ± 12		Elective laparoscopic cholecystectomy
				T		58 ± 6				86 ± 12		
				Dex		56 ± 4				80 ± 14		
Wang, 2002 [51]	38 (116)	35–55		P		64 ± 8	NR	I–II	Sevoflurane or Desflurane	170 ± 24		Tympanomastoid surgery
				T		63 ± 7				169 ± 26		
				Dex		65 ± 7				178 ± 25		
Yang, 2015 [52]	53 (153)	18–60	0/154	NR			NR	I–II	Sevoflurane	Dex	100 (23–219)	Elective gynecological laparoscopic surgery
										Dex + T	109 (50–251)	
Yi, 2022 [53]	60 (180)	18–65	0/180	Dex		T61.80 ± 1.03	161 ± 1	I–II	Remimazolam	24.50 [17.50–29.50]		Gynecological day surgery
				Dex + T		62.05 ± 1.30	162 ± 1			24.50 [16.00–36.75]		
				Dex + D		61.80 ± 1.03	162 ± 1			23.00 [20.00–30.00]		
Zhou, 2012 [54]	50 (150)	18-	36/114	NR			NR	NR	Sevoflurane	Dex	74.0 ± 27.3 ^a^	Thyroid surgery
										Dex + T	78.7 ± 32.2 ^a^	
										T	81.7 ± 32.6 ^a^	
Papadima, 2013 [55]	40 (127)	18–75	27/100	P		68 ± 9.9	NR	NR	Sevoflurane	127.1 ± 14.4		Total thyroidectomy
				T		68 ± 8.9				128.4 ± 12.4		
				G		68 ± 9.4				113.8 ± 18.9		
Jakobsson, 1999 [56]	34 (68)	18–50	0/68	P		64 ± 10	NR	I–II	Isoflurane with N₂O	61 ± 19		Laparoscopic gynecological surgery
				T		65 ± 10				57 ± 29		
Zomers, 1993 [57]	35 (69)	18–75	0/69	P		68	167	NR	Isoflurane with N₂O	NR		Gynecological surgery
				T		66	165					

No.; number, yrs; years, M; male, F; female, kg; kilogram, cm; centimeter; ASA-PS; American Society of Anesthesiologists Physical Status, NR; not reported, min; minutes, P; placebo, T; tropisetron, mg; milligram, M;metoclopramide, D; droperidol, Dex; dexamethasone, G; granisetron, O; ondansetron, PPF; propofol, and Sev; sevoflurane. ^a^: duration of surgery. Data are presented as absolute number, [range], mean ± standard deviation, median (Q_1_–Q_3_).

**Table 2:** Risk of bias.

Author, Year	Bias Arising from the Randomization Process	Bias Due to Deviations from Intended Intervention	Bias Due to Missing Outcome Data	Bias in Measurement of the Outcome	Bias in Selection of the Reported Results	Overall Bias
Akin, 2006 [28]	Some concern	Low risk	Low risk	Low risk	Low risk	Some concern
Ali-Melkkilä, 1996 [29]	Some concern	Low risk	Low risk	Some concern	Low risk	High risk
Alon, 1996 [30]	Some concern	Low risk	Low risk	Low risk	Low risk	Some concern
Argiriadou, 2002 [31]	Low risk	Low risk	Low risk	Low risk	Low risk	Low risk
Capouet, 1996 [32]	Low risk	Low risk	Low risk	Low risk	Low risk	Low risk
Chan, 1998 [33]	Low risk	Low risk	Low risk	Low risk	Low risk	Low risk
Contreras-Dominguez, 2008 [34]	Some concern	Low risk	Low risk	Low risk	Low risk	Some concern
Eberhart, 2006 [35]	Low risk	Low risk	Low risk	Low risk	Low risk	Low risk
Ekinci, 2011 [36]	Low risk	Low risk	Low risk	Some concern	Low risk	Some concern
Jokela, 1999 [37]	Low risk	Low risk	Low risk	Low risk	Low risk	Low risk
Jokela, 2002 [38]	Low risk	Low risk	Low risk	Low risk	Low risk	Low risk
Koivuranta, 1999 [39]	Some concern	Low risk	Low risk	Low risk	Low risk	Some concern
Madenoglu,2003 [40]	Low risk	Low risk	Low risk	Low risk	Low risk	Low risk
Mei, 2014 [41]	Low risk	Low risk	Low risk	Low risk	Low risk	Low risk
Metaxari, 2011 [42]	Some concern	Low risk	Low risk	Low risk	Low risk	Some concern
Muhammad, 2000 [43]	Some concern	Low risk	Low risk	Low risk	Low risk	Some concern
Naguib, 1996 [44]	Low risk	Low risk	Low risk	Low risk	Low risk	Low risk
Papadimitriou, 2001 [45]	Some concern	Low risk	Low risk	Low risk	Low risk	Some concern
Purhonen, 1997 [46]	Some concern	Low risk	Low risk	Low risk	Low risk	Some concern
Quan, 2007 [47]	Some concern	Low risk	Low risk	Low risk	Low risk	Some concern
Scholz, 1998 [48]	Some concern	Low risk	Low risk	Low risk	Low risk	Some concern
Tsui, 1999 [49]	Some concern	Low risk	Low risk	Low risk	Low risk	Some concern
Wang, 2002 [50]	Low risk	Low risk	Low risk	Low risk	Low risk	Low risk
Wang, 2002 [51]	Low risk	Low risk	Low risk	Low risk	Low risk	Low risk
Yang, 2015 [52]	Low risk	Low risk	Low risk	Low risk	Low risk	Low risk
Yi, 2022 [53]	Low risk	Low risk	Low risk	Low risk	Low risk	Low risk
Zhou, 2012 [54]	Low risk	Low risk	Low risk	Low risk	Low risk	Low risk
Papadima, 2013 [55]	Low risk	Low risk	Low risk	Low risk	Low risk	Low risk
Jakobsson, 1999 [56]	Some concern	Low risk	Low risk	Some concern	Low risk	High risk
Zomers, 1993 [57]	Some concern	Low risk	Low risk	Some concern	Low risk	High risk

**Table 3:** Summary of the meta-analysis.

		No. of Studies	No. of Patients	Conventional Meta-Analysis				Trial Sequential Analysis			NNT
				RR with 95% CI	Heterogeneity (I^2^; P_chi_^2^; τ; 95% PI)	Sensitivity Analysis	Conventional Test Boundary	Trial Sequential Monitoring Boundary	Futility Boundary	RIS	
PON	vs. control	12	1955	Significant (RR: 0.718; 95% CI 0.652–0.790)	I^2^ = 0.0; P_chi_^2^ = 0.612; τ = 0.0	NC	Exceeded	Exceeded	Not Exceeded	Exceeded RIS (1754 of 992)	Significant (NNTB: 8; 95% CI NNTB 6 to NNTB 12)
	vs. control1	15	2312	Significant (RR: 0.814; 95% CI 0.683–0.971)	I^2^ = 65.38; P_chi_^2^ < 0.001; τ = 0.070, 95% PI 0.625–1.060	C ^a^	Exceeded	Exceeded	Not Exceeded	Exceeded RIS (2312 of 801)	Significant (NNTB: 7; 95% CI NNTB 6 to NNTB 10)
	vs. metoclopramide	1	80	Not significant (RR: 0.786; 95% CI 0.407–1.516)		NC					Not significant (NNTB: 13; 95% CI NNTH 8 to ∞ to NNTB 4)
	vs. ondansetron	4	984	Not significant (RR: 0.981; 95% CI 0.828–1.163)	I^2^ = 0.00; P_chi_^2^ = 0.684; τ = 0.00	NC	Not Exceeded	Not Exceeded	Exceeded	63.1% (984 of 1559 patients)	Not significant (NNTH: 552; 95% CI NNTH 17 to ∞ to NNTB 18)
	vs. ondansetron1	5	1072	Not significant (RR: 1.016; 95% CI 0.865–1.194)	I^2^ = 0.00; P_chi_^2^ = 0.548; τ = 0.00	NC	Not Exceeded	Not Exceeded	Exceeded	69.7% (1072 of 1538 patients)	Not significant (NNTH: 84; 95% CI NNTH 15 to ∞ to NNTB 23)
	vs. dexamethasone	4	413	Not significant (RR: 0.994; 95% CI 0.718–1.375)	I^2^ = 16.00; P_chi_^2^ = 0.312; τ = 0.137; 95% PI 0.699–2.273	NC	Not Exceeded	Not Exceeded	Not Exceeded	20.2% (413 of 2023 patients)	Not significant (NNTH: 273; 95% CI NNTH 11 to ∞ to NNTB 12)
	vs. droperidol	1	120	Not significant (RR: 0.892; 95% CI 0.658–1.208)		NC					Not significant (NNTB: 15; 95% CI NNTH 9 to ∞ to NNTB 4)
	vs. granisetron	2	338	Not significant (RR: 1.296; 95% CI 0.816–2.057)	I^2^ = 0.00; P_chi_^2^ = 0.472; τ = 0.00	NC	Not Exceeded	Not Exceeded	Not Exceeded	8.2% (338 of 4106 patients)	Not significant (NNTH: 22; 95% CI NNTH 8 to ∞ to NNTB 29)
POV	vs. control	14	2048	Significant (RR: 0.587; 95% CI 0.455–0.757)	I^2^ = 63.32; P_chi_^2^ = 0.001; τ = 0.346, 95% PI 0.415–0.829	NC	Exceeded	Exceeded	Not Exceeded	38.5% (2048 of 5314 patients)	Significant (NNTB: 11; 95% CI NNTB 7 to NNTB 19)
	vs. control1	18	2487	Significant (RR: 0.592; 95% CI 0.479–0.732)	I^2^ = 53.41; P_chi_^2^ = 0.004; τ = 0.300, 95% PI 0.439–0.799	NC	Exceeded	Exceeded	Not Exceeded	63.0% (2487 of 3947 patients)	Significant (NNTB: 10 95% CI NNTB 7 to NNTB 15)
	vs. metoclopramide	1	80	Not significant (RR: 1.833; 95% CI 0.751–4.478)		NC					Not significant (NNTH: 8; 95% CI NNTH 3 to ∞ to NNTB 19)
	vs. ondansetron	6	1120	Not significant (RR: 0.940; 95% CI 0.649–1.361)	I^2^ = 25.32; P_chi_^2^ = 0.244; τ = 0.233, 95% PI 0.611–1.636	NC	Not Exceeded	Not Exceeded	Not Exceeded	19.8% (1120 of 5659 patients)	Not significant (NNTB: 2405; 95% CI NNTH 17 to ∞ to NNTB 16)
	vs. ondansetron1	7	1208	Not significant (RR: 0.989; 95% CI 0.742–1.319)	I^2^ = 10.40; P_chi_^2^ = 0.350; τ = 0.138, 95% PI 0.136–7.166	NC	Not Exceeded	Not Exceeded	Not Exceeded	24.4% (1208 of 4955 patients)	Not significant (NNTH: 161; 95% CI NNTH 20 to ∞ to NNTB 26)
	vs. dexamethasone	3	332	Not significant (RR: 1.286; 95% CI 0.803–2.058)	I^2^ = 16.30; P_chi_^2^ = 0.303; τ = 0.173, 95% PI 0.776–2.13	NC	Not Exceeded	Not Exceeded	Not Exceeded	8.1% (332 of 4120 patients)	Not significant (NNTH: 18; 95% CI NNTH 7 to ∞ to NNTB 30)
	vs. droperidol	2	217	Significant (RR: 0.400; 95% CI 0.260–0.614)	I^2^ = 0.00; P_chi_^2^ = 0.864; τ = 0.00	NC	Exceeded	Met	Not Exceeded	26.5% (217 of 818 patients)	Significant (NNTB: 4; 95% CI NNTB 3 to NNTB 9)
	vs. granisetron	2	338	Not significant (RR: 1.594; 95% CI 0.757–3.358)	I^2^ = 0.00; P_chi_^2^ = 0.910; τ = 0.00	NC	Not Exceeded	Not Exceeded	Not Exceeded	6.8% (338 of 4955 patients)	Not significant (NNTH: 29; 95% CI NNTH 11 to ∞ to NNTB 45)
	vs. granisetron1	3	423	Not significant (RR: 1.404; 95% CI 0.830–2.376)	I^2^ = 0.00; P_chi_^2^ = 0.0.889; τ = 0.00	NC	Not Exceeded	not Exceeded	Not Exceeded	6.1% (423 of 6880 patients)	Not significant (NNTH: 28; 95% CI NNTH 11 to ∞ to NNTB 40)
PONV	vs. control	11	953	Significant (RR: 0.655; 95% CI 0.532–0.806)	I^2^ = 49.09; P_chi_^2^ = 0.039; τ = 0.218, 95% PI 0.440–0.975;	NC	Exceeded	Exceeded	Not Exceeded	40.0% (953 of 2380 patients)	Significant (NNTB: 5; 95% CI NNTB 4 to NNTB 7)
	vs. control1	15	1428	Significant (RR: 0.614; 95% CI 0.504–0.749)	I^2^ = 59.21; P_chi_^2^ = 0.002; τ = 0.280, 95% PI 0.375–1.004	NC	Exceeded	Exceeded	Not Exceeded	69.7% (1428 of 2048 patients)	Significant (NNTB: 5 95% CI NNTB 4 to NNTB 7)
	vs. metoclopramide	5	290	Significant (RR: 0.770; 95% CI 0.624–0.950)	I^2^ = 0.00; P_chi_^2^ = 0.419; τ = 0.00; 95% PI 0.440–0.975	C ^b^	Exceeded	Not Exceeded	Not Exceeded	46.9% (290 of 618 patients)	Significant (NNTB: 7; 95% CI NNTB 4 to NNTB 32)
	vs. ondansetron	3	214	Not significant (RR: 1.063; 95% CI 0.727–1.553)	I^2^ = 38.38; τ = 0.209; P_chi_^2^ = 0.047; 95% PI 0.564–2.001	NC	Not Exceeded	Not Exceeded	Not Exceeded	11.5% (214 of 1876 patients)patients)	Not significant (NNTH: 93; 95% CI NNTH 7 to ∞ to NNTB 8)
	vs. dexamethasone	3	282	Not significant (RR: 1.129; 95% CI 0.552–2.306)	I^2^ = 70.56; τ = 0.514, P_chi_^2^ = 0.033; 95% PI 0.252–5.061	NC	Not Exceeded	Not Exceeded	Not Exceeded	6.1% (282 of 7432 patients)	Not significant (NNTH: 21; 95% CI NNTH 6 to ∞ to NNTB 15)
	vs. droperidol	3	187	Not significant (RR: 0.970; 95% CI 0.787–1.196)	I^2^ = 0.0; P_chi_^2^ = 0.586; τ = 0.0	NC	Not Exceeded	Not Exceeded	Not Exceeded	25.9% (187 of 721 patients)	Not significant (NNTB: 194; 95% CI NNTH 7 to ∞ to NNTB 7)
	vs. droperidol1	4	285	Not significant (RR: 0.981; 95% CI 0.812–1.185)	I^2^ = 0.0; P_chi_^2^ = 0.771; τ = 0.0	NC	Not Exceeded	Not Exceeded	Not Exceeded	36.1% (285 of 789 patients),	Not significant (NNTB: 294; 95% CI NNTH 9 to ∞ to NNTB 8)
	vs. granisetron	2	100	Not significant (RR: 1.073; 95% CI 0.637–1.806)	I^2^ = 0.0; P_chi_^2^ = 0.922; τ = 0.0	NC	Not Exceeded	Not Exceeded	Not Exceeded	12.7% (100 of 1719 patients)	Not significant (NNTH: 50; 95% CI NNTH 5 to ∞ to NNTB 6)
Use of RA	vs. control	16	2078	Significant (RR: 0.622; 95% CI 0.552–0.700)	I^2^ = 0.00; P_chi_^2^ = 0.492; τ = 0.00	NC	Exceeded	Exceeded	Not Exceeded	exceeds RIS (2078 of 1027 patients)	Significant (NNTB: 6; 95% CI NNTB 5 to NNTB 9)
	vs. control1	21	2738	Significant (0.620; 95% CI 0.556–0.692)	I^2^ = 0.00; P_chi_^2^ = 0.456; τ = 0.00	NC	Exceeded	Exceeded	Not Exceeded	60.8% (2738 of 4506)	Significant (NNTB: 10 95% CI NNTB 7 to NNTB 16)
	vs. metoclopramide	5	320	Significant (RR: 0.707; 95% CI 0.561–0.892)	I^2^ = 0.0; P_chi_^2^ = 0.618; τ = 0.0	NC	Exceeded	Not Exceeded	Not Exceeded	41.6% (320 of 769 patients),	Significant (NNTB: 7; 95% CI NNTH 4 to NNTB 24)
	vs. ondansetron	6	917	Not significant (RR: 0.968; 95% CI 0.759–1.235)	I^2^ = 18.02; τ = 0.130; P_chi_^2^ = 0.295; 95% PI 0.744–1.256	NC	Not Exceeded	Not Exceeded	Not Exceeded	31.4% (917 of 2925 patients)	Not significant (NNTB: 123; 95% CI NNTH 20 to ∞ to NNTB 15)
	vs. ondansetron1	7	1005	Not significant (RR: 1.000; 95% CI 0.806–1.242)	I^2^ = 13.46; τ = 0.130; P_chi_^2^ = 0.327; 95% PI 0.799–1.352	NC	Not Exceeded	Not Exceeded	Not Exceeded	34.4% (1005 of 2541 patients)	Not significant (NNTH: 84; 95% CI NNTH 15 to ∞ to NNTB 23)
	vs. dexamethasone	4	408	Not significant (RR: 0.988; 95% CI 0.594–1.645)	I^2^ = 48.78; P_chi_^2^ = 0.434; τ = 0.358; 95% PI 0.426–2.293	NC	Not Exceeded	Not Exceeded	Not Exceeded	16.1% (408 of 4644 patients)	Not significant (NNTB: 51; 95% CI NNTH 16 to ∞ to NNTB 10)
	vs. droperidol	3	257	Not significant (RR: 0.864; 95% CI 0.654–1.142)	I^2^ = 0.00; P_chi_^2^ = 0.809; τ = 0.00	NC	Not Exceeded	Not Exceeded	Not Exceeded	28.2% (257 of 910 patients)	Not significant (NNTB: 17; 95% CI NNTH 16 to ∞ to NNTB 6)
	vs. granisetron	1	50	Not significant (RR: 1.296; 95% CI 0.816–2.057)		NC					Not significant (NNTH: 22; 95% CI NNTH 8 to ∞ to NNTB 29)
	vs. granisetron1	2	135	Not significant (RR: 1.250; 95% CI 0.871–1.795)	I^2^ = 0.00; P_chi_^2^ = 0.505; τ = 0.00	NC	Not Exceeded	Not Exceeded	Not Exceeded	14.8% (135 of 1071 patients)	Not significant (NNTH: 25; 95% CI NNTH 3 to ∞ to NNTB 4)
CR	vs. control	9	823	Significant (RR: 1.517; 95% CI 1.222–1.885)	I^2^ = 44.14; P_chi_^2^ = 0.074; τ = 0.215, 95% PI 1.018–2.260	NC	Exceeded	Not Exceeded	Not Exceeded	29.0% (823 of 2838 patients)	Significant (NNTB: 1; 95% CI NNTB 1 to NNTB 1)
	vs. control1	12	1175	Significant (1.442; 95% CI 1.250–1.663)	I^2^ = 30.00; P_chi_^2^ = 0.152; τ = 0.133; 95% PI 1.133–1.834	NC	Exceeded	Exceeded	Not Exceeded	63.0% (1175 of 1864 patients)	Significant (NNTB: 5 95% CI NNTB 4 to NNTB 8)
	vs. metoclopramide	3	239	Not significant (RR: 1.180; 95% CI 0.933–1.493)	I^2^ = 0.00; P_chi_^2^ = 0.377; τ = 0.00	NC	Not Exceeded	Not Exceeded	Not Exceeded	21.9% (239 of 1093 patients)	Not significant (NNTB: 8; 95% CI NNTH 85 to ∞ to NNTB 4)
	vs. ondansetron	4	452	RR: 0.928; 95% CI 0.726–1.185	I^2^ = 41.88; P_chi_^2^ = 0.160; τ = 0.162; 95% PI 0.635–1.356	NC	Not Exceeded	Not Exceeded	Not Exceeded	29.1% (452 of 1551 patients)	Not significant (NNTB: 8; 95% CI NNTH 85 to ∞ to NNTB 4)
	vs. dexamethasone	4	408	RR: 0.939 CI 0.747–1.181	I^2^ = 56.21; P_chi_^2^ = 0.077; τ = 0.174; 95% PI 0.625–1.412	NC	Not Exceeded	Not Exceeded	Not Exceeded	42.0% (408 of 971 patients)	Not significant (NNTB: 8; 95% CI NNTH 85 to ∞ to NNTB 4)
	vs. droperidol	3	257	RR: 1.142;95% CI 0.836–1.558	I^2^ = 0.00; P_chi_^2^ = 0.982; τ = 0.00	NC	Not Exceeded	Not Exceeded	Not Exceeded	17.9% (257 of 1436 patients	Not significant (NNTB: 20; 95% CI NNTH 15 to ∞ to NNTB 6)
	vs. droperidol1	4	355	RR: 1.075;95% CI 0.850–1.359	I^2^ = 0.00; P_chi_^2^ = 0.946; τ = 0.00	NC	Not Exceeded	Not Exceeded	Not Exceeded	24.7% (355 of 1140 patients)	Not significant (NNTB: 26; 95% CI NNTH 16 to ∞ to NNTB 7)
	vs. granisetron	2	286	RR: 0.934;95% CI 0.798–1.093	I^2^ = 0.00; P_chi_^2^ = 0.742; τ = 0.00	NC	Not Exceeded	Not Exceeded	Not Exceeded	39.3% (286 of 728 patients)	Not significant (NNTH: 20; 95% CI NNTH 6 to ∞ to NNTB 17)

No; number, RR; relative risk, CI; confidence interval, PI; predictive interval, RIS; required information size, NNT; number needed to treat, PON; post-operative nausea; NC; not change significance, C; change significance, NNTB; number needed to treat to benefit, control1; compared with control when including studies comparing other anti-emetic agents to tropisetron combined with other anti-emetic agents, NNTH; number needed to treat to harm, ondansetron1; compared with ondansetron when including studies comparing tropisetron combined with other anti-emetic agents to ondansetron combined with other anti-emetic agents, POV; post-operative vomiting, granisetron1; compared with granisetron when including studies comparing tropisetron combined with other anti-emetic agents to granisetron combined with other anti-emetic agents, PONV; post-operative nausea and vomiting, droperidol1; compared with droperidol when including studies comparing tropisetron combined with other anti-emetics to droperidol combined with other anti-emetics, RA; rescue anti-emetics, CR; complete response. ^a^; the exclusion of the studies conducted by Capouet et al. [32], Chan et al. [33], and Ebehart et al. [35] resulted in a statistically significant difference between the tropisetron and control1 group, ^b^; the exclusion of the studies conducted by Naguib et al. [44] and Jokela et al. [38] resulted in a statistically significant difference between tropisetron and metoclopramide.

**Table 4:** GRADE evidence quality for each outcome.

Outcomes		Number of Studies	Quality Assessment					Quality
			ROB	Inconsistency	Indirectness	Imprecision	Publication Bias	
PON	vs. control	12	Not serious	Not serious	Not serious	Not serious	Not serious	⨁⨁⨁⨁ High
	vs. control1	15	Not serious	Serious ^a^	Not serious	Not serious	Not serious	⨁⨁⨁◯ Moderate
	vs. metoclopramide	1	Not serious	Not serious	Not serious	Serious	NA	⨁⨁⨁◯ Moderate
	vs. ondansetron	4	Not serious	Not serious	Not serious	Serious	NA	⨁⨁⨁◯ Moderate
	vs. ondansetron1	5	Not serious	Not serious	Not serious	Serious	NA	⨁⨁⨁◯ Moderate
	vs. dexamethasone	4	Not serious	Not serious	Not serious	Serious	NA	⨁⨁⨁◯ Moderate
	vs. droperidol	1	Not serious	Not serious	Not serious	Serious	NA	⨁⨁⨁◯ Moderate
	vs. granisetron	2	Not serious	Not serious	Not serious	Serious	NA	⨁⨁⨁◯ Moderate
POV	vs. control	14	Not serious	Serious ^a^	Not serious	Not serious	Not serious	⨁⨁⨁◯ Moderate
	vs. control1	18	Not serious	Serious ^a^	Not serious	Not serious	Not serious	⨁⨁⨁◯ Moderate
	vs. metoclopramide	1	Not serious	Not serious	Not serious	Serious	NA	⨁⨁⨁◯ Moderate
	vs. ondansetron	6	Not serious	Not serious	Not serious	Serious	NA	⨁⨁⨁◯ Moderate
	vs. ondansetron1	7	Not serious	Not serious	Not serious	Serious	NA	⨁⨁⨁◯ Moderate
	vs. dexamethasone	3	Not serious	Not serious	Not serious	Serious	NA	⨁⨁⨁◯ Moderate
	vs. droperidol	2	Not serious	Not serious	Not serious	Serious	NA	⨁⨁⨁◯ Moderate
	vs. granisetron	2	Not serious	Not serious	Not serious	Serious	Not serious	⨁⨁⨁◯ Moderate
	vs. granisetron1	3	Not serious	Not serious	Not serious	Serious	NA	⨁⨁⨁◯ Moderate
PONV	vs. control	11	Not serious	Not serious	Not serious	Not serious	Not serious ^b^	⨁⨁⨁⨁ High
	vs. control1	15	Not serious	Serious ^a^	Not serious	Not serious	Not serious ^b^	⨁⨁⨁◯ Moderate
	vs. metoclopramide	5	Not serious	Not serious	Not serious	Not serious	NA	⨁⨁⨁⨁ High
	vs. ondansetron	3	Not serious	Not serious	Not serious	Serious	NA	⨁⨁⨁◯ Moderate
	vs. dexamethasone	3	Not serious	Serious ^a^	Not serious	Serious	NA	⨁⨁◯◯ Low
	vs. droperidol	3	Not serious	Not serious	Not serious	Serious	NA	⨁⨁⨁◯ Moderate
	vs. droperidol1	4	Not serious	Not serious	Not serious	Serious	NA	⨁⨁⨁◯ Moderate
	vs. granisetron	2	Not serious	Not serious	Not serious	Serious	NA	⨁⨁⨁◯ Moderate
Use of RA	vs. control	16	Not serious	Not serious	Not serious	Not serious	Not serious ^b^	⨁⨁⨁⨁ High
	vs. control1	21	Not serious	Not serious	Not serious	Not serious	Not serious ^b^	⨁⨁⨁⨁ High
	vs. metoclopramide	5	Not serious	Not serious	Not serious	Serious	NA	⨁⨁⨁◯ Moderate
	vs. ondansetron	6	Not serious	Not serious	Not serious	Serious	NA	⨁⨁⨁◯ Moderate
	vs. ondansetron1	7	Not serious	Not serious	Not serious	Serious	NA	⨁⨁⨁◯ Moderate
	vs. dexamethasone	4	Not serious	Not serious	Not serious	Serious	NA	⨁⨁⨁◯ Moderate
	vs. droperidol	3	Not serious	Not serious	Not serious	Serious	NA	⨁⨁⨁◯ Moderate
	vs. granisetron	1	Not serious	Not serious	Not serious	Serious	NA	⨁⨁⨁◯ Moderate
	vs. granisetron1	2	Not serious	Not serious	Not serious	Serious	NA	⨁⨁⨁◯ Moderate
CR	vs. control	9	Not serious	Not serious	Not serious	Not serious	NA	⨁⨁⨁⨁ High
	vs. control1	12	Not serious	Not serious	Not serious	Not serious	Not serious	⨁⨁⨁⨁ High
	vs. metoclopramide	3	Not serious	Not serious	Not serious	Serious	NA	⨁⨁⨁◯ Moderate
	vs. ondansetron	4	Not serious	Not serious	Not serious	Serious	NA	⨁⨁⨁◯ Moderate
	vs. dexamethasone	4	Not serious	Serious ^a^	Not serious	Serious	NA	⨁⨁◯◯ Low
	vs. droperidol	3	Not serious	Not serious	Not serious	Serious	NA	⨁⨁⨁◯ Moderate
	vs. droperidol1	4	Not serious	Not serious	Not serious	Serious	NA	⨁⨁⨁◯ Moderate
	vs. granisetron	2	Not serious	Not serious	Not serious	Serious	NA	⨁⨁⨁◯ Moderate

ROB; Risk of bias, post-operative nausea; control1; compared with control when including studies comparing other anti-emetic agents to tropisetron combined with other anti-emetic agents, ondansetron1; compared with ondansetron when including studies comparing tropisetron combined with other anti-emetic agents to ondansetron combined with other anti-emetic agents, POV; post-operative vomiting, granisetron1; compared with granisetron when including studies comparing tropisetron combined with other anti-emetic agents to granisetron combined with other anti-emetic agents, PONV; post-operative nausea and vomiting, droperidol1; compared with droperidol when including studies comparing tropisetron combined with other anti-emetics to droperidol combined with other anti-emetics, RA; rescue anti-emetics, CR; complete response. ^a^: considered serious as the I^2^ value was >50% or P_chi_^2^ value was <0.010; ^b^: regarded as not serious as the trim-and-fill analysis did not reveal any statistically significant changes.

## Data Availability

The datasets used and analyzed during the current study are available from the corresponding author upon reasonable request.

## Supplementary Materials

The following supporting information can be downloaded at: https://www.mdpi.com/article/10.3390/jpm14080797/s1, Figure S1: Forest plot for post-operative nausea comparing tropisetron and control including studies comparing dexamethasone and tropisetron combined with dexamethasone; Figure S2: Trial sequential analysis plot for post-operative nausea comparing tropisetron and control including studies comparing dexamethasone and tropisetron combined with dexamethasone; Figure S3: Forest plot for post-operative nausea comparing tropisetron and metoclopramide; Figure S4: Forest plot for post-operative nausea comparing tropisetron and ondansetron; Figure S5: Forest plot for post-operative nausea comparing tropisetron and ondansetron including studies comparing tropisetron combined with droperidol and ondansetron combined with droperidol; Figure S6: Forest plot for post-operative nausea comparing tropisetron and dexamethasone; Figure S7: Forest plot for post-operative nausea comparing tropisetron and droperidol; Figure S8: Forest plot for post-operative nausea comparing tropisetron and granisetron; Figure S9: Trial sequential analysis plot for post-operative nausea comparing tropisetron and ondansetron; Figure S10: Trial sequential analysis plot for post-operative nausea comparing tropisetron and ondansetron when including study comparing tropisetron combined with droperidol and tropisetron combined with ondansetron; Figure S11: Trial sequential analysis plot for post-operative nausea comparing tropisetron and dexamethasone; Figure S12: Trial sequential analysis plot for post-operative nausea comparing tropisetron and granisetron; Figure S13: Forest plot for post-operative vomiting comparing tropisetron and control groups including studies comparing droperidol and tropisetron combined with droperidol and comparing dexamethasone and tropisetron combined with dexamethasone; Figure S14: Trial sequential analysis plot for post-operative vomiting comparing tropisetron and control including studies comparing droperidol and tropisetron combined with droperidol and comparing dexamethasone and tropisetron combined with dexamethasone; Figure S15: Forest plot for post-operative vomiting comparing tropisetron and droperidol; Figure S16: Forest plot for post-operative vomiting comparing tropisetron and metoclopramide; Figure S17: Forest plot for post-operative vomiting comparing tropisetron and ondansetron; Figure S18: Forest plot for post-operative vomiting comparing tropisetron and ondansetron including studies comparing tropisetron combined with droperidol and ondansetron combined with droperidol; Figure S19: Forest plot for post-operative vomiting comparing tropisetron and dexamethasone; Figure S20: Forest plot for post-operative vomiting comparing tropisetron and granisetron; Figure S21: Forest plot for post-operative vomiting comparing tropisetron and granisetron including studies comparing tropisetron combined with droperidol and granisetron combined with droperidol; Figure S22: Trial sequential analysis plot for post-operative vomiting comparing tropisetron and ondansetron; Figure S23: Trial sequential analysis plot for post-operative vomiting comparing tropisetron and ondansetron when including studies comparing tropisetron combined with droperidol and ondansetron combined with droperidol; Figure S24: Trial sequential analysis plot for post-operative vomiting comparing tropisetron and dexamethasone; Figure S25: Trial sequential analysis plot for post-operative vomiting comparing tropisetron and droperidol, Figure S26: Trial sequential analysis plot for post-operative vomiting comparing tropisetron and granisetron; Figure S27: Trial sequential analysis plot for post-operative vomiting comparing tropisetron and graniseetron when including studies comparing tropisetron combined with droperidol and granisetron combined with droperidol; Figure S28: Forest plot for post-operative nausea and vomiting comparing tropisetron and control including studies comparing dexamethasone and tropisetron combined with dexamethasone and comparing metoclopramide and tropisetron combined with metoclopramide; Figure S29: Trial sequential analysis plot for post-operative nausea and vomiting comparing tropisetron and control including studies comparing dexamethasone and tropisetron combined with dexamethasone and comparing metoclopramide and tropisetron combined with metoclopramide; Figure S30: Forest plot for post-operative nausea and vomiting comparing tropisetron and metoclopramide; Figure S31: Forest plot for post-operative nausea and vomiting comparing tropisetron and ondansetron; Figure S32: Forest plot for post-operative nausea and vomiting comparing tropisetron and dexamethasone; Figure S33: Forest plot for post-operative nausea and vomiting comparing tropisetron and droperidol; Figure S34: Forest plot for post-operative nausea and vomiting comparing tropisetron and droperidol including studies comparing tropisetron combined with dexamethasone with droperidol combined with dexamethasone; Figure S35: Forest plot for post-operative nausea and vomiting comparing tropisetron and granisetron; Figure S36: Trial sequential analysis plot for post-operative nausea and vomiting comparing tropisetron and metoclopramide; Figure S37: Trial sequential analysis plot for post-operative nausea and vomiting comparing tropisetron and ondansetron; Figure S38: Trial sequential analysis plot for post-operative nausea and vomiting comparing tropisetron and dexamethasone; Figure S39: Trial sequential analysis plot for post-operative nausea and vomiting comparing tropisetron and droperidol; Figure S40: Trial sequential analysis plot for post-operative nausea and vomiting comparing tropisetron and droperidol when including study comparing tropisetron combined with dexamethasone with droperidol combined with dexamethasone; Figure S41: Trial sequential analysis plot for post-operative nausea and vomiting comparing tropisetron and granisetron; Figure S42: Forest plot for use of rescue anti-emetics comparing tropisetron and control groups including studies comparing metoclopramide and tropisetron combined with metoclopramide and comparing dexamethasone and tropisetron combined with dexamethasone and comparing droperidol and tropisetron combined with droperidol; Figure S43: Trial sequential analysis plot for use of rescue anti-emetics comparing tropisetron and control when including studies comparing metoclopramide and tropisetron combined with metoclopramide, dexamethasone, and tropisetron combined with dexamethasone, droperidol, and tropisetron combined with droperidol; Figure S44: Forest plot for use of rescue anti-emetics comparing tropisetron and metoclopramide; Figure S45: Forest plot for use of rescue anti-emetics comparing tropisetron and ondansetron; Figure S46: Forest plot for use of rescue anti-emetics comparing tropisetron and ondansetron when including studies comparing tropisetron combined with droperidol and ondansetron combined with droperidol; Figure S47: Forest plot for use of rescue anti-emetics comparing tropisetron and dexamethasone; Figure S48: Forest plot for use of rescue anti-emetics comparing tropisetron and droperidol; Figure S49: Forest plot for use of rescue anti-emetics comparing tropisetron and granisetron; Figure S50: Forest plot for use of rescue anti-emetics comparing tropisetron and granisetron including studies comparing tropisetron combined with droperidol and granisetron combined with droperidol; Figure S51: Trial sequential analysis plot for use of rescue anti-emetics comparing tropisetron and metoclopramide; Figure S52: Trial sequential analysis plot for use of rescue anti-emetics comparing tropisetron and ondansetron; Figure S53: Trial sequential analysis plot for use of rescue anti-emetics comparing tropisetron and ondansetron including studies comparing tropisetron combined with droperidol and ondansetron combined with droperidol; Figure S54: Trial sequential analysis plot for use of rescue anti-emetics comparing tropisetron and dexamethasone; Figure S55: Trial sequential analysis plot for use of rescue anti-emetics comparing tropisetron and droperidol; Figure S56: Trial sequential analysis plot for use of rescue anti-emetics comparing tropisetron and granisetron including studies comparing tropisetron combined with droperidol and granisetron combined with droperidol; Figure S57: Forest plot for complete response comparing tropisetron and control including studies comparing dexamethasone and tropisetron combined with dexamethasone; Figure S58: Trial sequential analysis plot for complete response comparing tropisetron and control including studies comparing dexamethasone and tropisetron combined with dexamethasone; Figure S59: Forest plot for complete response comparing tropisetron and metoclopramide; Figure S60: Forest plot for complete response comparing tropisetron and ondansetron; Figure S61: Forest plot for complete response comparing tropisetron and dexamethasone; Figure S62: Forest plot for complete response comparing tropisetron and droperidol; Figure S63: Forest plot for complete response comparing tropisetron and droperidol including studies comparing tropisetron combined with dexamethasone with droperidol combined with dexamethasone; Figure S64: Forest plot for complete response comparing tropisetron and granisetron; Figure S65: Trial sequential analysis plot for complete response comparing tropisetron and metoclopramide; Figure S66: Trial sequential analysis plot for complete response comparing tropisetron and ondansetron; Figure S67: Trial sequential analysis plot for complete response comparing tropisetron and dexamethasone; Figure S68: Trial sequential analysis plot for complete response comparing tropisetron and droperidol; Figure S69: Trial sequential analysis plot for complete response comparing tropisetron and droperidol when including studies comparing tropisetron combined with dexamethasone with droperidol combined with dexamethasone; Figure S70: Trial sequential analysis plot for complete response comparing tropisetron, and granisetron. Figure S71: Funnel plot for postoperative nausea compared with control; Figure S72: Funnel plot for postoperative nausea compared with control including studies comparing dexamethasone and tropisetron combined with dexamethasone; Figure S73: Funnel plot for postoperative nausea compared with control including studies comparing dexamethasone and tropisetron combined with dexamethasone; Figure S74: Funnel plot for postoperative vomiting compared with control including studies comparing droperidol and tropisetron combined with droperidol and comparing dexamethasone and tropisetron combined with dexamethasone; Figure S75: Funnel plot for complete response compared with control including studies comparing dexamethasone and tropisetron combined with dexamethasone; Figure S76: Funnel plot for post-operative nausea and vomiting compared with control; Figure S77: Funnel plot for post-operative nausea and vomiting compared with control including studies comparing dexamethasone and tropisetron combined with dexamethasone and comparing metoclopramide and tropisetron combined with metoclopramide; Figure S78: Funnel plot for use of rescue anti-emetics compared with control; Figure S79. Funnel plot for use of rescue anti-emetics compared with control including studies comparing metoclopramide and tropisetron combined with metoclopramide, dexamethasone and tropisetron combined with dexamethasone, droperidol and tropisetron combined with droperidol.
