# Supplementary material for: Effect of Prophylactic Tropisetron on Post-Operative Nausea and Vomiting in Patients Undergoing General Anesthesia: Systematic Review and Meta-Analysis with Trial Sequential Analysis
Source: J Pers Med. 2024 Jul 27;14(8):797. doi: 10.3390/jpm14080797 (PMC11355328; doi:10.3390/jpm14080797)

Search term

Search terms for MEDLINE

1. randomized controlled trial.pt
2. randomized controlled trial$.mp
3. controlled clinical trial.pt
4. controlled clinical trial$.mp
5. random allocation.mp
6. exp double-blind method/
7. double-blind.mp
8. exp single-blind method/
9. single-blind.mp
10. or/1-9
11. clinical trial.pt
12. clinical trial$.mp
13. exp clinical trial/
14. (clin$ adj25 trial$).mp
15. ((singl$ or doubl$ or tripl$ or trebl$) adj25 (blind$ or mask$)).mp
16. random$.mp
17. exp research design/
18. research design.mp
19. or/11-18
20. 10 or 19
21. Case report.tw.
22. Letter.pt.
23. Historical article.pt.
24. Review.pt.
25. or/21-24
26. 20 not 25
27. exp tropisetron/
28. Navoban.mp
29. Setrovel.mp
30. Or/27-29
31. 26 and 30

Search terms for Embase

1. randomi?ed AND controlled AND trial$:ti,ab
2. 'controlled clinical trial (topic)'/exp
3. controlled AND clinical AND trials
4. controlled AND clinical AND trial$:ti,ab
5. 'randomization'/exp
6. 'random allocation'/exp
7. random AND allocation:ti,ab
8. double-blind:ti,ab
9. single-blind:ti,ab
10. #1 OR #2 OR #3 OR #4 OR #5 OR #6 OR #7 OR #8 OR #9
11. 'clinical trial (topic)'/exp
12. clinical AND trial$:ti,ab
13. random$:ti,ab
14. rct
15. #11 OR #12 OR #13 OR #14
16. #10 OR #15
17. 'case study'/exp
18. 'case report'/exp
19. 'abstract report'/exp
20. 'letter'/exp
21. #16 NOT #20
22. ‘tropisetron’/exp
23. Navoban:ti,ab
24. Setrovel:ti,ab
25. #22 OR #23 OR #24
26. #21 AND #25

Supplementary Figure S1. Forest plot for post-operative nausea comparing tropisetron and control including studies comparing dexamethasone and tropisetron combined with dexamethasone. The figure depicts individual trials as filled squares with relative sample size and the 95% confidence interval (CI) of the difference as a solid line. The diamond shape indicates the pooled estimate and uncertainty for the combined effect. The pooled estimate indicates that the incidence of post-operative nausea is lower in tropisetron group than that in control group including studies comparing dexamethasone and tropisetron combined with dexamethasone.

Study name	Statistics for each study			Risk ratio and 95% CI
	Risk ratio	Lower limit	Upper limit	
Zomers, 1993	0.777	0.602	1.004	
Ali-Melkkila, 1996	0.524	0.292	0.939	
Alon, 1996	0.571	0.327	0.998	
Capouet, 1996	0.769	0.613	0.964	
Scholz, 1998	0.830	0.680	1.013	
Chan, 1998	0.572	0.447	0.733	
Jakobsson, 1999	0.923	0.494	1.723	
Tsui, 1999	0.730	0.529	1.007	
Wang, 2002_1	0.513	0.233	1.130	
Wang, 2002_2	0.821	0.444	1.517	
Madenoglu, 2003	0.643	0.330	1.253	
Eberhart, 2006	0.626	0.442	0.887	
Metaxari, 2011	0.577	0.145	2.288	
Eberhart, 2006(2)	1.859	1.123	3.077	
Zhou, 2012	1.909	1.032	3.532	
Yang, 2015	1.893	1.116	3.209	
	0.814	0.683	0.971	

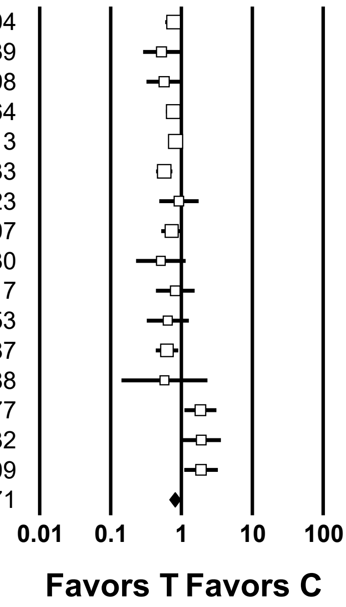

Supplementary Figure S2. Trial sequential analysis plot for post-operative nausea comparing tropisetron and control including studies comparing dexamethasone and tropisetron combined with dexamethasone. Uppermost and lowermost complete red curves represent trial sequential monitoring boundary lines for benefit and harm respectively. Horizontal dotted red line represents the conventional boundaries for statistical significance. Triangular red lines on the right side reflects the futility boundaries. The blue solid line represents the cumulative z-curve. The number on the x-axis indicates required information size (801). The cumulative Z-curve crosses the alpha spending boundary, favoring tropisetron over control for post-operative nausea when including studies comparing dexamethasone and tropisetron combined with dexamethasone.

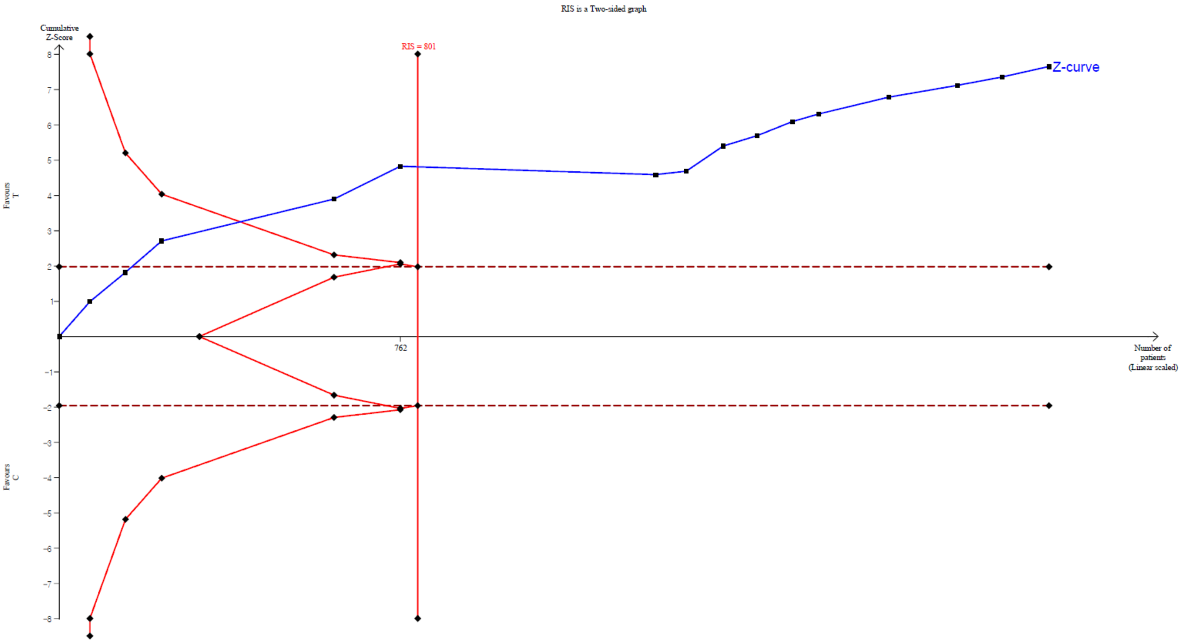

Supplementary Figure S3. Forest plot for post-operative nausea comparing tropisetron and metoclopramide. The figure depicts individual trials as filled squares with relative sample size and the 95% confidence interval (CI) of the difference as a solid line. The diamond shape indicates the pooled estimate and uncertainty for the combined effect. The pooled estimate indicates no significant difference in the incidence of post-operative nausea between tropisetron and metoclopramide.

Study name	Statistics for each study		
	Risk ratio	Lower limit	Upper limit
Ali-Melkkila, 1996	0.786	0.407	1.516
	0.786	0.407	1.516

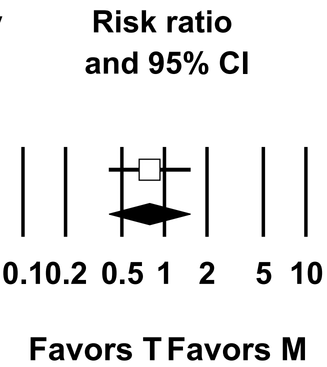

Supplementary Figure S4. Forest plot for post-operative nausea comparing tropisetron and ondansetron. The figure depicts individual trials as filled squares with relative sample size and the 95% confidence interval (CI) of the difference as a solid line. The diamond shape indicates the pooled estimate and uncertainty for the combined effect. The pooled estimate indicates no significant difference in the incidence of post-operative nausea between tropisetron and ondansetron

Study name	Statistics for each study			Risk ratio and 95% CI
	Risk ratio	Lower limit	Upper limit	
Scholz, 1998	1.018	0.819	1.266	
Tsui, 1999	0.820	0.577	1.164	
Quan, 2007 (Abstract)	1.130	0.718	1.779	
Metaxari, 2011	0.981	0.208	4.635	
	0.981	0.828	1.163	

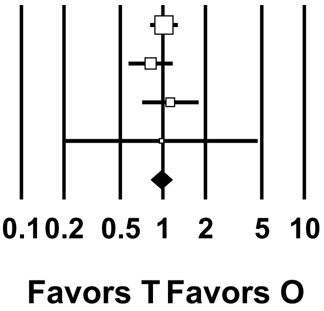

Supplementary Figure S5. Forest plot for post-operative nausea comparing tropisetron and ondansetron including study comparing tropisetron combined with droperidol and ondansetron combined with droperidol. The figure depicts individual trials as filled squares with relative sample size and the 95% confidence interval (CI) of the difference as a solid line. The diamond shape indicates the pooled estimate and uncertainty for the combined effect. The pooled estimate indicates no significant difference in the incidence of post-operative nausea between tropisetron and ondansetron when including study comparing tropisetron combined with droperidol and ondansetron combined with droperidol

Study name	Statistics for each study		
	Risk ratio	Lower limit	Upper limit
Scholz, 1998	1.018	0.819	1.266
Tsui, 1999	0.820	0.577	1.164
Quan, 2007 (Abstract)	1.130	0.718	1.779
Metaxari, 2011	0.981	0.208	4.635
Koivuranta, 1999	1.374	0.835	2.261
	1.016	0.865	1.194

Risk ratio and 95% CI

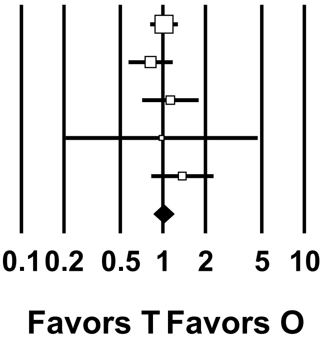

Supplementary Figure S6. Forest plot for post-operative nausea comparing tropisetron and dexamethasone. The figure depicts individual trials as filled squares with relative sample size and the 95% confidence interval (CI) of the difference as a solid line. The diamond shape indicates the pooled estimate and uncertainty for the combined effect. The pooled estimate indicates no significant difference in the incidence of post-operative nausea between tropisetron and dexamethasone

Study name	Statistics for each study			Risk ratio and 95% CI
	Risk ratio	Lower limit	Upper limit	
Wang, 2002_1	1.167	0.432	3.150	
Wang, 2002_2	2.053	0.858	4.910	
Eberhart, 2006	0.906	0.607	1.354	
Zhou, 2012	0.810	0.488	1.342	
	0.994	0.718	1.375	

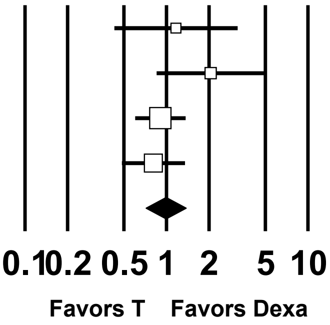

Supplementary Figure S7. Forest plot for post-operative nausea comparing tropisetron and droperidol. The figure depicts individual trials as filled squares with relative sample size and the 95% confidence interval (CI) of the difference as a solid line. The diamond shape indicates the pooled estimate and uncertainty for the combined effect. The pooled estimate indicates no significant difference in the incidence of post-operative nausea between tropisetron and droperidol

Study name	Statistics for each study			Risk ratio and 95% CI
	Risk ratio	Lower limit	Upper limit	
Jokela, 1999	0.892	0.658	1.208	
	0.892	0.658	1.208	

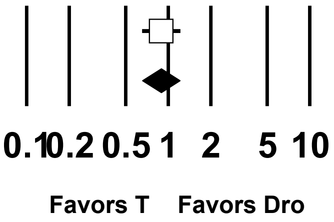

Supplementary Figure S8. Forest plot for post-operative nausea comparing tropisetron and granisetron. The figure depicts individual trials as filled squares with relative sample size and the 95% confidence interval (CI) of the difference as a solid line. The diamond shape indicates the pooled estimate and uncertainty for the combined effect. The pooled estimate indicates no significant difference in the incidence of post-operative nausea between tropisetron and granisetron.

Study name	Statistics for each study			Risk ratio and 95% CI
	Risk ratio	Lower limit	Upper limit	
Quan, 2007	1.250	0.780	2.004	
Metaxari, 2011	2.885	0.310	26.817	
	1.296	0.816	2.057	

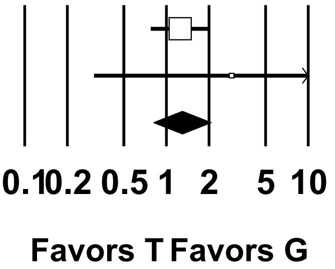

Supplementary Figure S9. Trial sequential analysis plot for post-operative nausea comparing tropisetron and ondansetron. Uppermost and lowermost complete red curves represent trial sequential monitoring boundary lines for benefit and harm respectively. Horizontal dotted red line represents the conventional boundaries for statistical significance. Triangular red lines on the right side reflects the futility boundaries. The blue solid line represents the cumulative z-curve. The number on the x-axis indicates required information size. The TSA suggests insufficient evidence, with only 63.1% of the required information size (RIS) accrued, as the Z-curve crossed futility boundary.

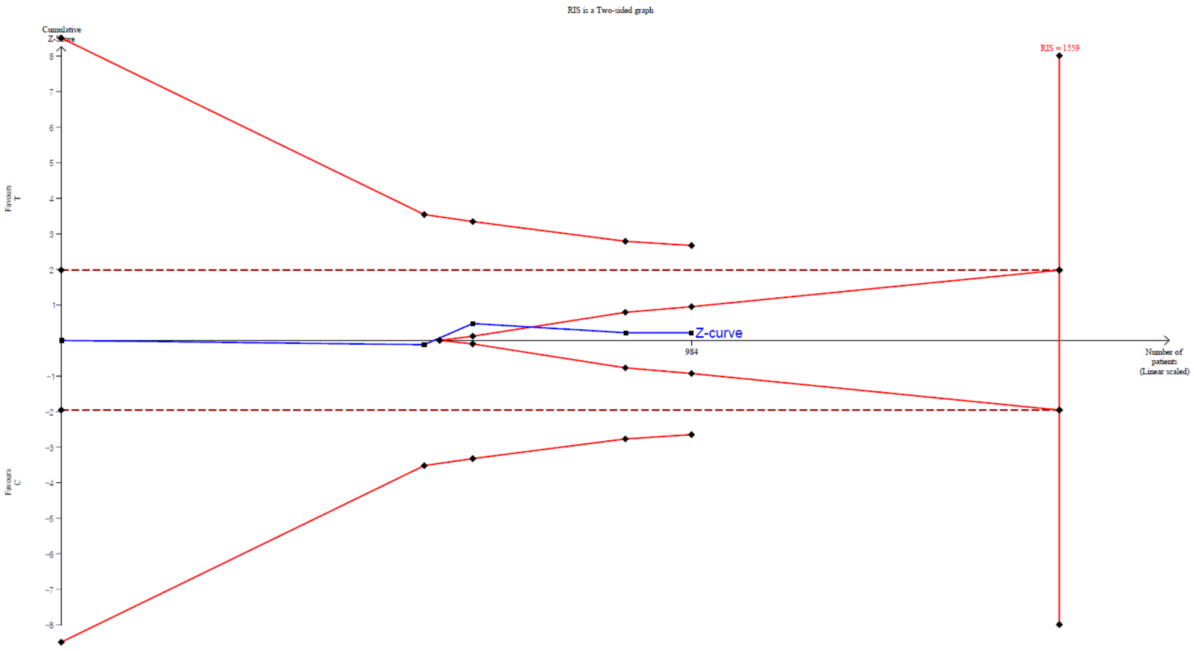

Supplementary Figure S10. Trial sequential analysis plot for post-operative nausea comparing tropisetron and ondansetron when including study comparing Tropisetron combined with Droperidol and Tropisetron combined with ondansetron. Uppermost and lowermost complete red curves represent trial sequential monitoring boundary lines for benefit and harm respectively. Horizontal dotted red line represents the conventional boundaries for statistical significance. Triangular red lines on the right side reflects the futility boundaries. The blue solid line represents the cumulative z-curve. The number on the x-axis indicates required information size. The TSA suggests insufficient evidence, with only 69.7% of the required information size (RIS) accrued, as the Z-curve crossed futility boundary.

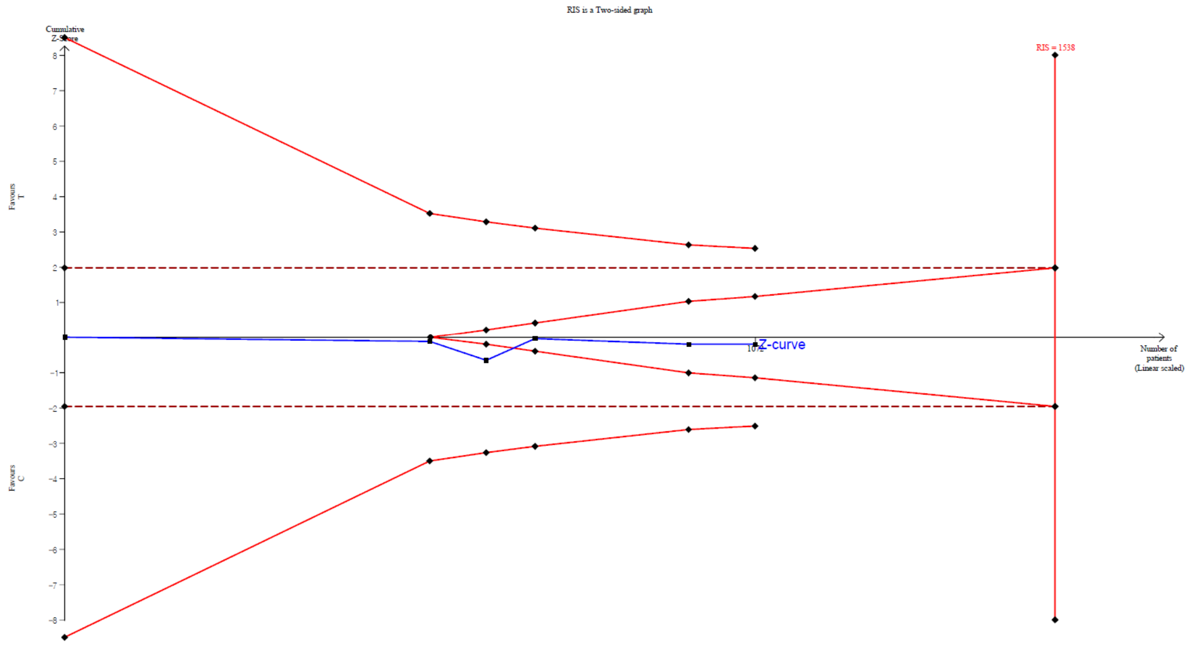

Supplementary Figure S11. Trial sequential analysis plot for post-operative nausea comparing tropisetron and dexamethasone. Horizontal dotted red line represents the conventional boundaries for statistical significance. The blue solid line represents the cumulative z-curve. The number on the x-axis indicates required information size. The TSA suggests insufficient evidence, with only 20.2% of the required information size (RIS) accrued, as the Z-curve but crossed neither the conventional test boundary nor cross the trial sequential monitoring boundary.

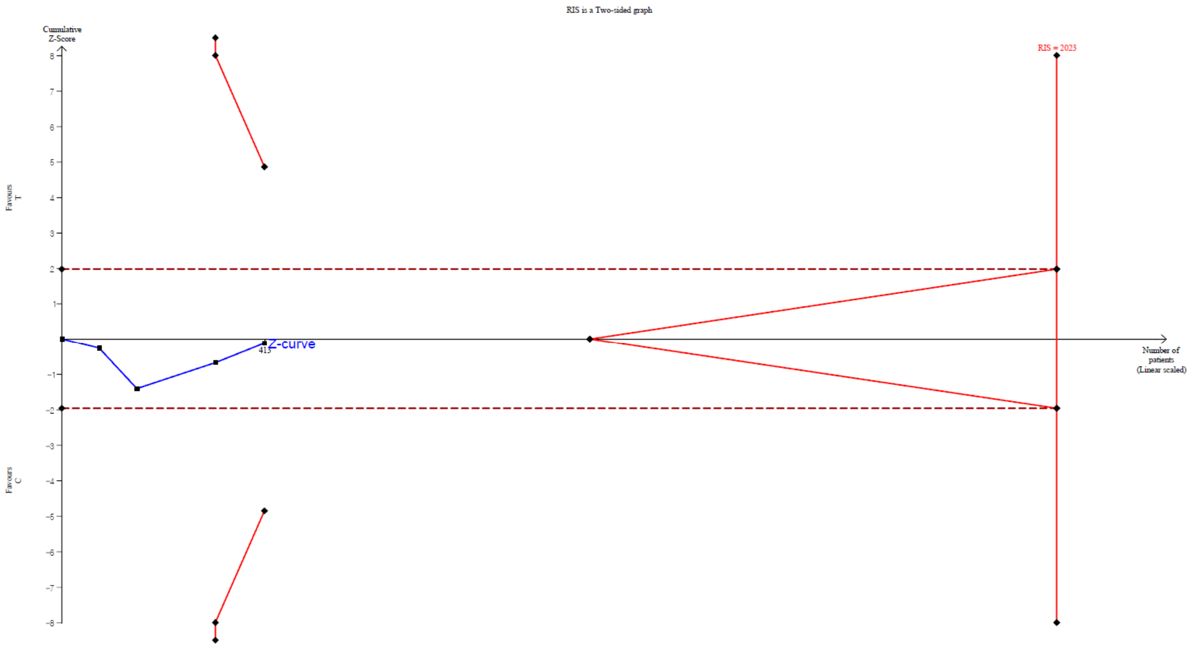

Supplementary Figure S12. Trial sequential analysis plot for post-operative nausea comparing tropisetron and granisetron. Uppermost and lowermost complete red curves represent trial sequential monitoring boundary lines for benefit and harm respectively. Horizontal dotted red line represents the conventional boundaries for statistical significance. Triangular red lines on the right side reflects the futility boundaries. The blue solid line represents the cumulative z-curve. The number on the x-axis indicates required information size. The TSA suggests insufficient evidence, with only 8.2 % of the required information size (RIS) accrued, as the Z-curve crossed neither the conventional test boundary nor cross the trial sequential monitoring boundary.

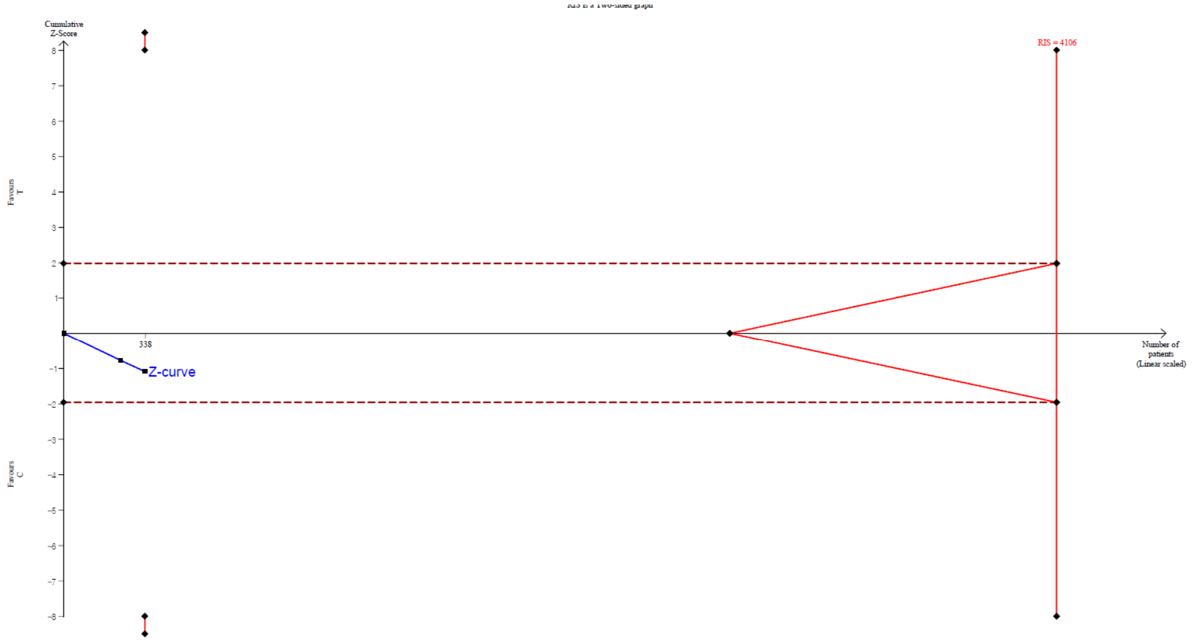

Supplementary Figure S13. Forest plot for post-operative vomiting comparing tropisetron and control including studies comparing droperidol and tropisetron combined with droperidol and comparing dexamethasone and tropisetron combined with dexamethasone. The figure depicts individual trials as filled squares with relative sample size and the 95% confidence interval (CI) of the difference as a solid line. The diamond shape indicates the pooled estimate and uncertainty for the combined effect. The pooled estimate indicates that the incidence of post-operative vomiting is lower in tropisetron group than that in control group including studies comparing droperidol and tropisetron combined with droperidol and comparing dexamethasone and tropisetron combined with dexamethasone.

Study name	Statistics for each study		
	Risk ratio	Lower limit	Upper limit
Zomers, 1993	0.437	0.233	0.820
Ali-Melkkila, 1996	0.917	0.459	1.829
Alon, 1996	0.438	0.202	0.947
Capouet, 1996	0.658	0.494	0.876
Purhonen, 1997	0.328	0.174	0.620
Scholz, 1998	1.128	0.860	1.480
Chan, 1998	0.462	0.350	0.609
Tsui, 1999	0.405	0.194	0.847
Jakobsson, 1999	1.333	0.322	5.513
Wang, 2002_1	0.373	0.130	1.070
Wang, 2002_2	0.924	0.423	2.019
Madenoglu, 2003	0.444	0.229	0.861
Eberhart, 2006	0.633	0.366	1.097
Metaxari, 2011	0.321	0.013	7.693
Eberhart, 2006(2)	0.475	0.170	1.324
Zhou, 2012	0.500	0.236	1.061
Papadima, 2013	0.770	0.403	1.470
Yang, 2015	0.629	0.281	1.409
	0.592	0.479	0.732

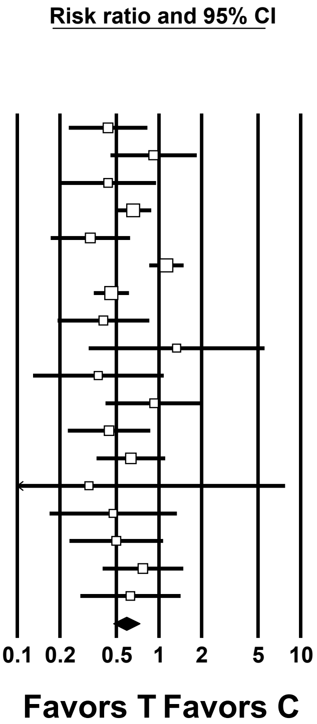

Supplementary Figure S14. Trial sequential analysis plot for post-operative vomiting comparing tropisetron and control including studies comparing droperidol and tropisetron combined with droperidol and comparing dexamethasone and tropisetron combined with dexamethasone. Uppermost and lowermost complete red curves represent trial sequential monitoring boundary lines for benefit and harm respectively. Horizontal dotted red line represents the conventional boundaries for statistical significance. Triangular red lines on the right side reflects the futility boundaries. The blue solid line represents the cumulative z-curve. The number on the x-axis indicates required information size (3947). The cumulative Z-curve crosses the alpha spending boundary, favoring tropisetron over control for post-operative vomiting when including studies comparing droperidol and tropisetron combined with droperidol and comparing dexamethasone and tropisetron combined with dexamethasone.

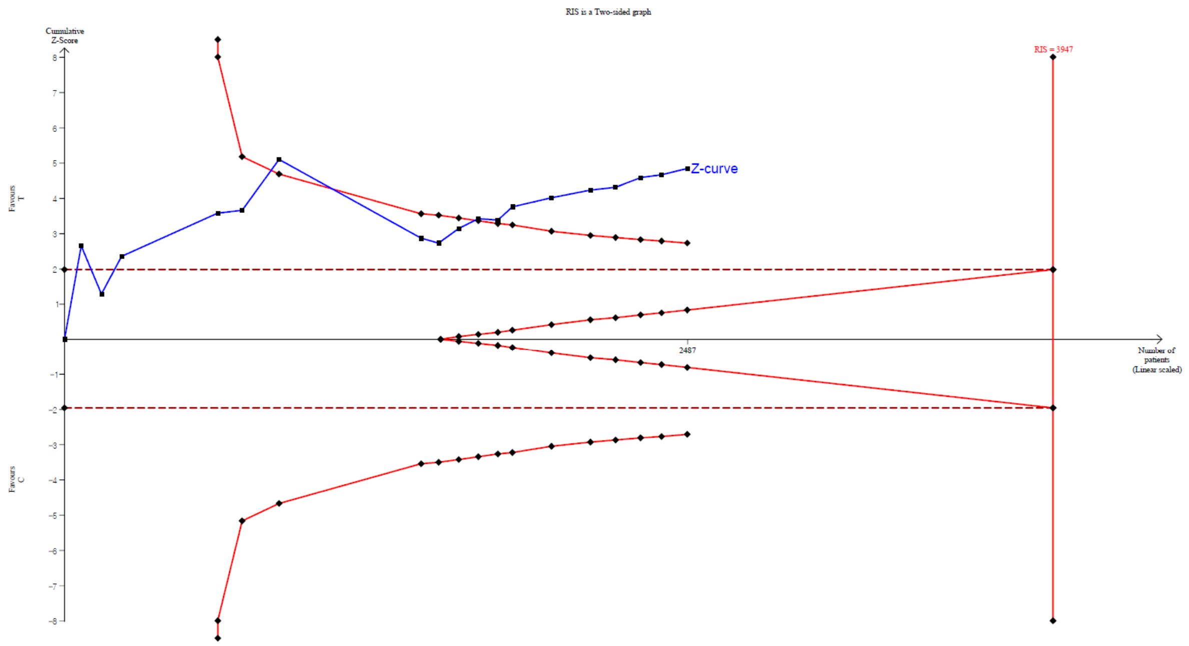

Supplementary Figure S15. Forest plot for post-operative vomiting comparing tropisetron and droperidol. The figure depicts individual trials as filled squares with relative sample size and the 95% confidence interval (CI) of the difference as a solid line. The diamond shape indicates the pooled estimate and uncertainty for the combined effect. The pooled estimate indicates significant difference in the incidence of post-operative vomiting between tropisetron and droperidol.

Study name	Statistics for each study			Risk ratio and 95% CI
	Risk ratio	Lower limit	Upper limit	
Purhonen, 1997	0.418	0.215	0.813	
Jokela, 1999	0.387	0.221	0.679	
	0.400	0.260	0.614	

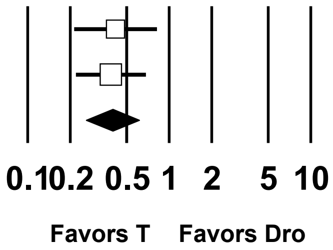

Supplementary Figure S16. Forest plot for post-operative vomiting comparing tropisetron and metoclopramide. The figure depicts individual trials as filled squares with relative sample size and the 95% confidence interval (CI) of the difference as a solid line. The diamond shape indicates the pooled estimate and uncertainty for the combined effect. The pooled estimate indicates no significant difference in the incidence of post-operative vomiting between tropisetron and metoclopramide.

Study name	Statistics for each study		
	Risk ratio	Lower limit	Upper limit
Ali-Melkkila, 1996	1.833	0.751	4.478
	1.833	0.751	4.478

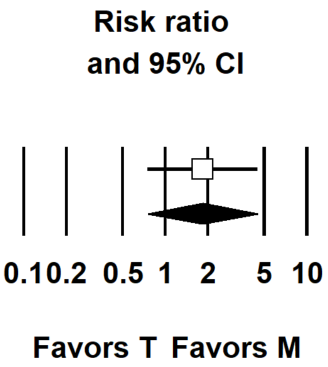

Supplementary Figure S17. Forest plot for post-operative vomiting comparing tropisetron and ondansetron. The figure depicts individual trials as filled squares with relative sample size and the 95% confidence interval (CI) of the difference as a solid line. The diamond shape indicates the pooled estimate and uncertainty for the combined effect. The pooled estimate indicates no significant difference in the incidence of post-operative vomiting between tropisetron and ondansetron.

Study name	Statistics for each study			Risk ratio and 95% CI
	Risk ratio	Lower limit	Upper limit	
Scholz, 1998	1.128	0.860	1.480	
Tsui, 1999	0.461	0.214	0.992	
Argiriadou, 2002	0.702	0.172	2.870	
Wang, 2002_1	1.000	0.270	3.710	
Quan,2007	1.356	0.671	2.742	
Metaxari, 2011	0.196	0.010	3.990	
	0.940	0.649	1.361	

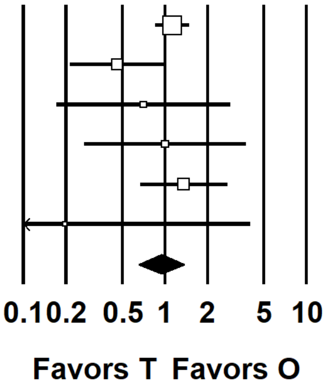

Supplementary Figure S18. Forest plot for post-operative vomiting comparing tropisetron and ondansetron including study comparing tropisetron combined with droperidol and ondansetron combined with droperidol. The figure depicts individual trials as filled squares with relative sample size and the 95% confidence interval (CI) of the difference as a solid line. The diamond shape indicates the pooled estimate and uncertainty for the combined effect. The pooled estimate indicates no significant difference in the incidence of post-operative vomiting between tropisetron and ondansetron when including study comparing tropisetron combined with droperidol and ondansetron combined with droperidol.

Study name	Statistics for each study		
	Risk ratio	Lower limit	Upper limit
Scholz, 1998	1.128	0.860	1.480
Tsui, 1999	0.461	0.214	0.992
Argiriadou, 2002	0.702	0.172	2.870
Wang, 2002_1	1.000	0.270	3.710
Quan,2007	1.356	0.671	2.742
Metaxari, 2011	0.196	0.010	3.990
Koivuranta, 1999	1.047	0.366	2.995
	0.989	0.742	1.319

Risk ratio and 95% CI

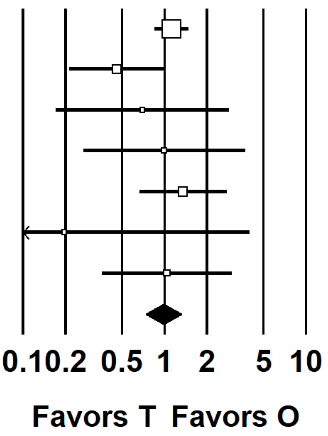

Supplementary Figure S19. Forest plot for post-operative vomiting comparing tropisetron and dexamethasone. The figure depicts individual trials as filled squares with relative sample size and the 95% confidence interval (CI) of the difference as a solid line. The diamond shape indicates the pooled estimate and uncertainty for the combined effect. The pooled estimate indicates no significant difference in the incidence of post-operative vomiting between tropisetron and dexamethasone.

Study name	Statistics for each study			Risk ratio and 95% CI
	Risk ratio	Lower limit	Upper limit	
Wang, 2002_2	2.309	0.777	6.867	
Eberhart, 2006	1.500	0.727	3.096	
Zhou, 2012	0.938	0.522	1.683	
	1.286	0.803	2.058	

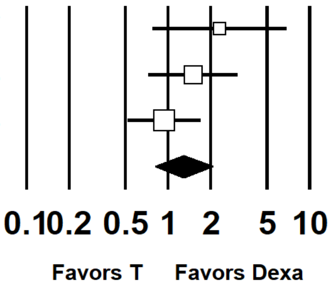

Supplementary Figure S20. Forest plot for post-operative vomiting comparing tropisetron and granisetron. The figure depicts individual trials as filled squares with relative sample size and the 95% confidence interval (CI) of the difference as a solid line. The diamond shape indicates the pooled estimate and uncertainty for the combined effect. The pooled estimate indicates no significant difference in the incidence of post-operative vomiting between tropisetron and granisetron.

Study name	Statistics for each study			Risk ratio and 95% CI
	Risk ratio	Lower limit	Upper limit	
Quan, 2007	1.600	0.757	3.380	
Metaxari, 2011	0.962	0.000	6108.300	
	1.594	0.757	3.358	

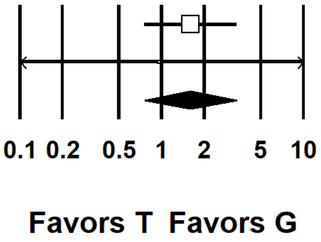

Supplementary Figure S21. Forest plot for post-operative vomiting comparing tropisetron and granisetron including study comparing tropisetron combined with droperidol and granisetron combined with droperidol**.** The figure depicts individual trials as filled squares with relative sample size and the 95% confidence interval (CI) of the difference as a solid line. The diamond shape indicates the pooled estimate and uncertainty for the combined effect. The pooled estimate indicates no significant difference in the incidence of post-operative vomiting between tropisetron and granisetron when including study comparing tropisetron combined with droperidol and granisetron combined with droperidol.

Study name	Statistics for each study		
	Risk ratio	Lower limit	Upper limit
Quan, 2007	1.600	0.757	3.380
Metaxari, 2011	0.962	0.000	6108.300
Papadima, 2013	1.238	0.589	2.601
	1.404	0.830	2.376

Risk ratio and 95% CI

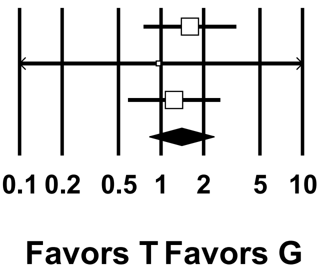

Supplementary Figure S22. Trial sequential analysis plot for post-operative vomiting comparing tropisetron and ondansetron. Horizontal dotted red line represents the conventional boundaries for statistical significance. The blue solid line represents the cumulative z-curve. The number on the x-axis indicates required information size. The TSA suggests insufficient evidence, with only 19.8 % of the required information size (RIS) accrued, as the Z-curve but crossed neither the conventional test boundary nor cross the trial sequential monitoring boundary.

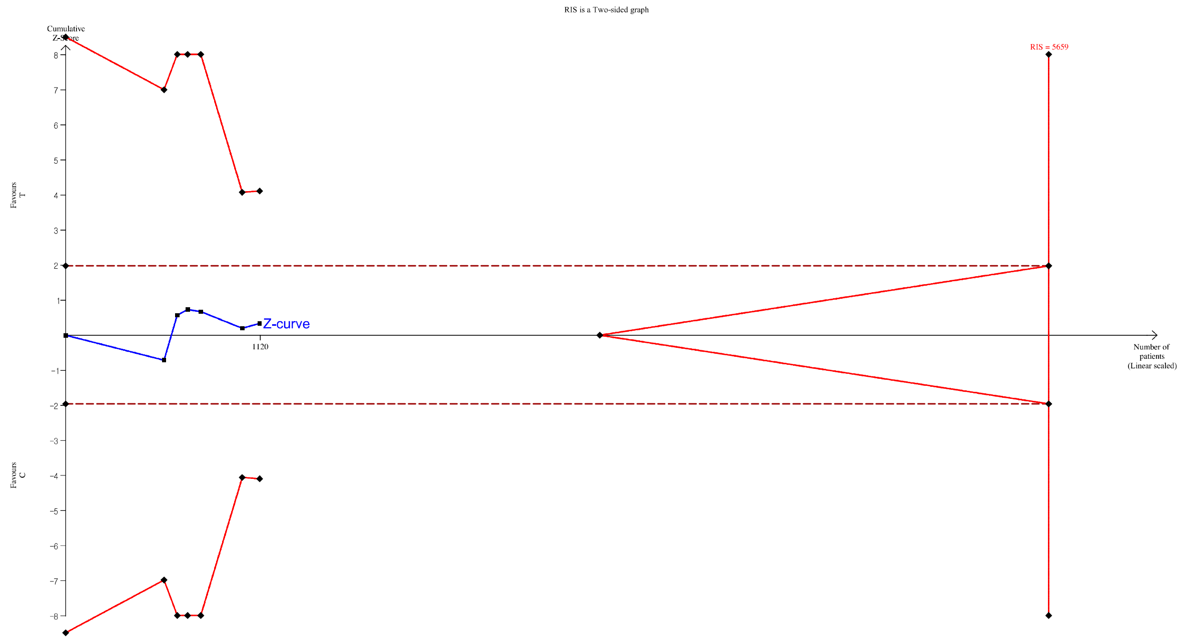

Supplementary Figure S23. Trial sequential analysis plot for post-operative vomiting comparing tropisetron and ondansetron when including study comparing tropisetron combined with droperidol and ondansetron combined with droperidol. Horizontal dotted red line represents the conventional boundaries for statistical significance. The blue solid line represents the cumulative z-curve. The number on the x-axis indicates required information size. The TSA suggests insufficient evidence, with only 24.4% of the required information size (RIS) accrued, as the Z-curve but crossed neither the conventional test boundary nor cross the trial sequential monitoring boundary.

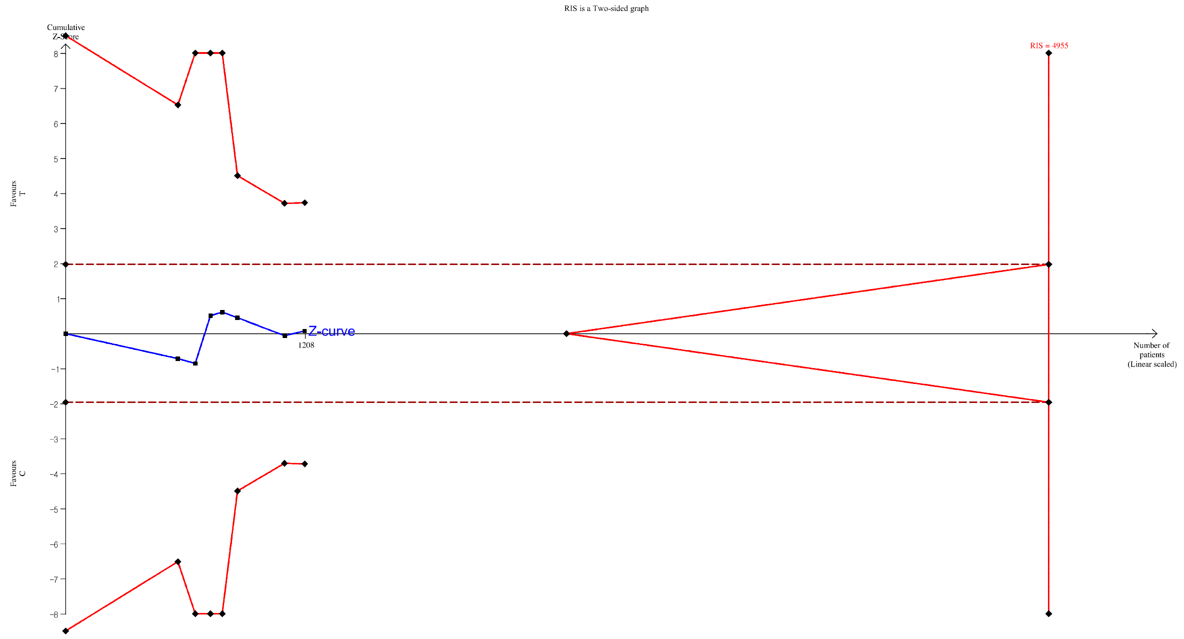

Supplementary Figure S24. Trial sequential analysis plot for post-operative vomiting comparing tropisetron and dexamethasone. Horizontal dotted red line represents the conventional boundaries for statistical significance. The blue solid line represents the cumulative z-curve. The number on the x-axis indicates required information size. The TSA suggests insufficient evidence, with only 8.1% of the required information size (RIS) accrued, as the Z-curve but crossed neither the conventional test boundary nor cross the trial sequential monitoring boundary.

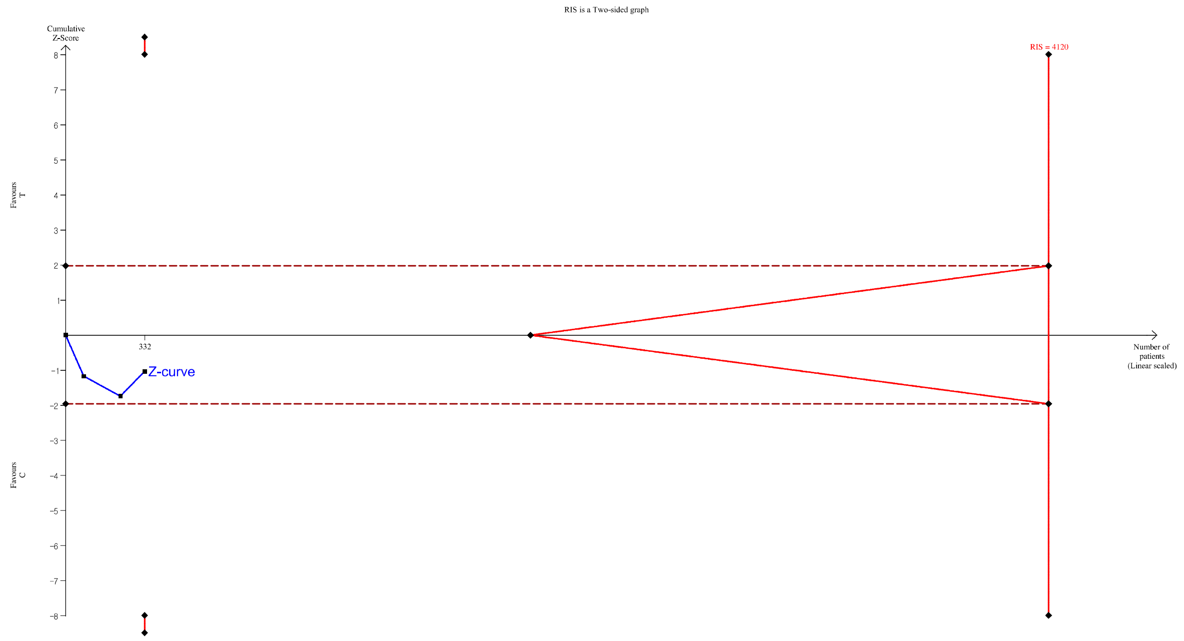

Supplementary Figure S25. Trial sequential analysis plot for post-operative vomiting comparing tropisetron and droperidol. Horizontal dotted red line represents the conventional boundaries for statistical significance. The blue solid line represents the cumulative z-curve. The number on the x-axis indicates required information size. The TSA suggests insufficient evidence, with only 26.5% of the required information size (RIS) accrued and as **Z-curve crossed the conventional test boundary and met the trial sequential monitoring boundary (represented by the complete red curve).**

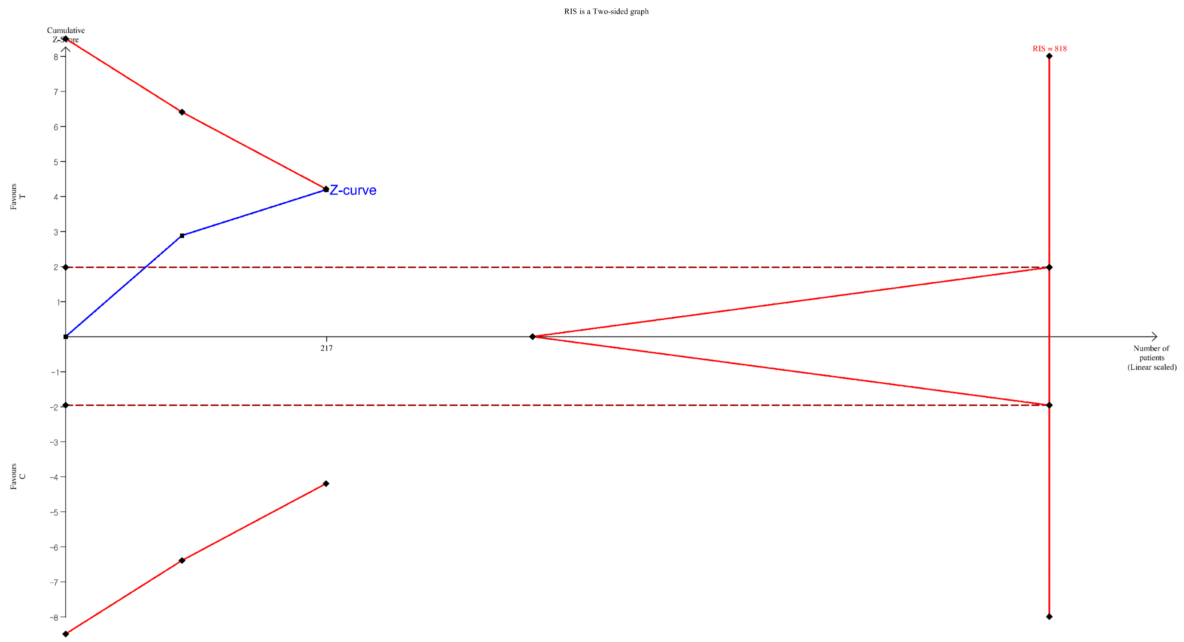

Supplementary Figure S26. Trial sequential analysis plot for post-operative vomiting comparing tropisetron and granisetron. Horizontal dotted red line represents the conventional boundaries for statistical significance. The blue solid line represents the cumulative z-curve. The number on the x-axis indicates required information size. The TSA suggests insufficient evidence, with only 6.8% of the required information size (RIS) accrued, as the Z-curve but crossed neither the conventional test boundary nor cross the trial sequential monitoring boundary.

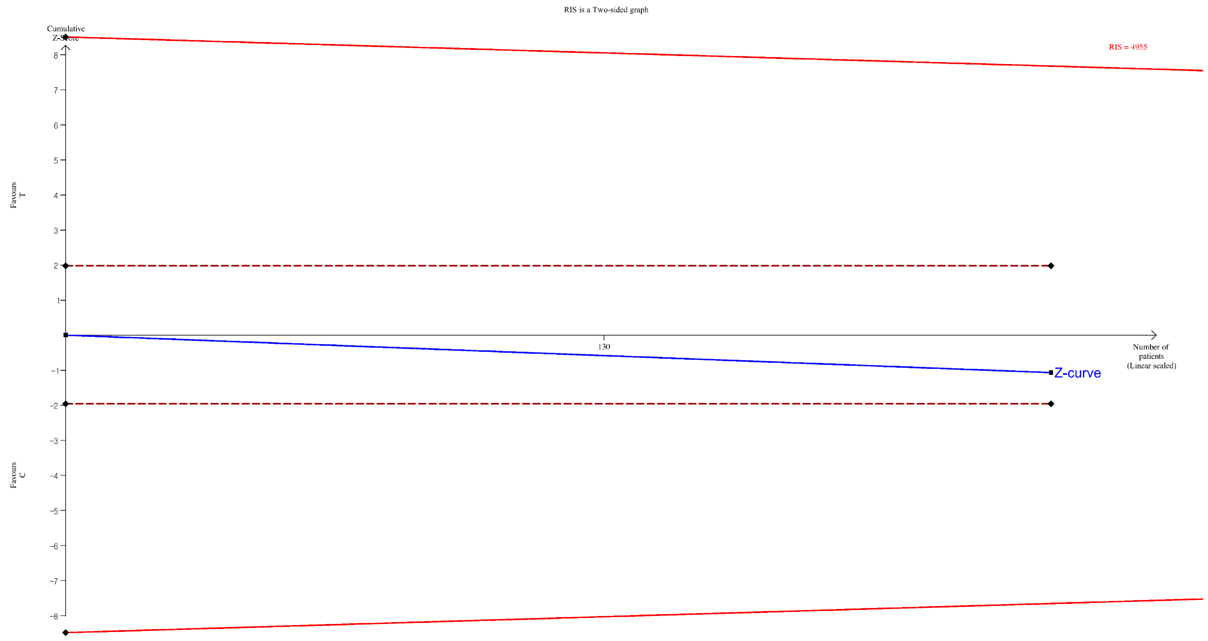

Supplementary Figure S27. Trial sequential analysis plot for post-operative vomiting comparing tropisetron and graniseetron when including study comparing tropisetron combined with droperidol and granisetron combined with droperidol. Horizontal dotted red line represents the conventional boundaries for statistical significance. The blue solid line represents the cumulative z-curve. The number on the x-axis indicates required information size. The TSA suggests insufficient evidence, with only 6.1% of the required information size (RIS) accrued, as the Z-curve but crossed neither the conventional test boundary nor cross the trial sequential monitoring boundary.

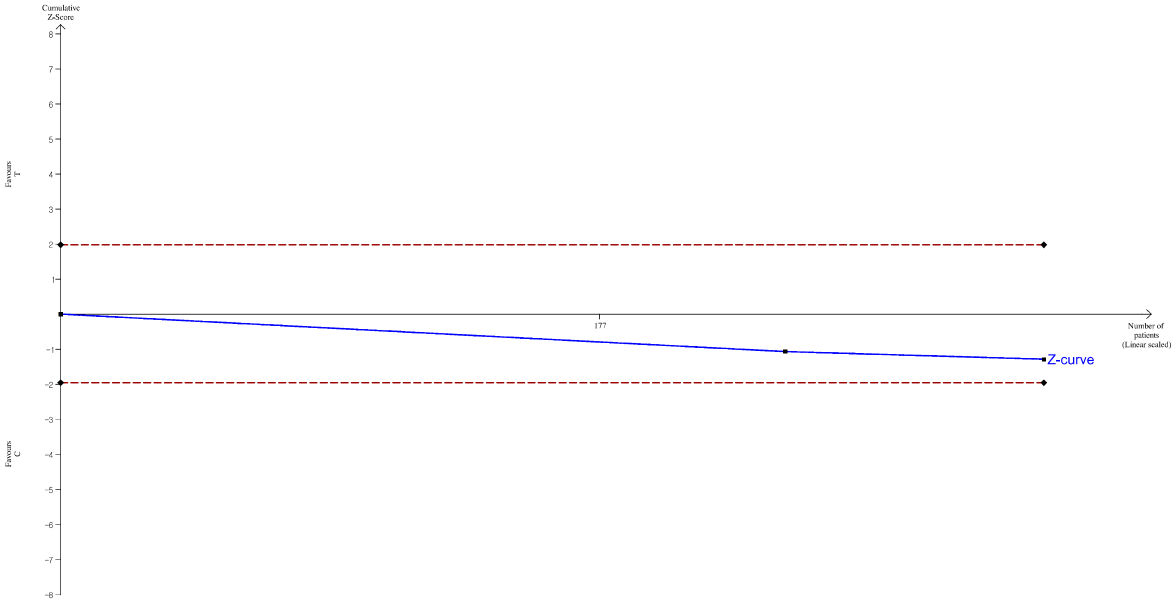

Supplementary Figure S28. Forest plot for post-operative nausea and vomiting comparing tropisetron and control including studies comparing dexamethasone and tropisetron combined with dexamethasone and comparing metoclopramide and tropisetron combined with metoclopramide. The figure depicts individual trials as filled squares with relative sample size and the 95% confidence interval (CI) of the difference as a solid line. The diamond shape indicates the pooled estimate and uncertainty for the combined effect. The pooled estimate indicates that the incidence of post-operative nausea and vomiting is lower in tropisetron group than that in control group including studies dexamethasone and tropisetron combined with dexamethasone and comparing metoclopramide and tropisetron combined with metoclopramide.

Study name	Statistics for each study		
	Risk ratio	Lower limit	Upper limit
Alon, 1996	0.583	0.357	0.954
Naguib, 1996	0.718	0.463	1.113
Purhonen, 1997	1.076	0.873	1.327
Muhammad, 2000	0.205	0.053	0.787
Papadimitriou, 2001	0.388	0.194	0.776
Wang, 2002_2	0.862	0.595	1.248
Eberhart, 2006(1)	0.633	0.451	0.889
Eberhart, 2006(2)	0.570	0.348	0.931
Akin, 2006	0.556	0.364	0.848
Contreras-Dominguez, 2008	0.429	0.125	1.472
Ekinci, 2011	0.692	0.387	1.238
Mei, 2014(Sevo)	0.333	0.169	0.659
Mei, 2014(Propofol)	0.338	0.129	0.882
Yang, 2015	0.528	0.312	0.896
YI, 2022	0.707	0.487	1.024
	0.614	0.504	0.749

Risk ratio and 95% CI

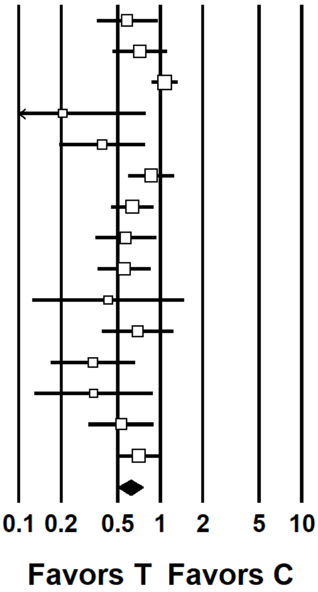

Supplementary Figure S29. Trial sequential analysis plot for post-operative nausea and vomiting comparing tropisetron and control including studies comparing dexamethasone and tropisetron combined with dexamethasone and comparing metoclopramide and tropisetron combined with metoclopramide. Uppermost and lowermost complete red curves represent trial sequential monitoring boundary lines for benefit and harm respectively. Horizontal dotted red line represents the conventional boundaries for statistical significance. Triangular red lines on the right side reflects the futility boundaries. The blue solid line represents the cumulative z-curve. The number on the x-axis indicates required information size (3947). The cumulative Z-curve crosses the alpha spending boundary, favoring tropisetron over control for post-operative nausea and vomiting when including studies comparing dexamethasone and tropisetron combined with dexamethasone and comparing metoclopramide and tropisetron combined with metoclopramide.

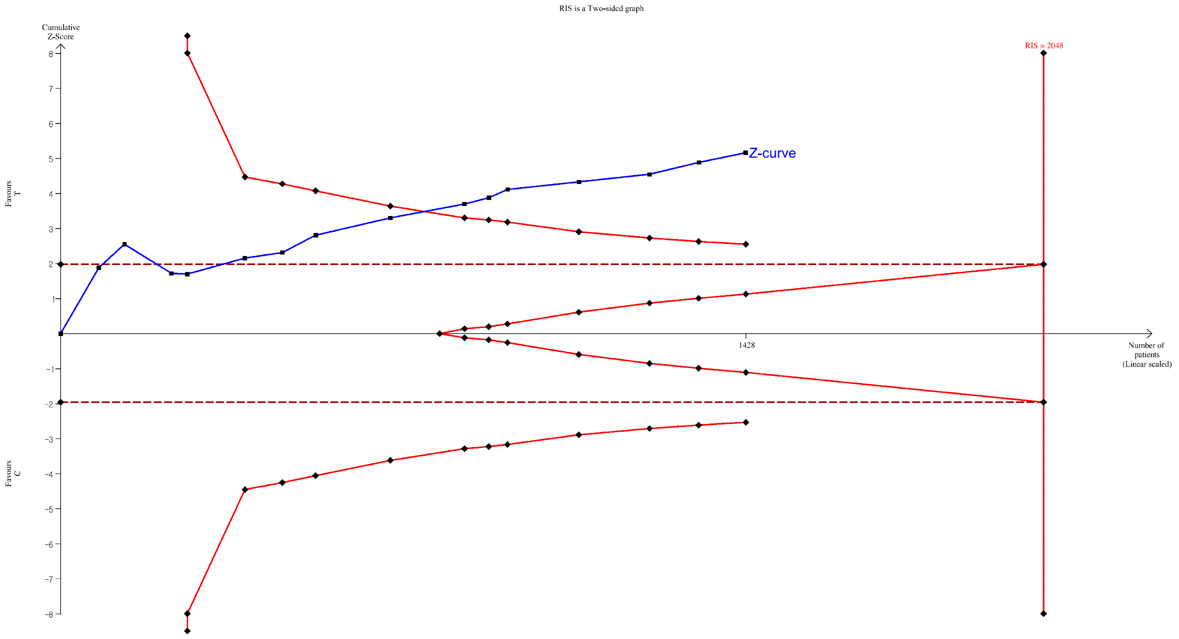

Supplementary Figure S30. Forest plot for post-operative nausea and vomiting comparing tropisetron and metoclopramide. The figure depicts individual trials as filled squares with relative sample size and the 95% confidence interval (CI) of the difference as a solid line. The diamond shape indicates the pooled estimate and uncertainty for the combined effect. The pooled estimate indicates significant difference in the incidence of post-operative nausea and vomiting between tropisetron and metoclopramide.

Study name	Statistics for each study			Risk ratio and 95% CI
	Risk ratio	Lower limit	Upper limit	
Naguib, 1996	0.734	0.465	1.158	
Muhammad, 2000	0.222	0.057	0.871	
Jokela, 2002	0.782	0.603	1.015	
Contreras-Dominguez, 2008	1.000	0.223	4.487	
Ekinci, 2011	1.000	0.504	1.984	
	0.770	0.624	0.950	

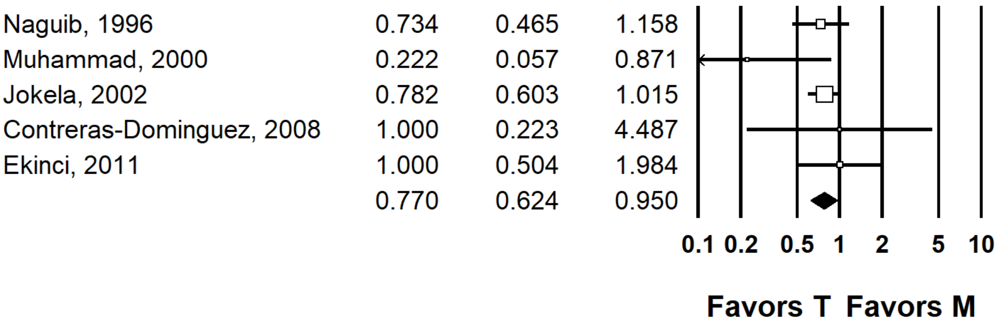

Supplementary Figure S31. Forest plot for post-operative nausea and vomiting comparing tropisetron and ondansetron. The figure depicts individual trials as filled squares with relative sample size and the 95% confidence interval (CI) of the difference as a solid line. The diamond shape indicates the pooled estimate and uncertainty for the combined effect. The pooled estimate indicates no significant difference in the incidence of post-operative nausea and vomiting between tropisetron and ondansetron.

Study name	Statistics for each study		
	Risk ratio	Lower limit	Upper limit
Naguib, 1996	1.508	0.805	2.824
Jokela, 2002	0.854	0.649	1.123
Ekinci, 2011	1.286	0.596	2.774
	1.063	0.727	1.553

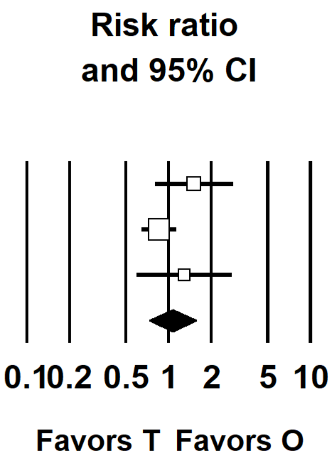

Supplementary Figure S32. Forest plot for post-operative nausea and vomiting comparing tropisetron and dexamethasone. The figure depicts individual trials as filled squares with relative sample size and the 95% confidence interval (CI) of the difference as a solid line. The diamond shape indicates the pooled estimate and uncertainty for the combined effect. The pooled estimate indicates no significant difference in the incidence of post-operative nausea and vomiting between tropisetron and dexamethasone.

Study name	Statistics for each study			Risk ratio and 95% CI
	Risk ratio	Lower limit	Upper limit	
Wang, 2002_2	2.155	1.176	3.952	
Eberhart, 2006	0.938	0.631	1.393	
Contreras-Dominguez, 2008	0.500	0.140	1.781	
	1.129	0.552	2.306	

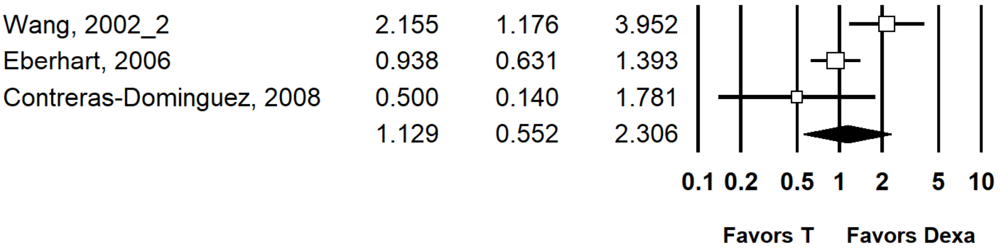

Supplementary Figure S33. Forest plot for post-operative nausea and vomiting comparing tropisetron and droperidol. The figure depicts individual trials as filled squares with relative sample size and the 95% confidence interval (CI) of the difference as a solid line. The diamond shape indicates the pooled estimate and uncertainty for the combined effect. The pooled estimate indicates no significant difference in the incidence of post-operative nausea and vomiting between tropisetron and droperidol.

Study name	Statistics for each study			Risk ratio and 95% CI
	Risk ratio	Lower limit	Upper limit	
Purhonen, 1997	0.967	0.774	1.208	
Contreras-Dominguez, 2008	3.000	0.334	26.919	
Ekinci, 2011	0.900	0.468	1.730	
	0.970	0.787	1.196	

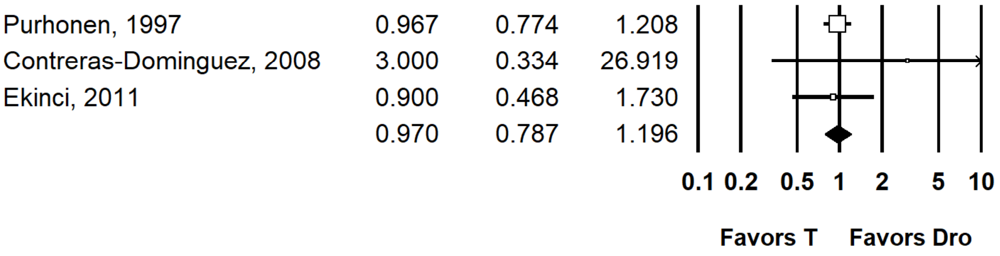

Supplementary Figure S34. Forest plot for post-operative nausea and vomiting comparing tropisetron and droperidol including study comparing tropisetron combined with dexamethasone with droperidol combined with dexamethasone. The figure depicts individual trials as filled squares with relative sample size and the 95% confidence interval (CI) of the difference as a solid line. The diamond shape indicates the pooled estimate and uncertainty for the combined effect. The pooled estimate indicates no significant difference in the incidence of post-operative nausea and vomiting between tropisetron and droperidol including study comparing tropisetron combined with dexamethasone with droperidol combined with dexamethasone.

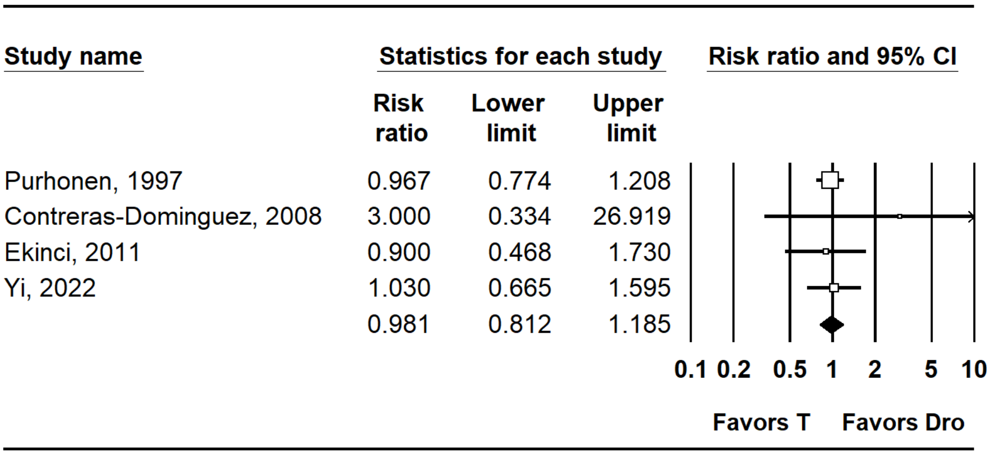

Supplementary Figure S35. Forest plot for post-operative nausea and vomiting comparing tropisetron and granisetron. The figure depicts individual trials as filled squares with relative sample size and the 95% confidence interval (CI) of the difference as a solid line. The diamond shape indicates the pooled estimate and uncertainty for the combined effect. The pooled estimate indicates no significant difference in the incidence of post-operative nausea and vomiting between tropisetron and granisetron.

Study name	Statistics for each study		
	Risk ratio	Lower limit	Upper limit
Naguib, 1996	1.083	0.622	1.888
Contreras-Dominguez, 2008	1.000	0.223	4.487
	1.073	0.637	1.806

Risk ratio and 95% CI

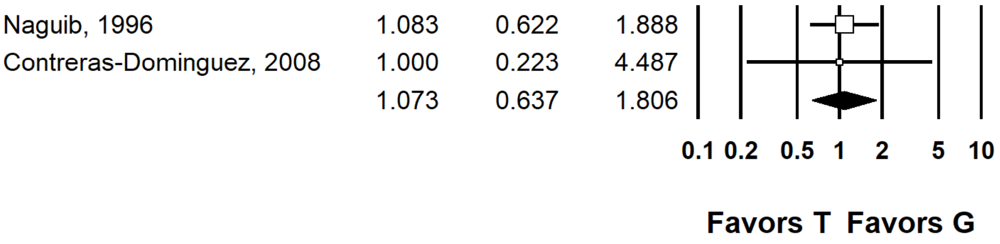

Supplementary Figure S36. Trial sequential analysis plot for post-operative nausea and vomiting comparing tropisetron and metoclopramide. Uppermost and lowermost complete red curves represent trial sequential monitoring boundary lines for benefit and harm respectively. Horizontal dotted red line represents the conventional boundaries for statistical significance. Triangular red lines on the right side reflects the futility boundaries. The blue solid line represents the cumulative z-curve. The number on the x-axis indicates required information size. The TSA **suggests insufficient evidence, with only 46.9 % of the required information size (RIS) accrued, as the Z-curve crossed the conventional test boundary but did not cross the trial sequential monitoring boundary.**

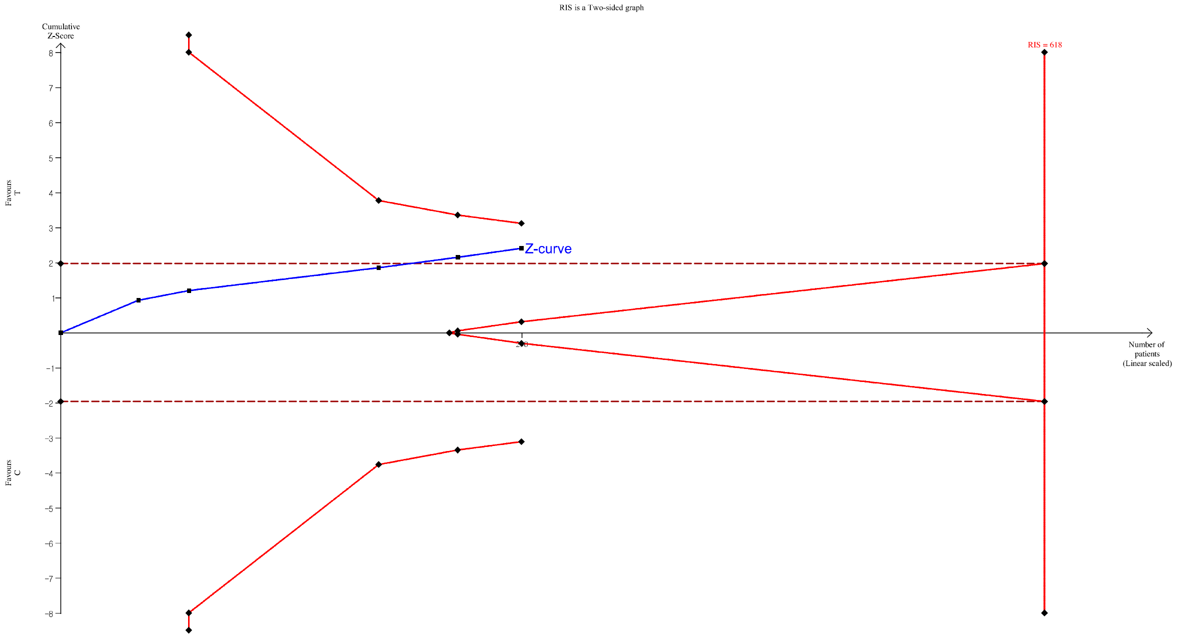

Supplementary Figure S37. Trial sequential analysis plot for post-operative nausea and vomiting comparing tropisetron and ondansetron. Uppermost and lowermost complete red curves represent trial sequential monitoring boundary lines for benefit and harm respectively. Horizontal dotted red line represents the conventional boundaries for statistical significance. Triangular red lines on the right side reflects the futility boundaries. The blue solid line represents the cumulative z-curve. The number on the x-axis indicates required information size. The TSA suggests insufficient evidence, with only 11.5 % of the required information size (RIS) accrued, as the Z-curve crossed neither the conventional test boundary nor cross the trial sequential monitoring boundary.

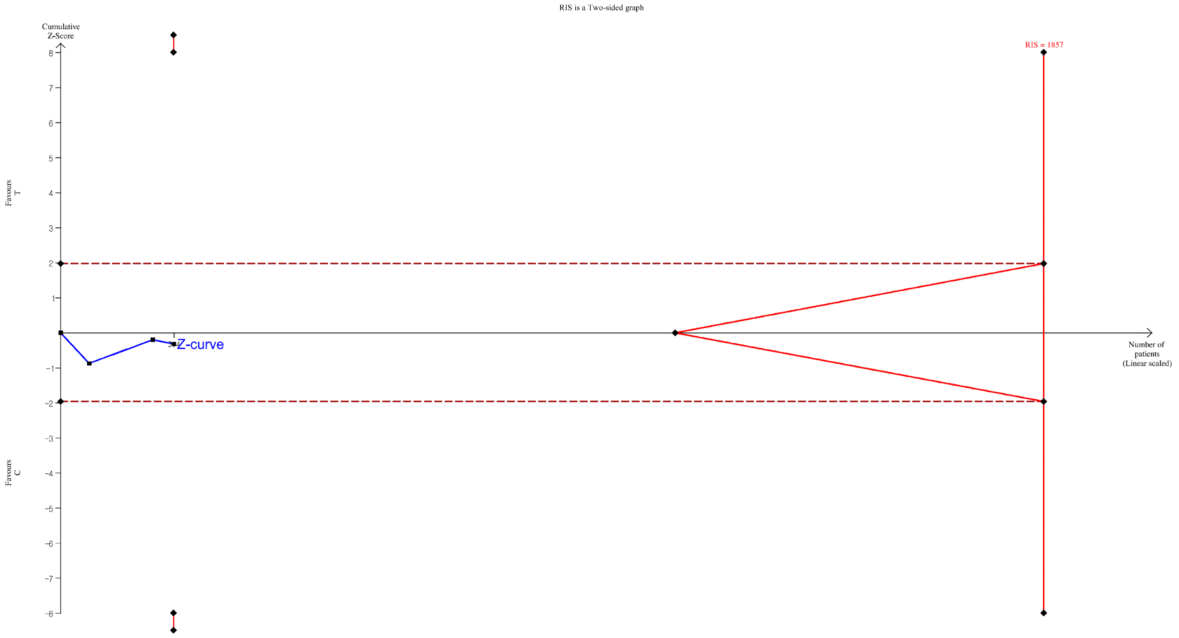

Supplementary Figure S38. Trial sequential analysis plot for post-operative nausea and vomiting comparing tropisetron and dexamethasone. Uppermost and lowermost complete red curves represent trial sequential monitoring boundary lines for benefit and harm respectively. Horizontal dotted red line represents the conventional boundaries for statistical significance. Triangular red lines on the right side reflects the futility boundaries. The blue solid line represents the cumulative z-curve. The number on the x-axis indicates required information size. The TSA suggests insufficient evidence, with only 6.1% of the required information size (RIS) accrued, as the Z-curve crossed neither the conventional test boundary nor cross the trial sequential monitoring boundary.

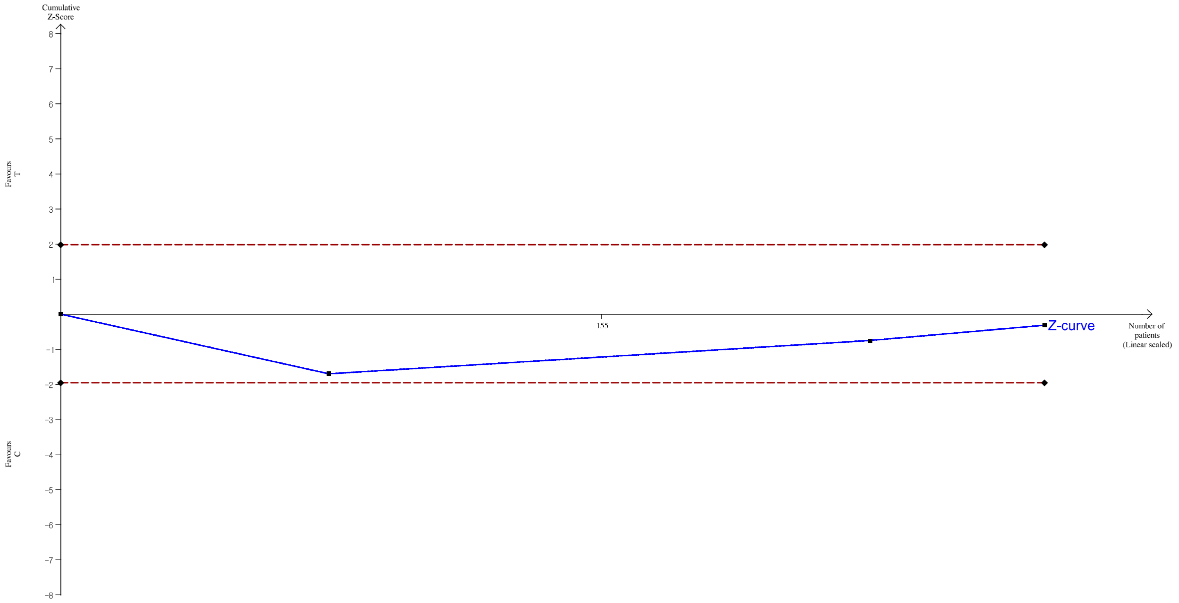

Supplementary Figure S39. Trial sequential analysis plot for post-operative nausea and vomiting comparing tropisetron and droperidol. Uppermost and lowermost complete red curves represent trial sequential monitoring boundary lines for benefit and harm respectively. Horizontal dotted red line represents the conventional boundaries for statistical significance. Triangular red lines on the right side reflects the futility boundaries. The blue solid line represents the cumulative z-curve. The number on the x-axis indicates required information size. The TSA suggests insufficient evidence, with only 25.9 % of the required information size (RIS) accrued, as the Z-curve crossed neither the conventional test boundary nor cross the trial sequential monitoring boundary.

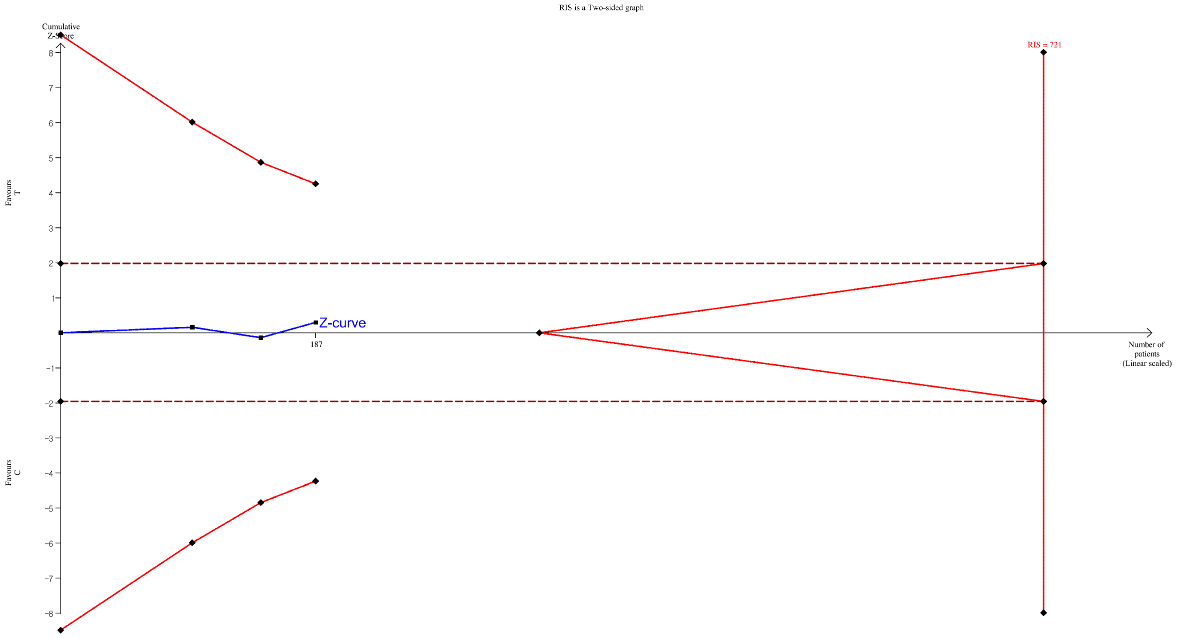

Supplementary Figure S40. Trial sequential analysis plot for post-operative nausea and vomiting comparing tropisetron and droperidol when including study comparing tropisetron combined with dexamethasone with droperidol combined with dexamethasone. Uppermost and lowermost complete red curves represent trial sequential monitoring boundary lines for benefit and harm respectively. Horizontal dotted red line represents the conventional boundaries for statistical significance. Triangular red lines on the right side reflects the futility boundaries. The blue solid line represents the cumulative z-curve. The number on the x-axis indicates required information size. The TSA suggests insufficient evidence, with only 36.1% of the required information size (RIS) accrued, as the Z-curve crossed neither the conventional test boundary nor cross the trial sequential monitoring boundary.

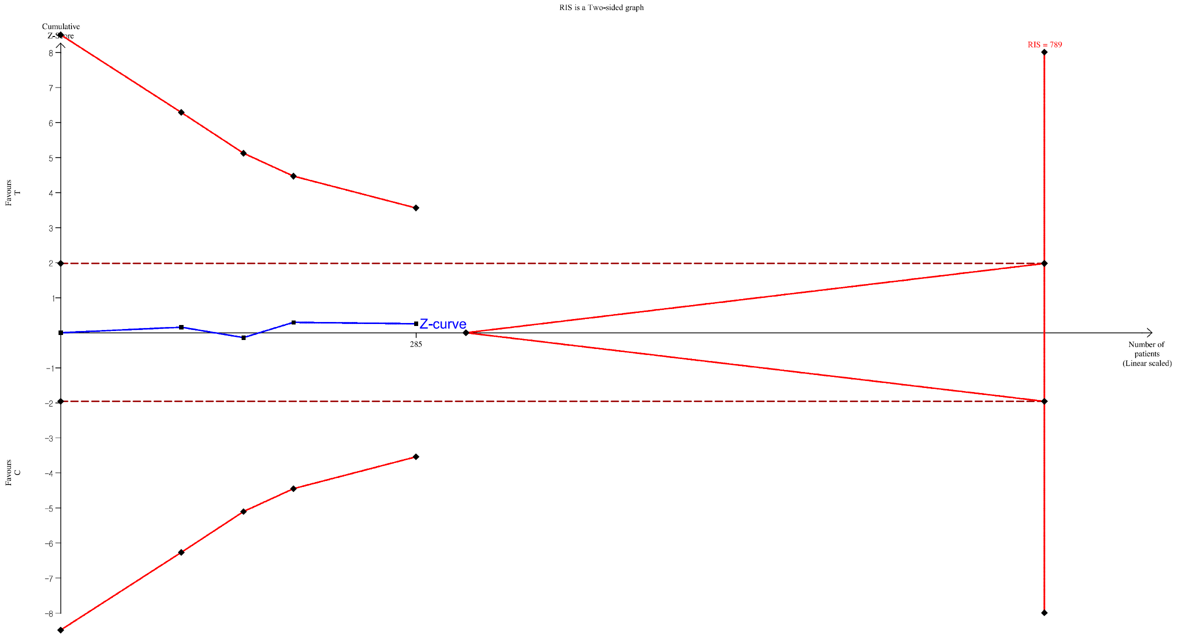

Supplementary Figure S41. Trial sequential analysis plot for post-operative nausea and vomiting comparing tropisetron and granisetron. Uppermost and lowermost complete red curves represent trial sequential monitoring boundary lines for benefit and harm respectively. Horizontal dotted red line represents the conventional boundaries for statistical significance. Triangular red lines on the right side reflects the futility boundaries. The blue solid line represents the cumulative z-curve. The number on the x-axis indicates required information size. The TSA suggests insufficient evidence, with only 12.7% of the required information size (RIS) accrued, as the Z-curve crossed neither the conventional test boundary nor cross the trial sequential monitoring boundary.

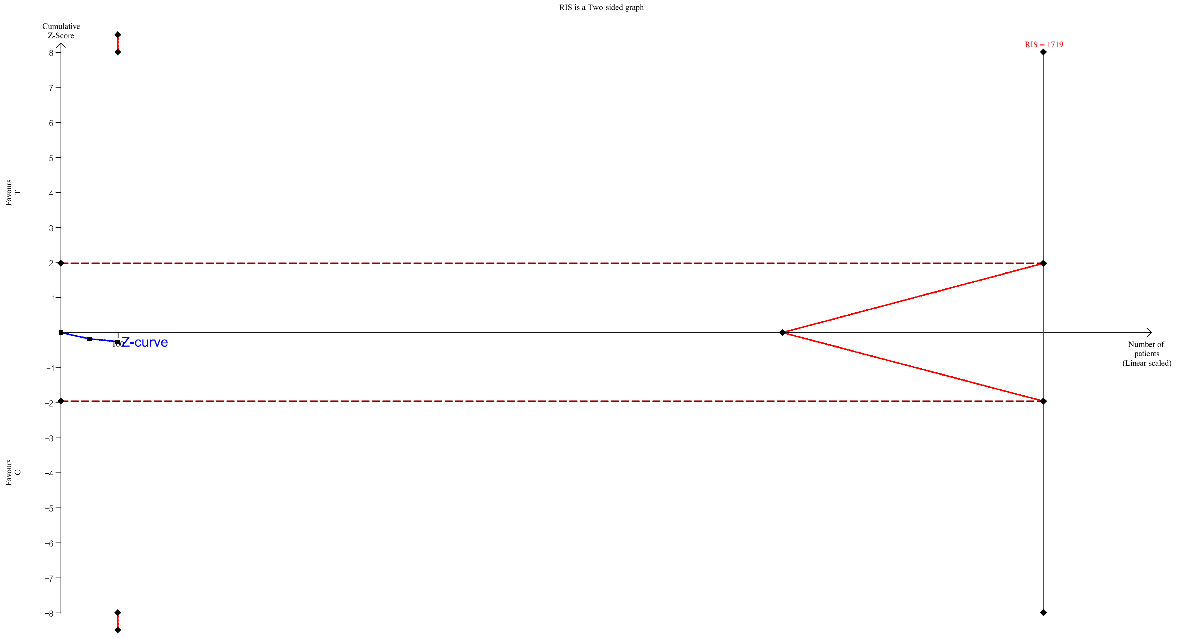

Supplementary Figure S42. Forest plot for use of rescue anti-emetics comparing tropisetron and control including studies comparing metoclopramide and tropisetron combined with metoclopramide and comparing dexamethasone and tropisetron combined with dexamethasone and comparing droperidol and tropisetron combined with droperidol. The figure depicts individual trials as filled squares with relative sample size and the 95% confidence interval (CI) of the difference as a solid line. The diamond shape indicates the pooled estimate and uncertainty for the combined effect. The pooled estimate indicates that the incidence of use of rescue anti-emetics is lower in tropisetron group than that in control group including studies comparing metoclopramide and tropisetron combined with metoclopramide and comparing dexamethasone and tropisetron combined with dexamethasone and comparing droperidol and tropisetron combined with droperidol.

Study name	Statistics for each study			Risk ratio and 95% CI
	Risk ratio	Lower limit	Upper limit	
Zomers, 1993	0.662	0.420	1.045	
Ali-Melkkila, 1996	0.636	0.275	1.474	
Capouet, 1996	0.745	0.548	1.011	
Naguib, 1996	0.718	0.463	1.113	
Jakobsson, 1996	0.909	0.446	1.853	
Purhonen, 1997	0.631	0.435	0.914	
Alon, 1998	0.500	0.164	1.528	
Chan,1998	0.510	0.384	0.678	
Scholz, 1998	0.750	0.569	0.989	
Tsui, 1999	0.384	0.171	0.862	
Muhammad, 2000	0.250	0.063	0.990	
Wang, 2002_1	0.385	0.169	0.878	
Wang, 2002_2	0.770	0.422	1.403	
Madenoglu, 2003	0.444	0.229	0.861	
Eberhart, 2006	0.475	0.285	0.792	
Ekinci, 2011	0.417	0.180	0.964	
Eberhart, 2006(2)	0.380	0.196	0.736	
Zhou, 2012	0.625	0.315	1.241	
Papadima, 2013	0.760	0.532	1.087	
Yang, 2015	0.539	0.168	1.730	
	0.624	0.560	0.696	

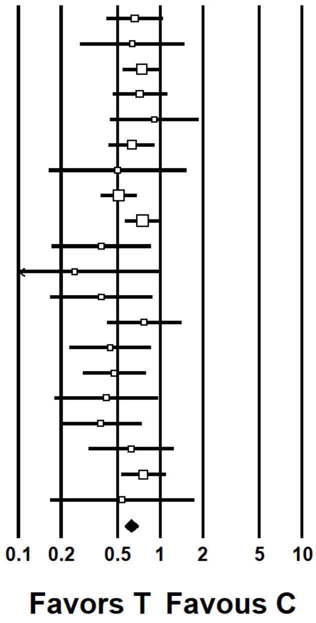

Supplementary Figure S43. Trial sequential analysis plot for use of rescue anti-emetics comparing tropisetron and control when including studies comparing metoclopramide and tropisetron combined with metoclopramide, dexamethasone and tropisetron combined with dexamethasone, droperidol and tropisetron combined with droperidol. Uppermost and lowermost complete red curves represent trial sequential monitoring boundary lines for benefit and harm respectively. Horizontal dotted red line represents the conventional boundaries for statistical significance. Triangular red lines on the right side reflects the futility boundaries. The blue solid line represents the cumulative z-curve. The number on the x-axis indicates required information size (992). The cumulative Z-curve crosses the alpha spending boundary, favoring tropisetron over control for use of rescue anti-emetics when including studies comparing metoclopramide and tropisetron combined with metoclopramide, dexamethasone and tropisetron combined with dexamethasone, droperidol and tropisetron combined with droperidol.

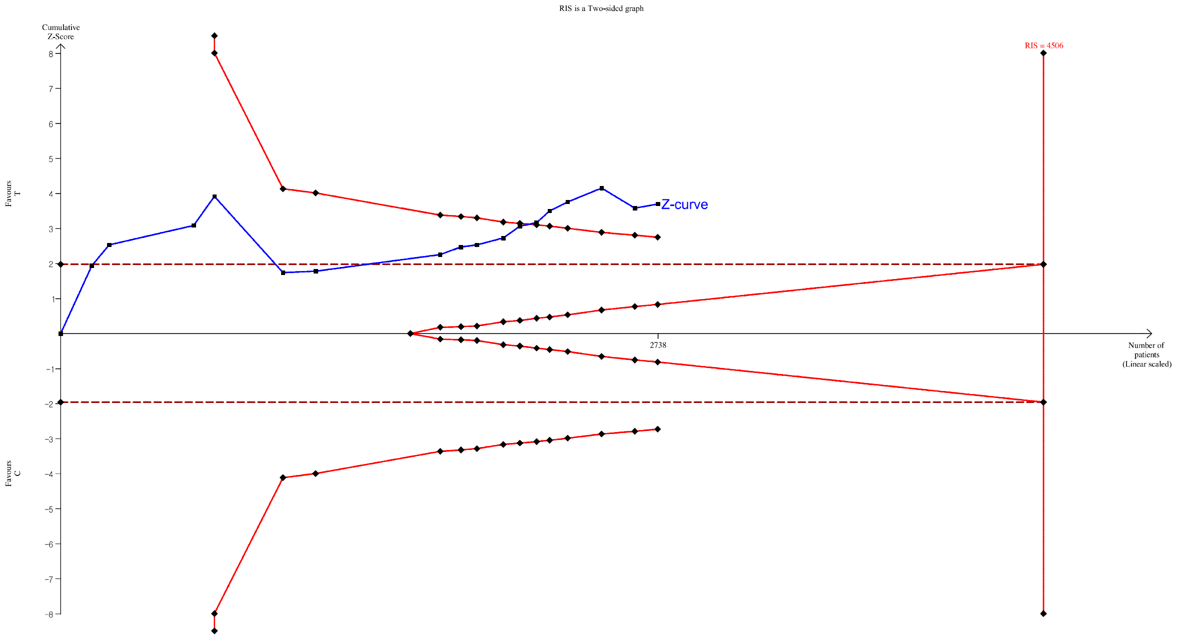

Supplementary Figure S44. Forest plot for use of rescue anti-emetics comparing tropisetron and metoclopramide. The figure depicts individual trials as filled squares with relative sample size and the 95% confidence interval (CI) of the difference as a solid line. The diamond shape indicates the pooled estimate and uncertainty for the combined effect. The pooled estimate indicates significant difference in the incidence of use of rescue anti-emetics between tropisetron and metoclopramide.

Study name	Statistics for each study		
	Risk ratio	Lower limit	Upper limit
Ali-Melkkila, 1996	1.167	0.430	3.166
Naguib, 1996	0.734	0.465	1.158
Muhammad, 2000	0.286	0.070	1.171
Jokela, 2002	0.702	0.520	0.949
Ekinci, 2011	0.625	0.247	1.584
	0.707	0.561	0.892

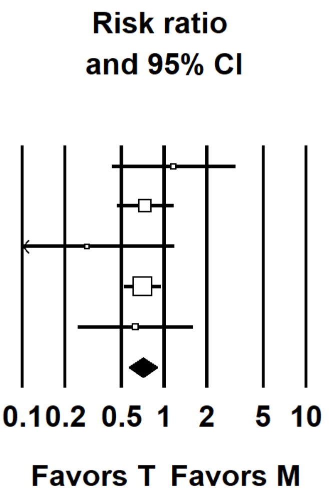

Supplementary Figure S45. Forest plot for use of rescue anti-emetics comparing tropisetron and ondansetron. The figure depicts individual trials as filled squares with relative sample size and the 95% confidence interval (CI) of the difference as a solid line. The diamond shape indicates the pooled estimate and uncertainty for the combined effect. The pooled estimate indicates no significant difference in the incidence of use of rescue anti-emetics between tropisetron and ondansetron.

Study name	Statistics for each study		
	Risk ratio	Lower limit	Upper limit
Naguib, 1996	1.508	0.805	2.824
Scholz, 1998	1.095	0.800	1.500
Tsui, 1999	0.632	0.255	1.566
Jokela, 2002	0.789	0.574	1.085
Argiriadou, 2002	0.668	0.239	1.871
Ekinci, 2011	1.667	0.459	6.056
	0.968	0.759	1.235

Risk ratio and 95% CI

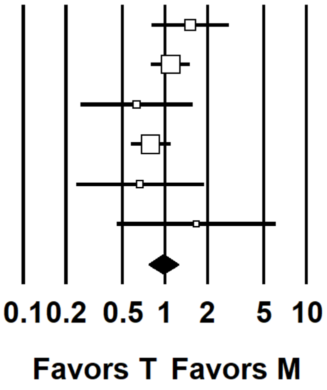

Supplementary Figure S46. Forest plot for use of rescue anti-emetics comparing tropisetron and ondansetron when including study comparing tropisetron combined with droperidol and ondansetron combined with droperidol. The figure depicts individual trials as filled squares with relative sample size and the 95% confidence interval (CI) of the difference as a solid line. The diamond shape indicates the pooled estimate and uncertainty for the combined effect. The pooled estimate indicates no significant difference in the incidence of use of rescue anti-emetics between tropisetron and ondansetron when including study comparing tropisetron combined with droperidol and ondansetron combined with droperidol.

Study name	Statistics for each study		
	Risk ratio	Lower limit	Upper limit
Naguib, 1996	1.508	0.805	2.824
Scholz, 1998	1.095	0.800	1.500
Tsui, 1999	0.632	0.255	1.566
Jokela, 2002	0.789	0.574	1.085
Argiriadou, 2002	0.668	0.239	1.871
Ekinci, 2011	1.667	0.459	6.056
Koivuranta, 1999	1.271	0.718	2.249
	1.000	0.806	1.242

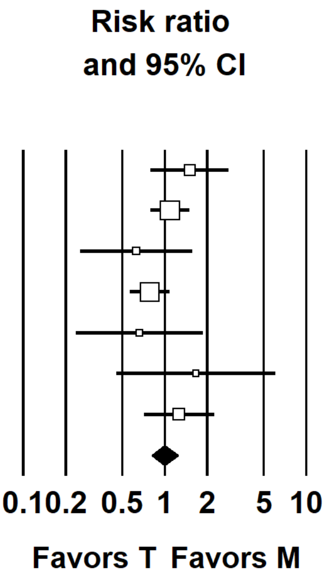

Supplementary Figure S47. Forest plot for use of rescue anti-emetics comparing tropisetron and dexamethasone. The figure depicts individual trials as filled squares with relative sample size and the 95% confidence interval (CI) of the difference as a solid line. The diamond shape indicates the pooled estimate and uncertainty for the combined effect. The pooled estimate indicates no significant difference in the incidence of use of rescue anti-emetics between tropisetron and dexamethasone.

Study name	Statistics for each study			Risk ratio and 95% CI
	Risk ratio	Lower limit	Upper limit	
Wang, 2002_1	1.200	0.400	3.599	
Wang, 2002_2	2.053	0.858	4.910	
Eberhart, 2006	0.600	0.349	1.032	
Zhou, 2012	0.938	0.522	1.683	
	0.988	0.594	1.645	

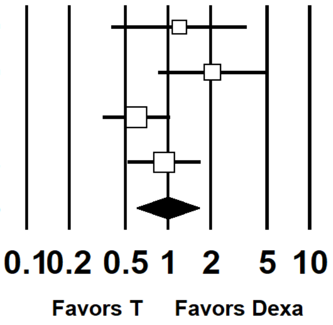

Supplementary Figure S48. Forest plot for use of rescue anti-emetics comparing tropisetron and droperidol. The figure depicts individual trials as filled squares with relative sample size and the 95% confidence interval (CI) of the difference as a solid line. The diamond shape indicates the pooled estimate and uncertainty for the combined effect. The pooled estimate indicates no significant difference in the incidence of use of rescue anti-emetics between tropisetron and droperidol.

Study name	Statistics for each study		
	Risk ratio	Lower limit	Upper limit
Jokela, 1999	0.833	0.563	1.233
Purhonen, 1997	0.858	0.562	1.308
Ekinci, 2011	1.250	0.392	3.986
	0.864	0.654	1.142

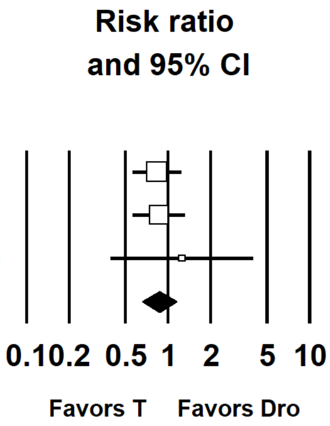

Supplementary Figure S49. Forest plot for use of rescue anti-emetics comparing tropisetron and granisetron. The figure depicts individual trials as filled squares with relative sample size and the 95% confidence interval (CI) of the difference as a solid line. The diamond shape indicates the pooled estimate and uncertainty for the combined effect. The pooled estimate indicates no significant difference in the incidence of use of rescue anti-emetics between tropisetron and granisetron.

Study name	Statistics for each study		
	Risk ratio	Lower limit	Upper limit
Naguib,1996	1.083	0.622	1.888
	1.083	0.622	1.888

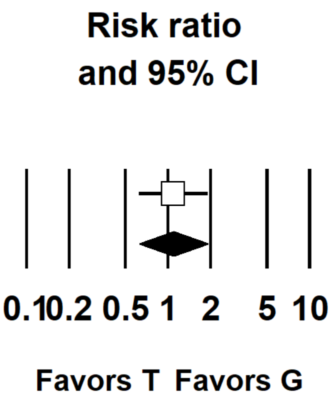

Supplementary Figure S50. Forest plot for use of rescue anti-emetics comparing tropisetron and granisetron including study comparing tropisetron combined with droperidol and granisetron combined with droperidol. The figure depicts individual trials as filled squares with relative sample size and the 95% confidence interval (CI) of the difference as a solid line. The diamond shape indicates the pooled estimate and uncertainty for the combined effect. The pooled estimate indicates no significant difference in the incidence of use of rescue anti-emetics between tropisetron and granisetron including study comparing tropisetron combined with droperidol and granisetron combined with droperidol.

Study name	Statistics for each study		
	Risk ratio	Lower limit	Upper limit
Naguib,1996	1.083	0.622	1.888
Papadima, 2013	1.390	0.863	2.239
	1.250	0.871	1.795

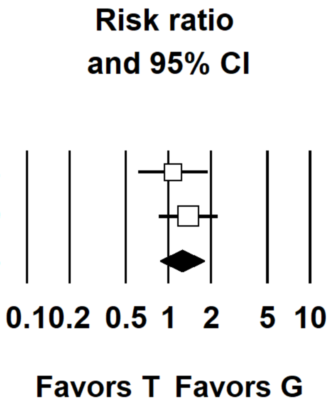

Supplementary Figure S51. Trial sequential analysis plot for use of rescue anti-emetics comparing tropisetron and metoclopramide. Uppermost and lowermost complete red curves represent trial sequential monitoring boundary lines for benefit and harm respectively. Horizontal dotted red line represents the conventional boundaries for statistical significance. Triangular red lines on the right side reflects the futility boundaries. The blue solid line represents the cumulative z-curve. The number on the x-axis indicates required information size. The TSA suggests **insufficient evidence**, with only 41.6 % of the required information size (RIS) accrued, as the Z-curve crossed the conventional test boundary but did not cross the trial sequential monitoring boundary.

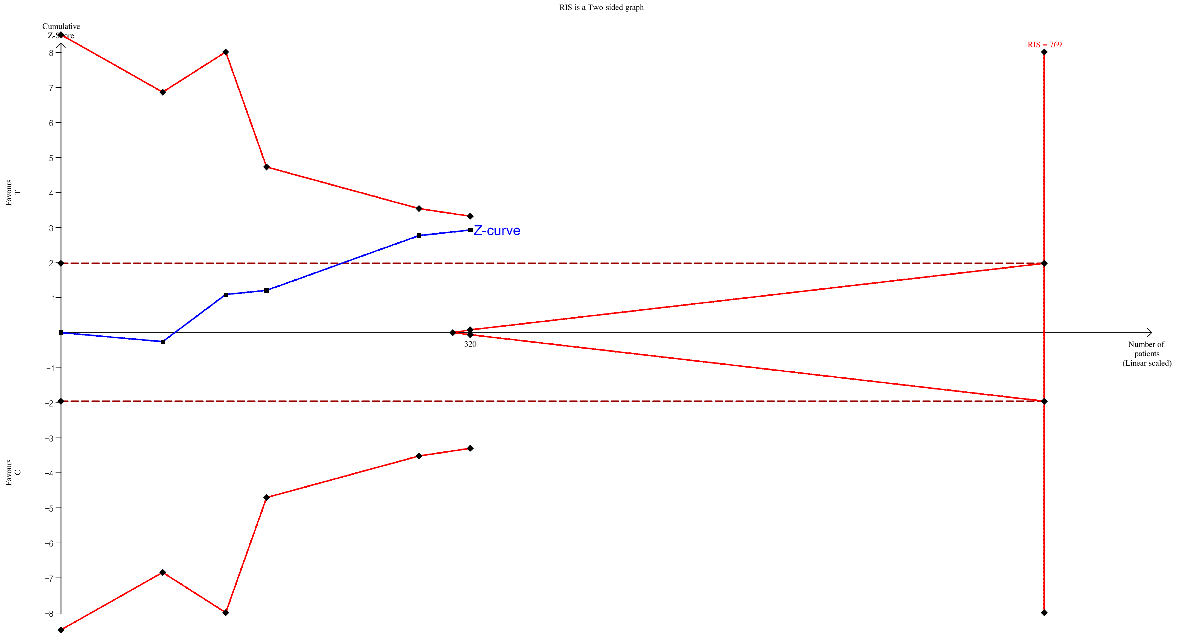

Supplementary Figure S52. Trial sequential analysis plot for use of rescue anti-emetics comparing tropisetron and ondansetron. Uppermost and lowermost complete red curves represent trial sequential monitoring boundary lines for benefit and harm respectively. Horizontal dotted red line represents the conventional boundaries for statistical significance. Triangular red lines on the right side reflects the futility boundaries. The blue solid line represents the cumulative z-curve. The number on the x-axis indicates required information size. The TSA suggests insufficient evidence, with only 31.4 % of the required information size (RIS) accrued, as the Z-curve crossed neither the conventional test boundary nor cross the trial sequential monitoring boundary.

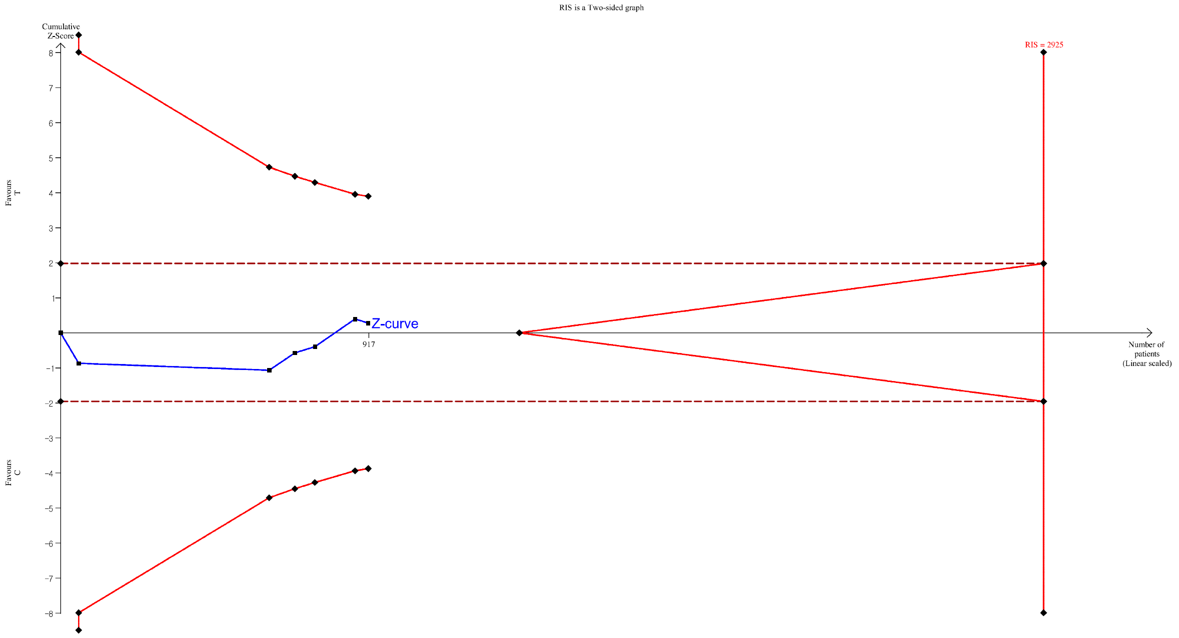

Supplementary Figure S53. Trial sequential analysis plot for use of rescue anti-emetics comparing tropisetron and ondansetron including study comparing tropisetron combined with droperidol and ondansetron combined with droperidol. Uppermost and lowermost complete red curves represent trial sequential monitoring boundary lines for benefit and harm respectively. Horizontal dotted red line represents the conventional boundaries for statistical significance. Triangular red lines on the right side reflects the futility boundaries. The blue solid line represents the cumulative z-curve. The number on the x-axis indicates required information size. The TSA suggests insufficient evidence, with only 34.4 % of the required information size (RIS) accrued, as the Z-curve crossed neither the conventional test boundary nor cross the trial sequential monitoring boundary.

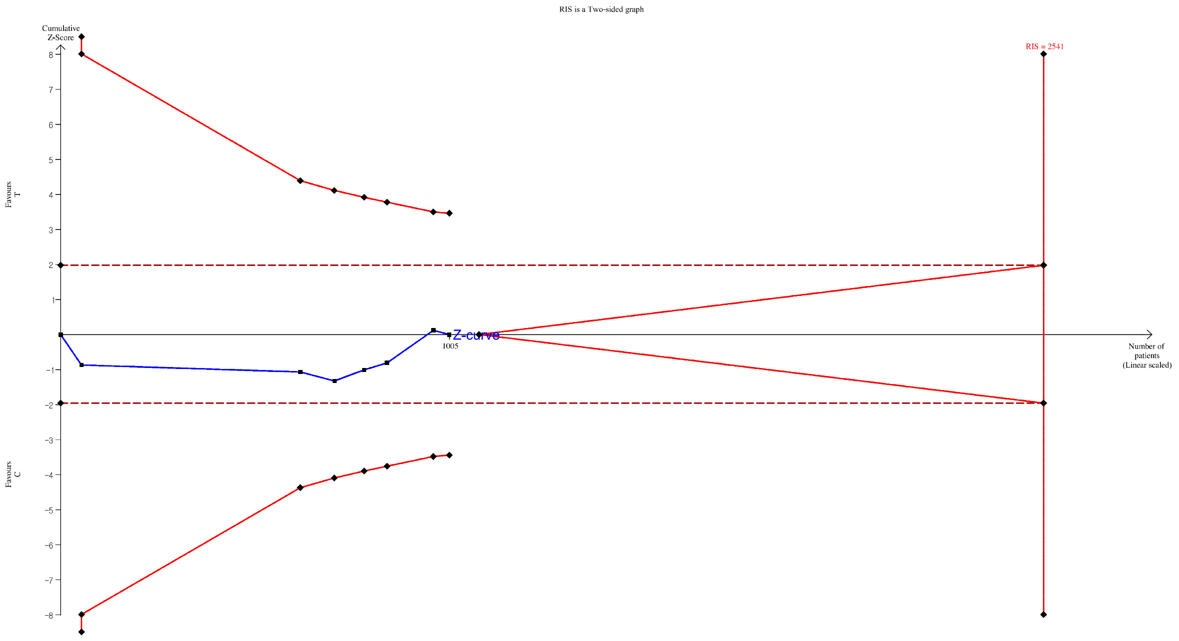

Supplementary Figure S54. Trial sequential analysis plot for use of rescue anti-emetics comparing tropisetron and dexamethasone. Uppermost and lowermost complete red curves represent trial sequential monitoring boundary lines for benefit and harm respectively. Horizontal dotted red line represents the conventional boundaries for statistical significance. Triangular red lines on the right side reflects the futility boundaries. The blue solid line represents the cumulative z-curve. The number on the x-axis indicates required information size. The TSA suggests insufficient evidence, with only 16.1% of the required information size (RIS) accrued, as the Z-curve crossed neither the conventional test boundary nor cross the trial sequential monitoring boundary.

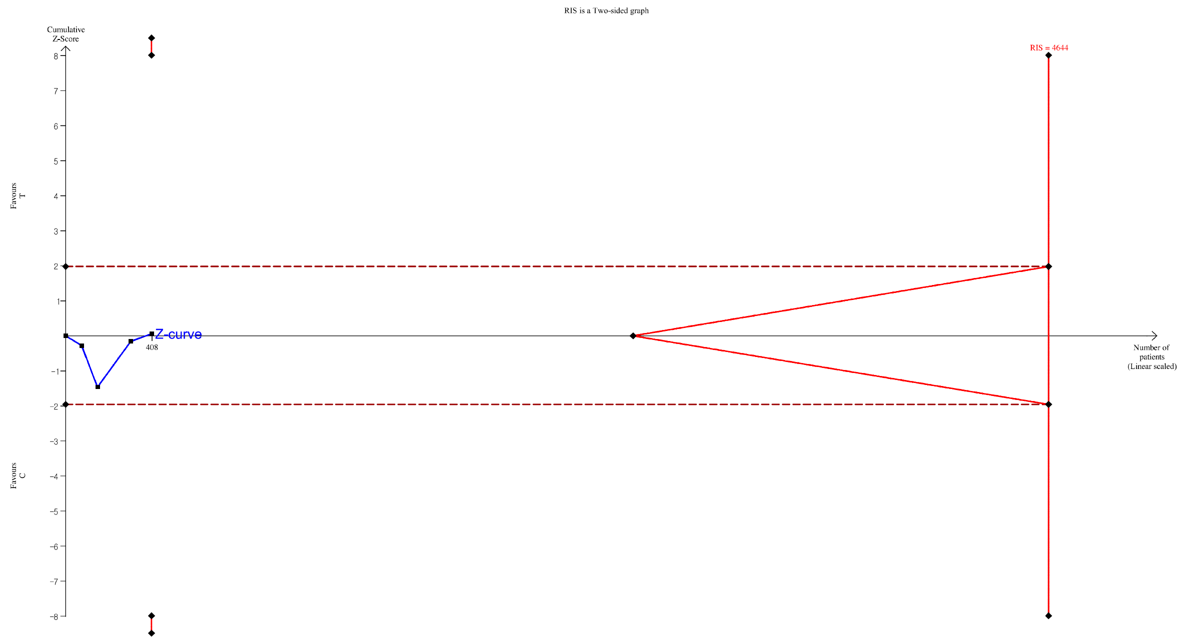

Supplementary Figure S55. Trial sequential analysis plot for use of rescue anti-emetics comparing tropisetron and droperidol. Uppermost and lowermost complete red curves represent trial sequential monitoring boundary lines for benefit and harm respectively. Horizontal dotted red line represents the conventional boundaries for statistical significance. Triangular red lines on the right side reflects the futility boundaries. The blue solid line represents the cumulative z-curve. The number on the x-axis indicates required information size. The TSA suggests insufficient evidence, with only 28.2 % of the required information size (RIS) accrued, as the Z-curve crossed neither the conventional test boundary nor cross the trial sequential monitoring boundary.

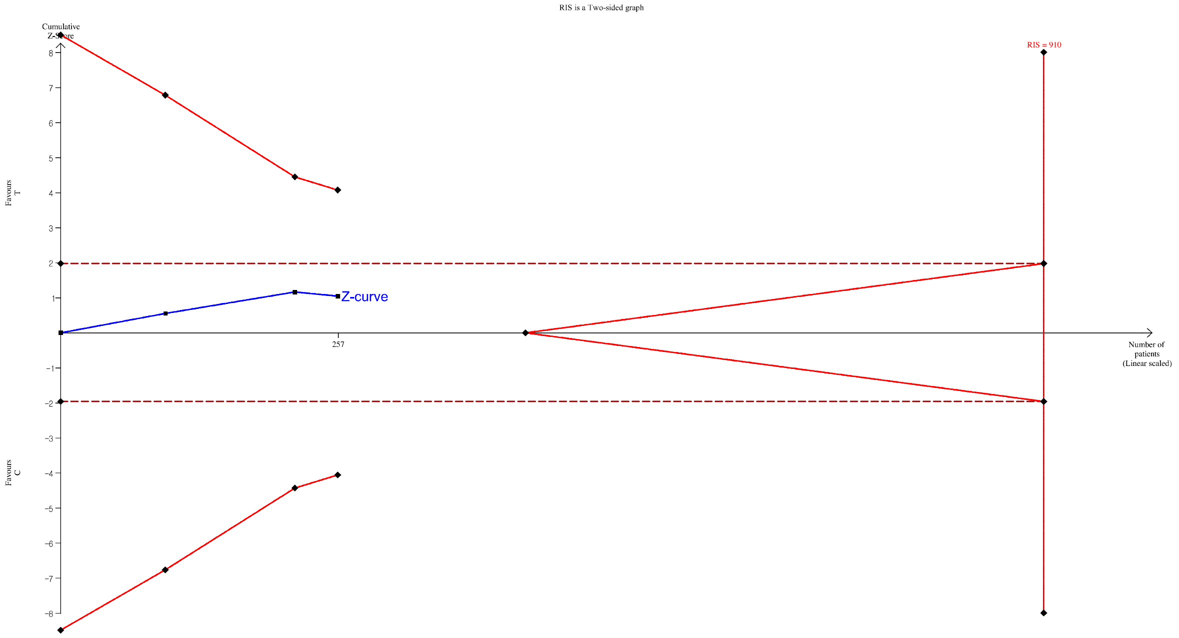

Supplementary Figure S56. Trial sequential analysis plot for use of rescue anti-emetics comparing tropisetron and granisetron including study comparing tropisetron combined with droperidol and granisetron combined with droperidol. Uppermost and lowermost complete red curves represent trial sequential monitoring boundary lines for benefit and harm respectively. Horizontal dotted red line represents the conventional boundaries for statistical significance. Triangular red lines on the right side reflects the futility boundaries. The blue solid line represents the cumulative z-curve. The number on the x-axis indicates required information size. The TSA suggests insufficient evidence, with only 14.8 % of the required information size (RIS) accrued, as the Z-curve crossed neither the conventional test boundary nor cross the trial sequential monitoring boundary.

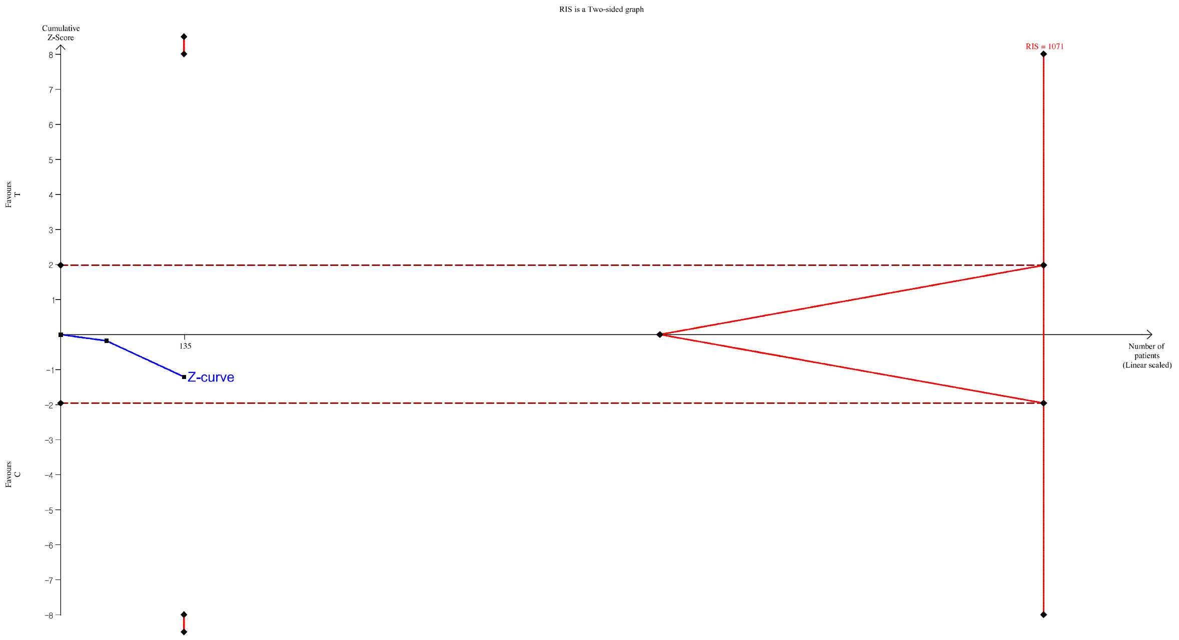

Supplementary Figure S57. Forest plot for complete response comparing tropisetron and control including studies comparing dexamethasone and tropisetron combined with dexamethasone. The figure depicts individual trials as filled squares with relative sample size and the 95% confidence interval (CI) of the difference as a solid line. The diamond shape indicates the pooled estimate and uncertainty for the combined effect. The pooled estimate indicates that the incidence of complete response is higher in tropisetron group than that in control group including studies comparing dexamethasone and tropisetron combined with dexamethasone.

Study name	Risk ratio	Lower limit	Upper limit
Ali-Melkkila, 1996	1.526	1.046	2.227
Alon, 1996	1.625	1.044	2.529
Purhonen, 1997	1.361	0.632	2.931
Chan,1998	3.253	1.676	6.311
Jakobsson, 1999	0.857	0.567	1.295
Wang, 2002_1	1.979	1.242	3.154
Wang, 2002_2	1.246	0.720	2.157
Eberhart, 2006	1.532	1.113	2.109
Ekinci, 2011	1.571	0.767	3.218
Eberhart, 2006(2)	1.287	1.033	1.604
Zhou, 2012	1.345	1.018	1.776
Yi, 2022	1.518	0.964	2.390
	1.442	1.250	1.663

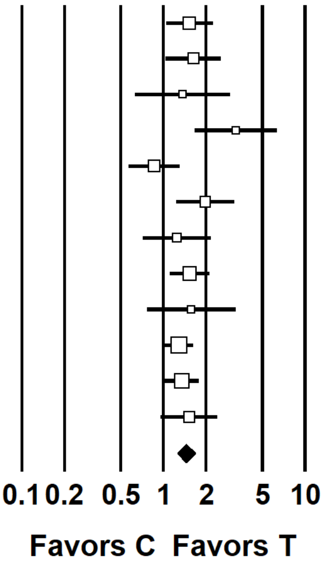

Supplementary Figure S58. Trial sequential analysis plot for complete response comparing tropisetron and control including studies comparing dexamethasone and tropisetron combined with dexamethasone. Uppermost and lowermost complete red curves represent trial sequential monitoring boundary lines for benefit and harm respectively. Horizontal dotted red line represents the conventional boundaries for statistical significance. Triangular red lines on the right side reflects the futility boundaries. The blue solid line represents the cumulative z-curve. The number on the x-axis indicates required information size (3947). The cumulative Z-curve crosses the alpha spending boundary, favoring tropisetron over control for complete response when including studies comparing dexamethasone and tropisetron combined with dexamethasone.

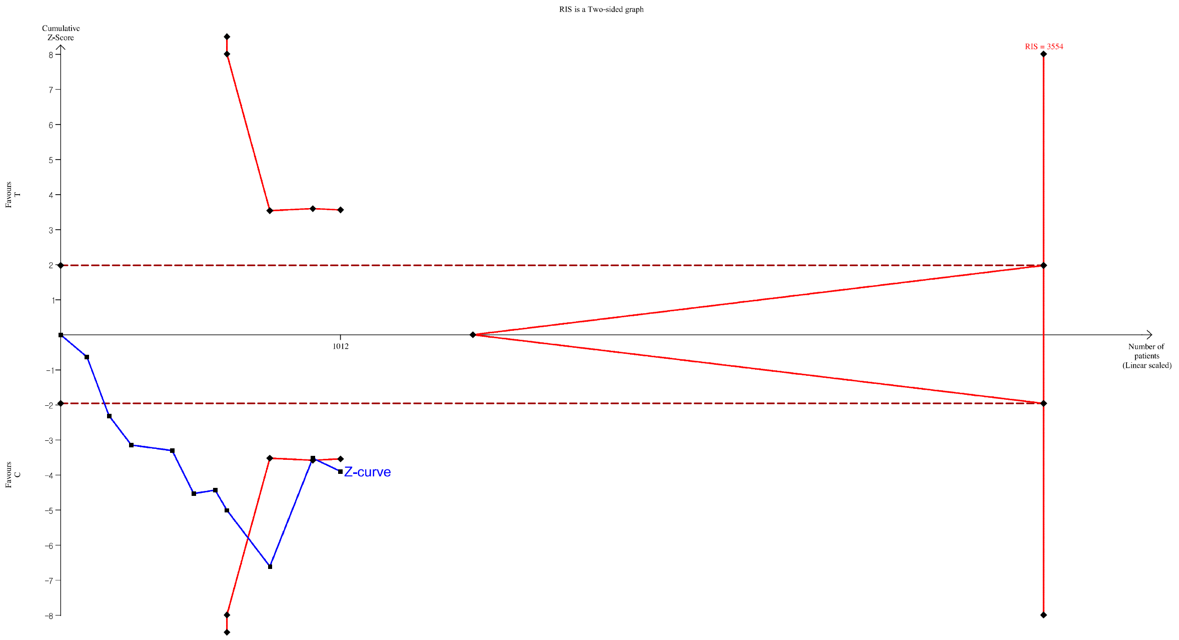

Supplementary Figure S59. Forest plot for complete response comparing tropisetron and metoclopramide. The figure depicts individual trials as filled squares with relative sample size and the 95% confidence interval (CI) of the difference as a solid line. The diamond shape indicates the pooled estimate and uncertainty for the combined effect. The pooled estimate indicates no significant difference in the incidence of complete response between tropisetron and metoclopramide.

Study name	Statistics for each study			Risk ratio and 95% CI
	Risk ratio	Lower limit	Upper limit	
Ali-Melkkila, 1996	1.115	0.829	1.501	
Jokela, 2002	1.639	0.965	2.784	
Ekinci, 2011	1.000	0.571	1.752	
	1.180	0.933	1.493	

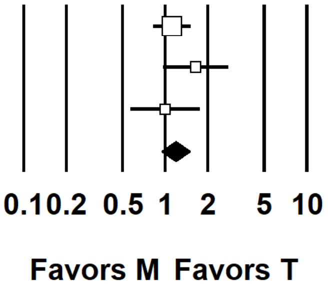

Supplementary Figure S60. Forest plot for complete response comparing tropisetron and ondansetron. The figure depicts individual trials as filled squares with relative sample size and the 95% confidence interval (CI) of the difference as a solid line. The diamond shape indicates the pooled estimate and uncertainty for the combined effect. The pooled estimate indicates no significant difference in the incidence of complete response between tropisetron and ondansetron.

Study name	Statistics for each study		
	Risk ratio	Lower limit	Upper limit
Naguib, 1996	0.633	0.401	0.999
Jokela, 2002	1.316	0.816	2.121
Quan, 2007	0.981	0.828	1.161
Ekinci, 2011	0.846	0.508	1.410
	0.928	0.726	1.185

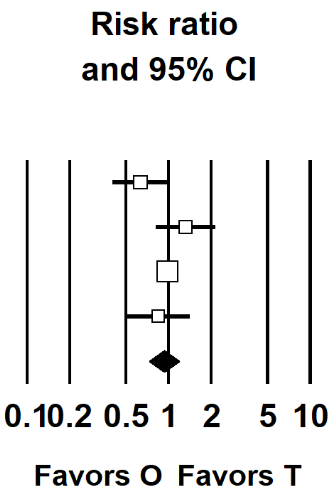

Supplementary Figure S61. Forest plot for complete response comparing tropisetron and dexamethasone. The figure depicts individual trials as filled squares with relative sample size and the 95% confidence interval (CI) of the difference as a solid line. The diamond shape indicates the pooled estimate and uncertainty for the combined effect. The pooled estimate indicates no significant difference in the incidence of complete response between tropisetron and dexamethasone.

Study name	Statistics for each study		
	Risk ratio	Lower limit	Upper limit
Wang, 2002_1	0.964	0.730	1.273
Wang, 2002_2	0.602	0.404	0.896
Eberhart, 2006	1.042	0.811	1.339
Zhou, 2012	1.138	0.836	1.549
	0.939	0.747	1.181

Risk ratio and 95% CI

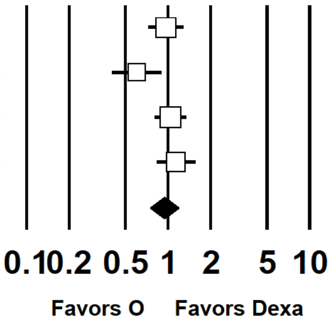

Supplementary Figure S62. Forest plot for complete response comparing tropisetron and droperidol. The figure depicts individual trials as filled squares with relative sample size and the 95% confidence interval (CI) of the difference as a solid line. The diamond shape indicates the pooled estimate and uncertainty for the combined effect. The pooled estimate indicates no significant difference in the incidence of complete response between tropisetron and droperidol.

Study name	Statistics for each study		
	Risk ratio	Lower limit	Upper limit
Purhonen, 1997	1.114	0.545	2.276
Jokela, 1999	1.174	0.767	1.797
Ekinci, 2011	1.100	0.609	1.986
	1.142	0.836	1.558

Risk ratio and 95% CI

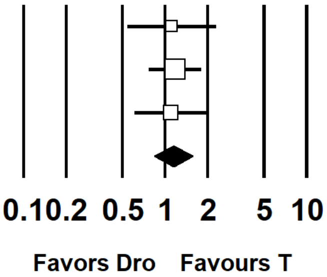

Supplementary Figure S63. Forest plot for complete response comparing tropisetron and droperidol including study comparing tropisetron combined with dexamethasone with droperidol combined with dexamethasone. The figure depicts individual trials as filled squares with relative sample size and the 95% confidence interval (CI) of the difference as a solid line. The diamond shape indicates the pooled estimate and uncertainty for the combined effect. The pooled estimate indicates no significant difference in the incidence of complete response between tropisetron and droperidol including study comparing tropisetron combined with dexamethasone with droperidol combined with dexamethasone.

Study name	Statistics for each study		
	Risk ratio	Lower limit	Upper limit
Purhonen, 1997	1.114	0.545	2.276
Jokela, 1999	1.174	0.767	1.797
Ekinci, 2011	1.100	0.609	1.986
yi, 2002	0.992	0.694	1.419
	1.075	0.850	1.359

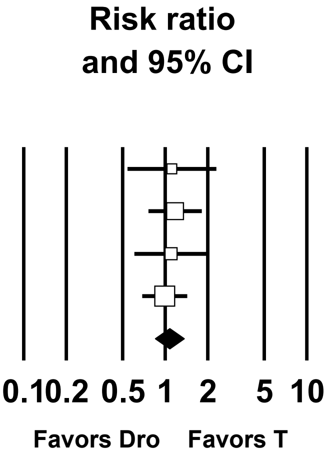

Supplementary Figure S64. Forest plot for complete response comparing tropisetron and granisetron. The figure depicts individual trials as filled squares with relative sample size and the 95% confidence interval (CI) of the difference as a solid line. The diamond shape indicates the pooled estimate and uncertainty for the combined effect. The pooled estimate indicates no significant difference in the incidence of complete response between tropisetron and granisetron.

Study name	Statistics for each study		
	Risk ratio	Lower limit	Upper limit
Naguib, 1996	0.857	0.502	1.465
Quan, 2007	0.942	0.799	1.110
	0.934	0.798	1.093

Risk ratio and 95% CI

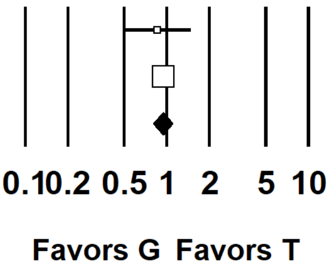

Supplementary Figure S65. Trial sequential analysis plot for complete response comparing tropisetron and metoclopramide. Horizontal dotted red line represents the conventional boundaries for statistical significance. The blue solid line represents the cumulative z-curve. The number on the x-axis indicates required information size. The TSA suggests insufficient evidence, with only 21.9 % of the required information size (RIS) accrued, as the Z-curve but crossed neither the conventional test boundary nor cross the trial sequential monitoring boundary.

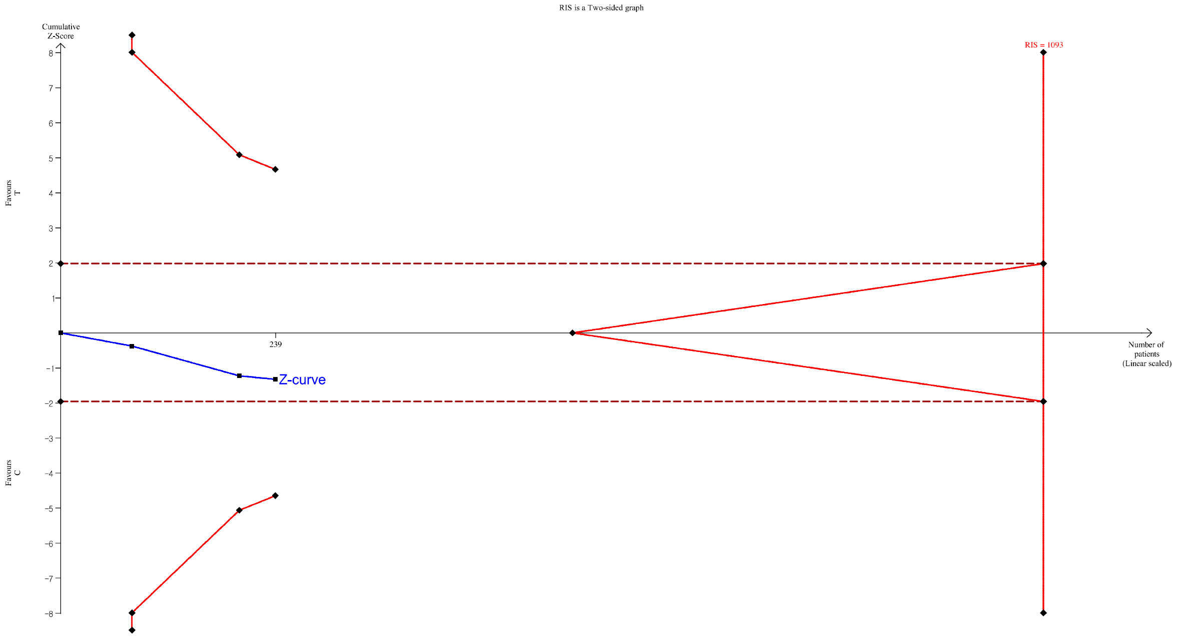

Supplementary Figure S66. Trial sequential analysis plot for complete response comparing tropisetron and ondansetron. Horizontal dotted red line represents the conventional boundaries for statistical significance. The blue solid line represents the cumulative z-curve. The number on the x-axis indicates required information size. The TSA suggests insufficient evidence, with only 29.1 % of the required information size (RIS) accrued, as the Z-curve but crossed neither the conventional test boundary nor cross the trial sequential monitoring boundary.

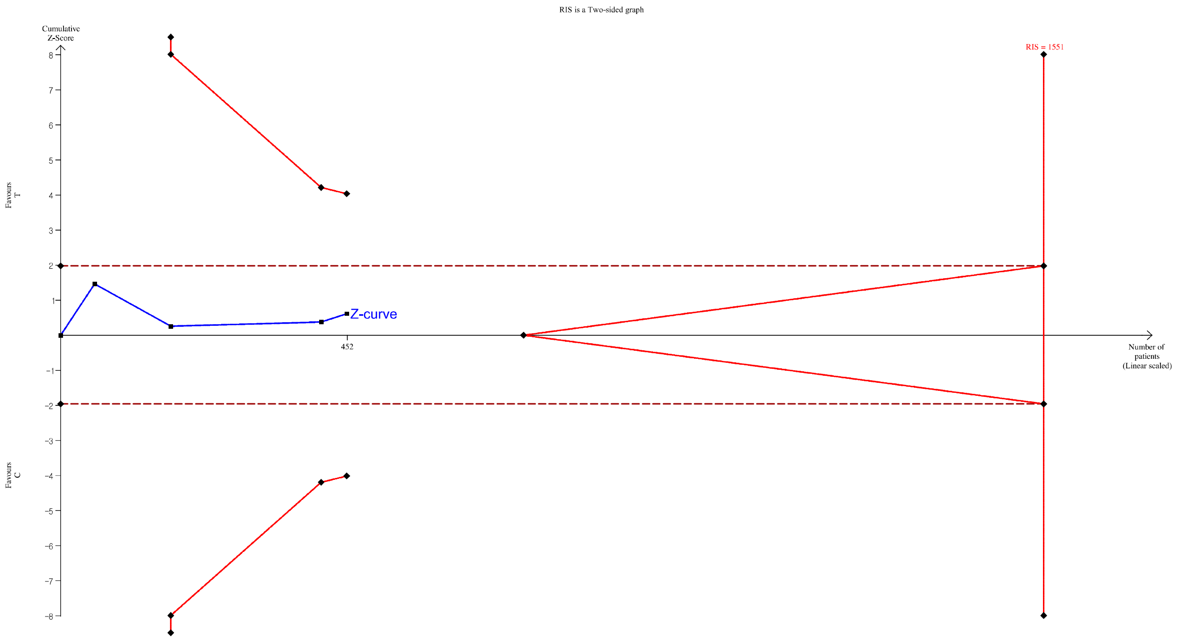

Supplementary Figure S67. Trial sequential analysis plot for complete response comparing tropisetron and dexamethasone. Horizontal dotted red line represents the conventional boundaries for statistical significance. The blue solid line represents the cumulative z-curve. The number on the x-axis indicates required information size. The TSA suggests insufficient evidence, with only 42.0 % of the required information size (RIS) accrued, as the Z-curve but crossed neither the conventional test boundary nor cross the trial sequential monitoring boundary.

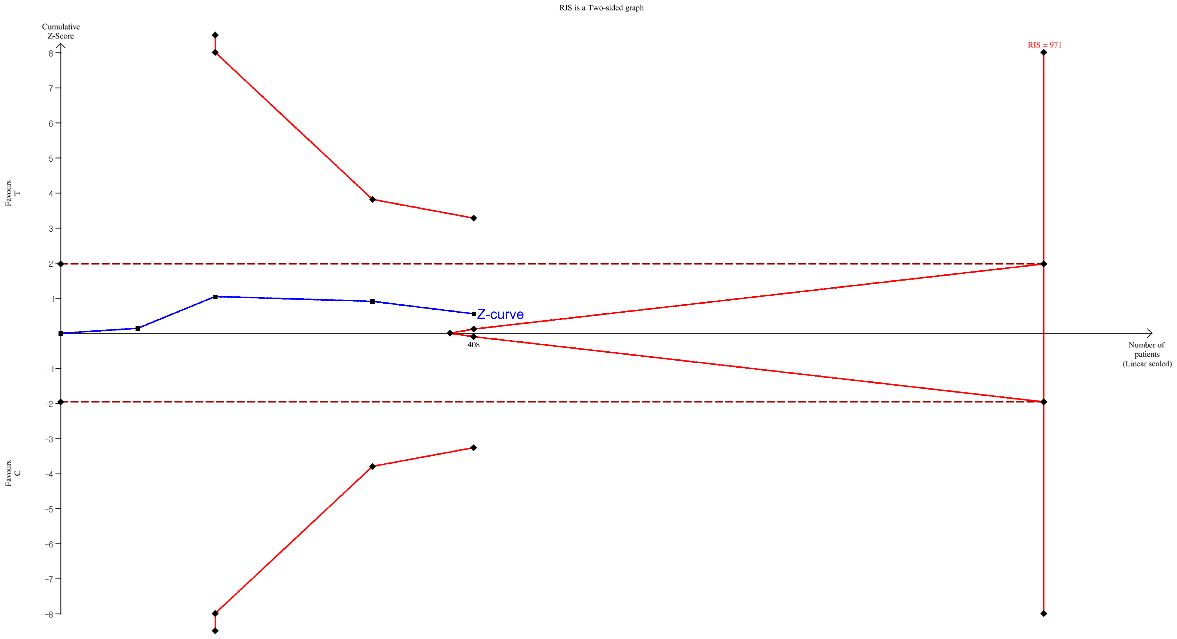

Supplementary Figure S68. Trial sequential analysis plot for complete response comparing tropisetron and droperidol. Horizontal dotted red line represents the conventional boundaries for statistical significance. The blue solid line represents the cumulative z-curve. The number on the x-axis indicates required information size. The TSA suggests insufficient evidence, with only 17.9% of the required information size (RIS) accrued, as the Z-curve but crossed neither the conventional test boundary nor cross the trial sequential monitoring boundary.

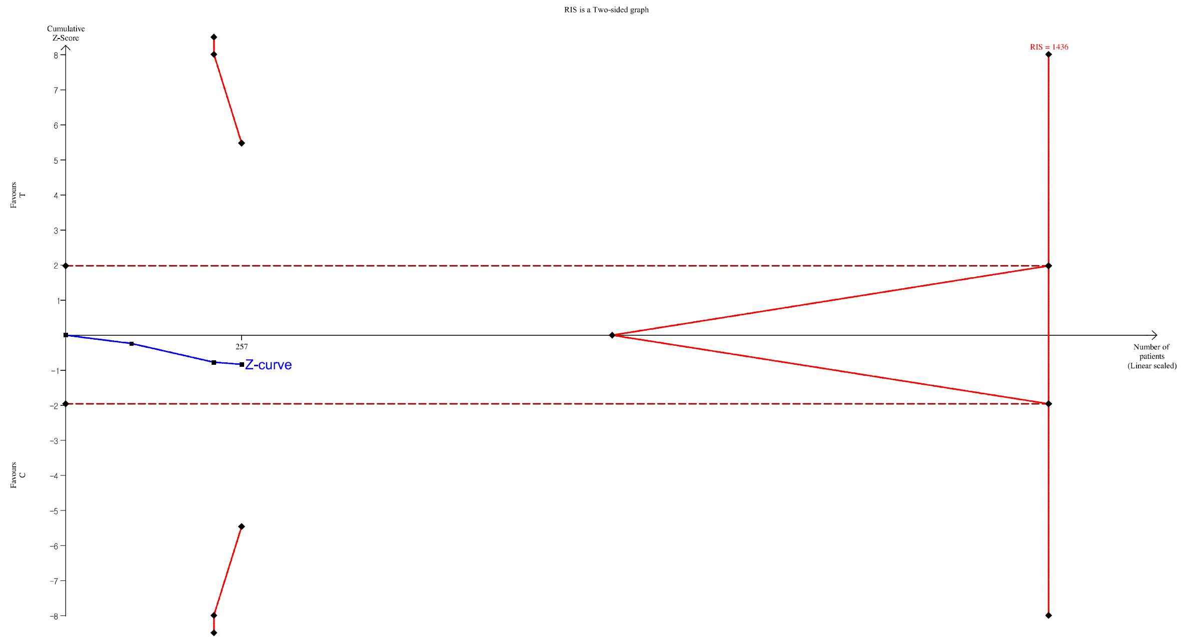

Supplementary Figure S69. Trial sequential analysis plot for complete response comparing tropisetron and droperidol when including study comparing tropisetron combined with dexamethasone with droperidol combined with dexamethasone. Horizontal dotted red line represents the conventional boundaries for statistical significance. The blue solid line represents the cumulative z-curve. The number on the x-axis indicates required information size. The TSA suggests insufficient evidence, with only 24.7 % of the required information size (RIS) accrued, as the Z-curve but crossed neither the conventional test boundary nor cross the trial sequential monitoring boundary.

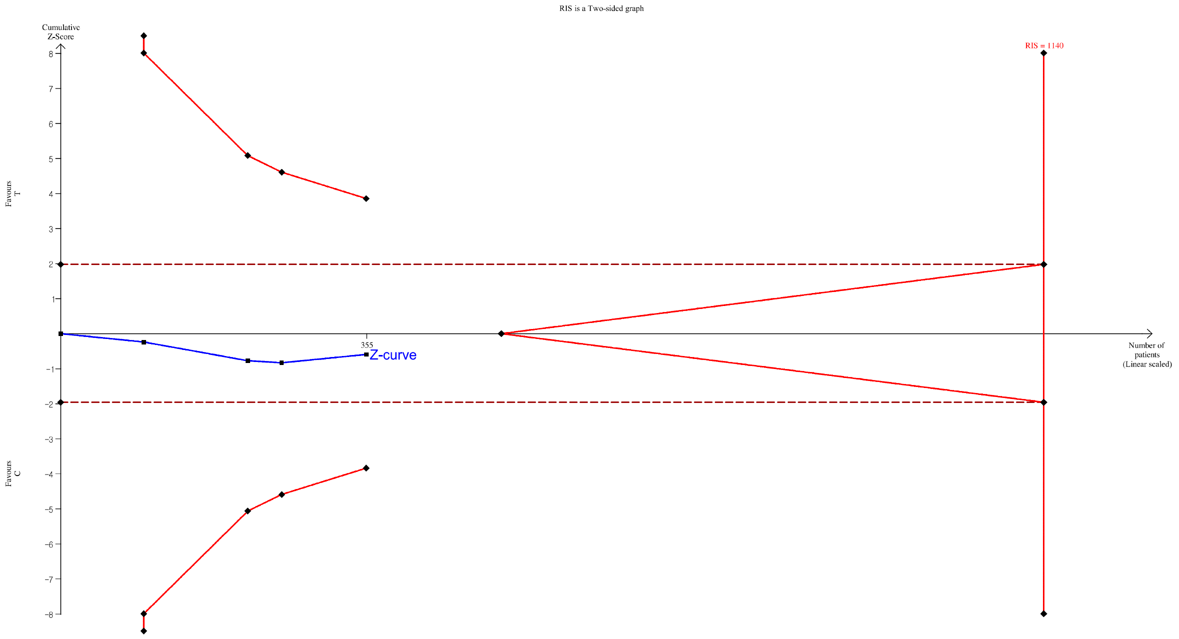

Supplementary Figure S70. Trial sequential analysis plot for complete response comparing tropisetron and granisetron. Horizontal dotted red line represents the conventional boundaries for statistical significance. The blue solid line represents the cumulative z-curve. The number on the x-axis indicates required information size. The TSA suggests insufficient evidence, with only 39.3% of the required information size (RIS) accrued, as the Z-curve but crossed neither the conventional test boundary nor cross the trial sequential monitoring boundary.

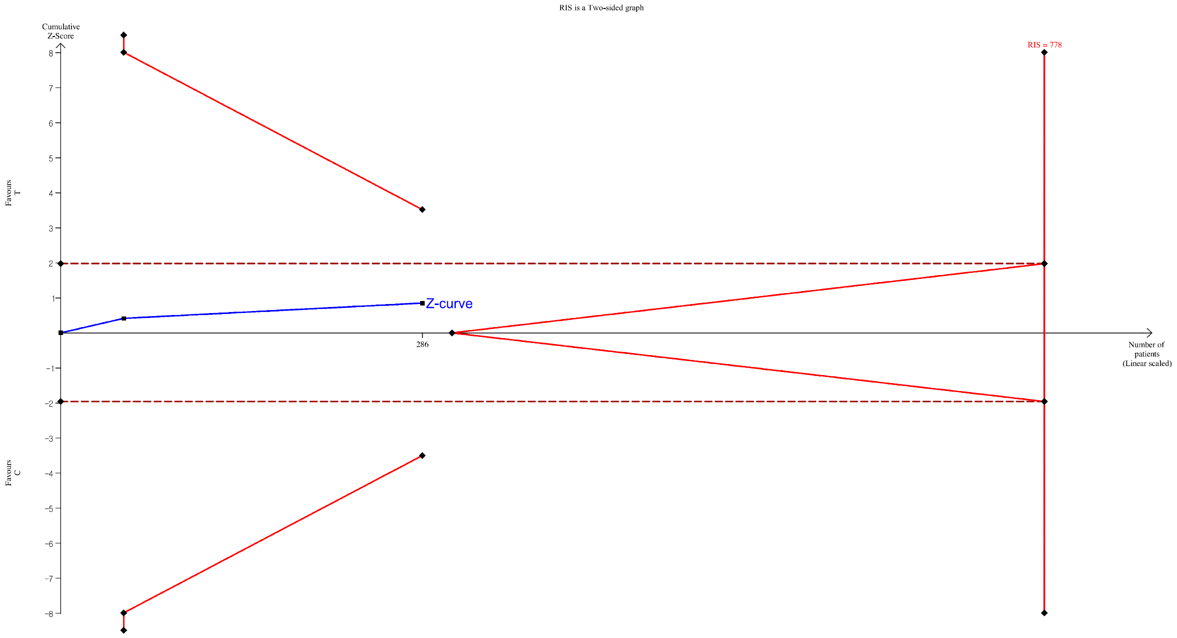

Supplementary Figure S71. Funnel plot for postoperative nausea compared with control

The horizontal axis demonstrates the log of risk ratio, and the vertical axis shows the inverse of the standard error.

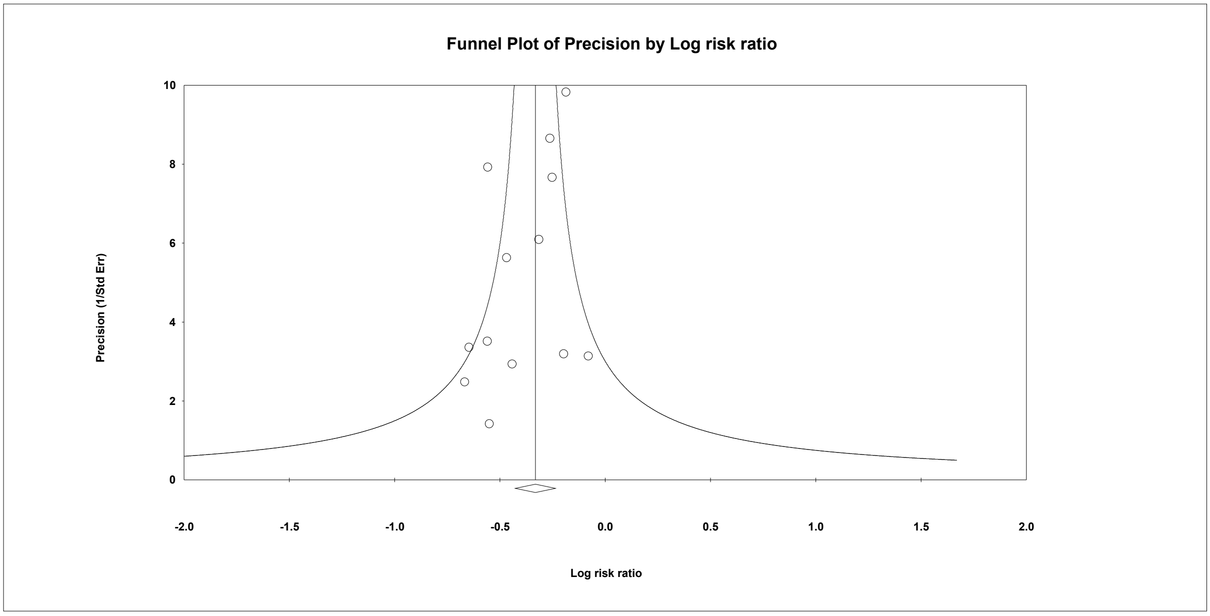

Supplementary Figure S72. Funnel plot for postoperative nausea compared with control including studies comparing dexamethasone and tropisetron combined with dexamethasone. The horizontal axis demonstrates the log of risk ratio, and the vertical axis shows the inverse of the standard error.

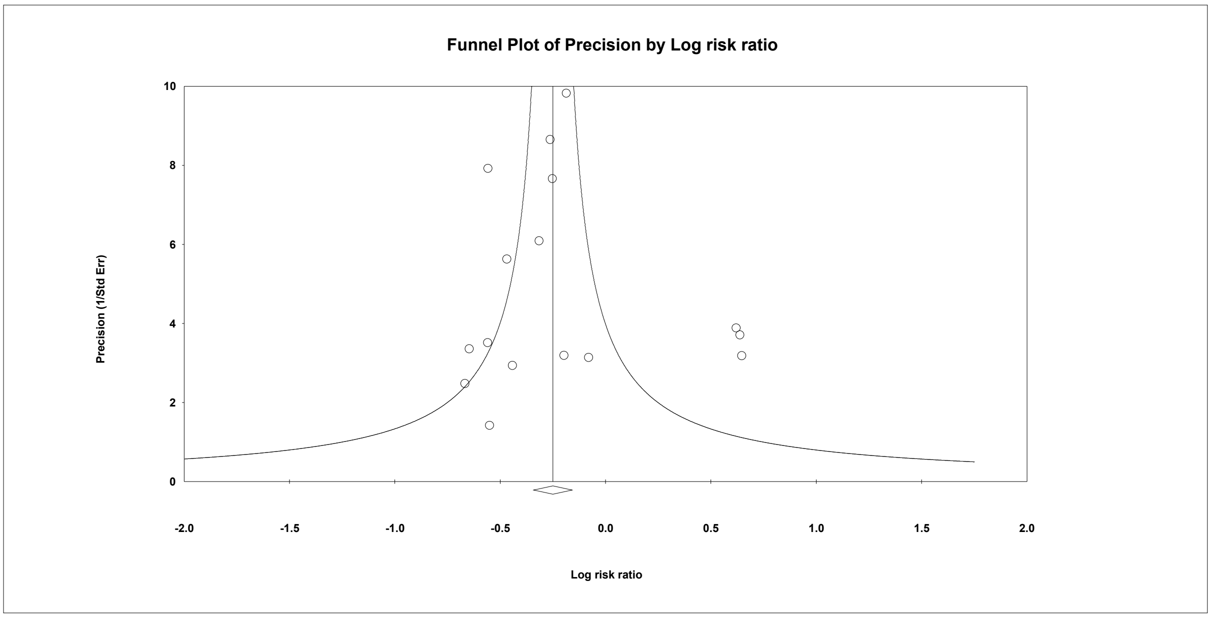

Supplementary Figure S73. Funnel plot for postoperative nausea compared with control including studies comparing dexamethasone and tropisetron combined with dexamethasone. The horizontal axis demonstrates the log of risk ratio, and the vertical axis shows the inverse of the standard error.

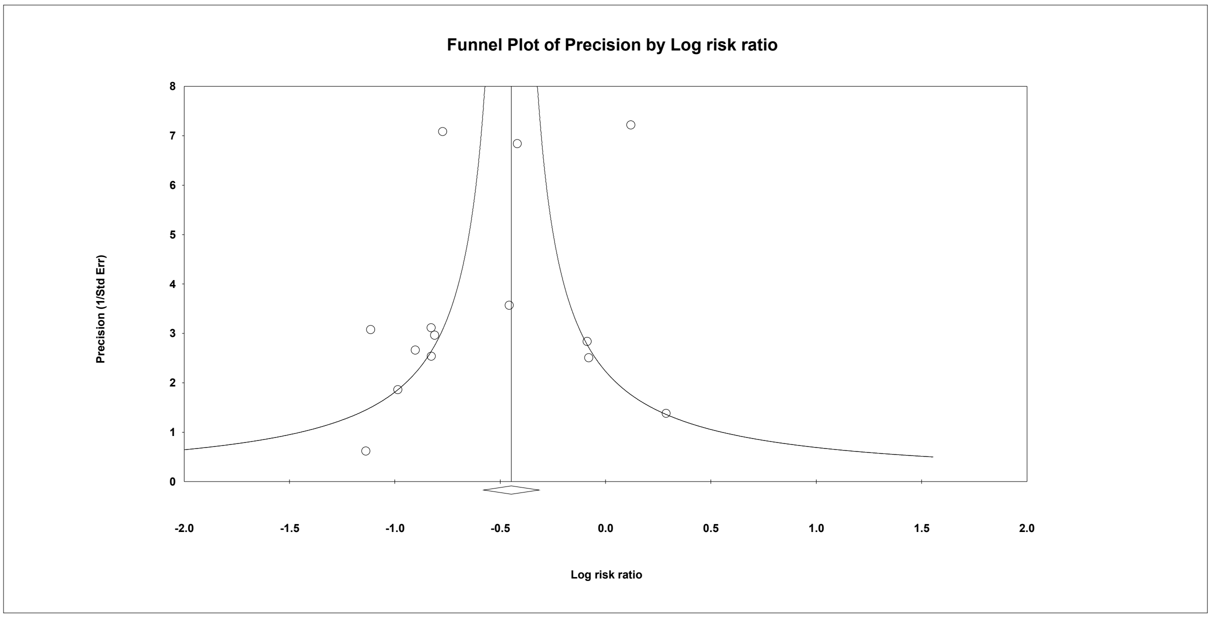

Supplementary Figure S74. Funnel plot for postoperative vomiting compared with control
including studies comparing droperidol and tropisetron combined with droperidol and comparing dexamethasone and tropisetron combined with dexamethasone. The horizontal axis demonstrates the log of risk ratio, and the vertical axis shows the inverse of the standard error.

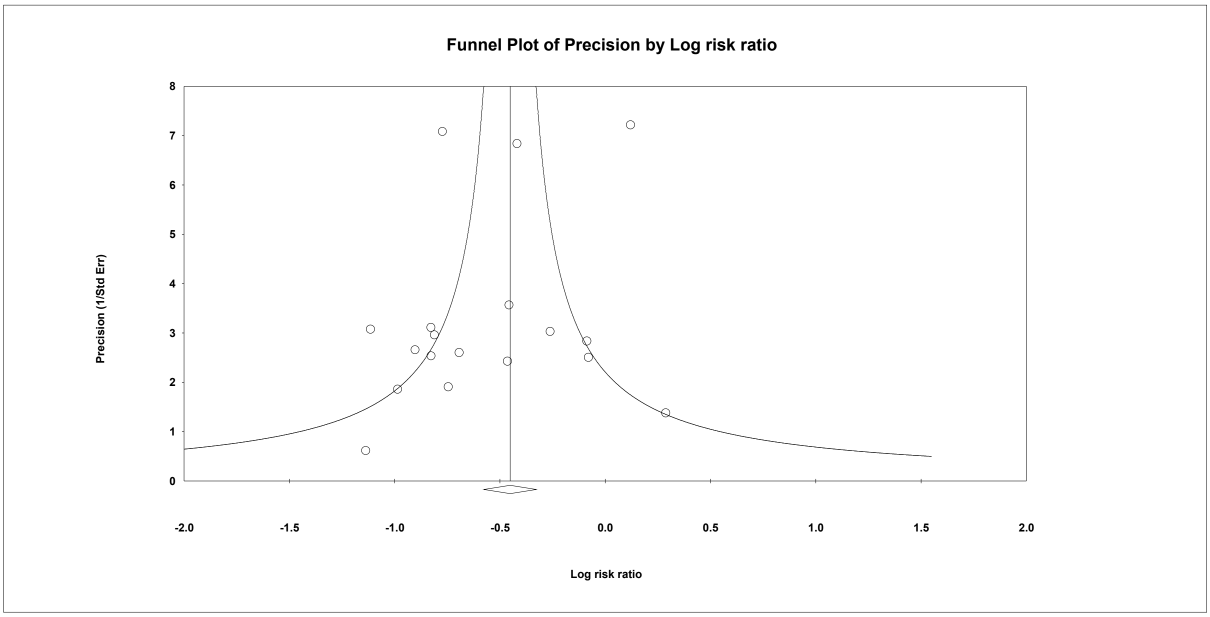

Supplementary Figure S75. Funnel plot for complete response compared with control including studies comparing dexamethasone and tropisetron combined with dexamethasone. The horizontal axis demonstrates the log of risk ratio, and the vertical axis shows the inverse of the standard error.

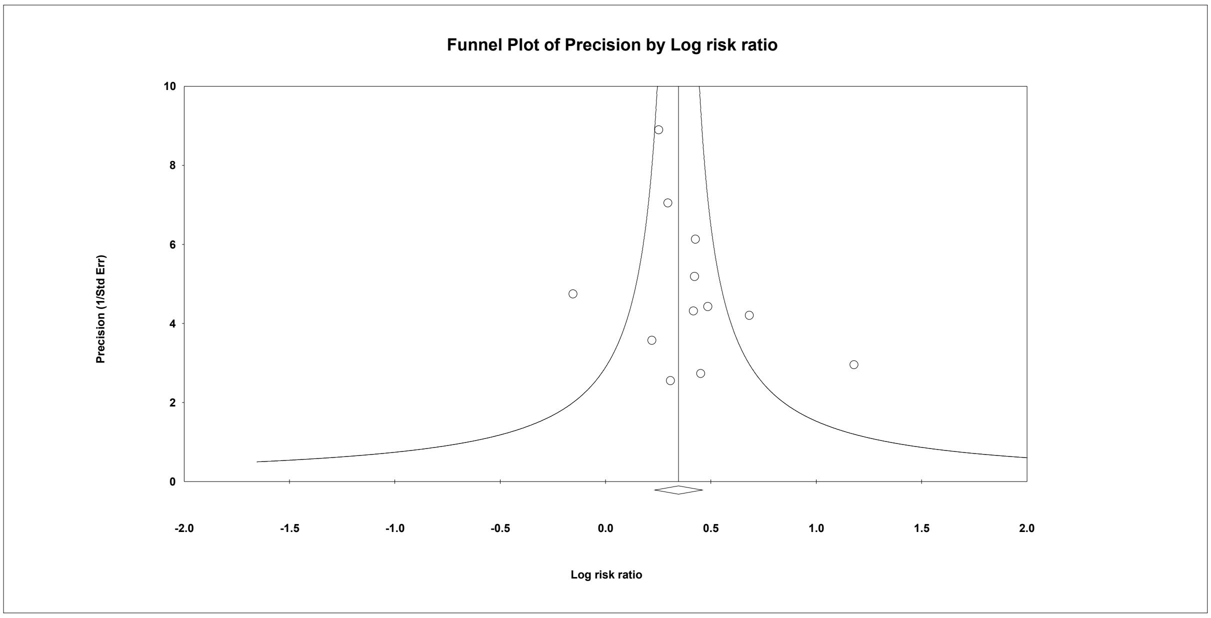

Supplementary Figure S76. Funnel plot for post-operative nausea and vomiting compared with control. The horizontal axis demonstrates the log of risk ratio, and the vertical axis shows the inverse of the standard error.

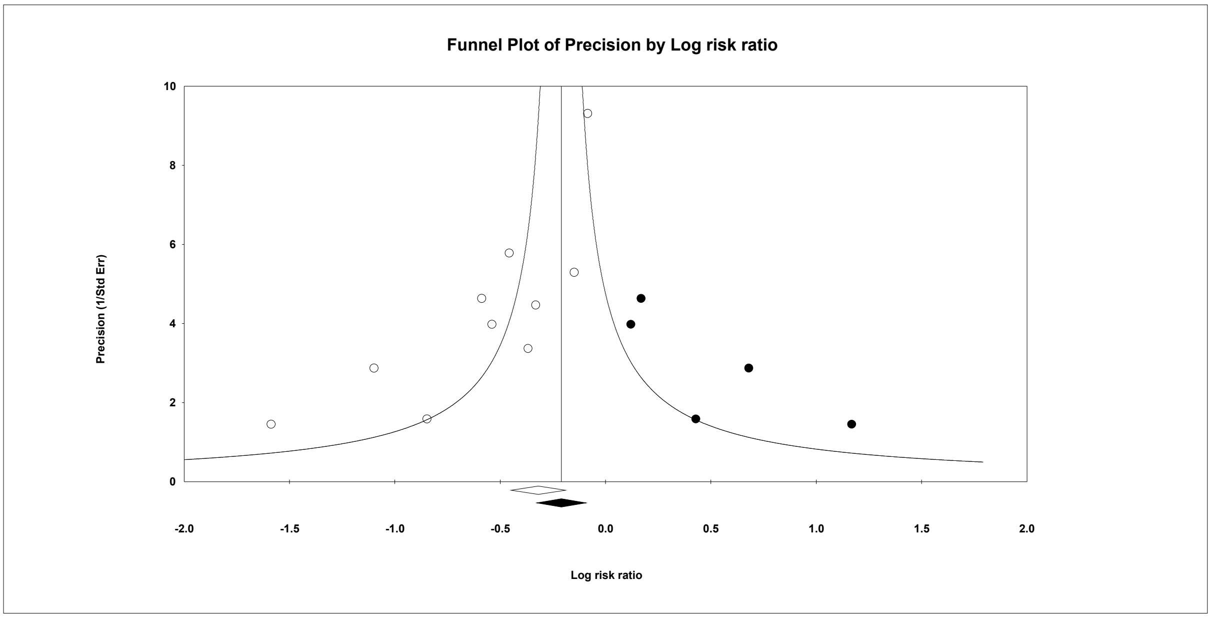

Supplementary Figure S77. Funnel plot for post-operative nausea and vomiting compared with control including studies comparing dexamethasone and tropisetron combined with dexamethasone and comparing metoclopramide and tropisetron combined with metoclopramide. The horizontal axis demonstrates the log of risk ratio, and the vertical axis shows the inverse of the standard error.

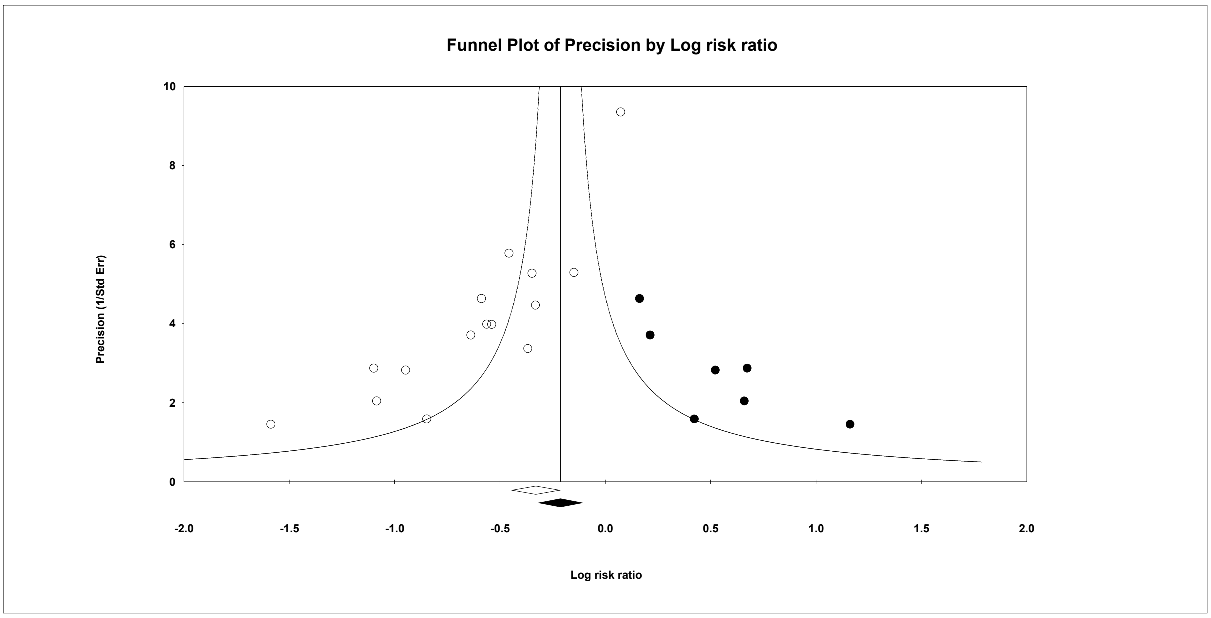

Supplementary Figure S78. Funnel plot for use of rescue anti-emetics compared with control . The horizontal axis demonstrates the log of risk ratio, and the vertical axis shows the inverse of the standard error.

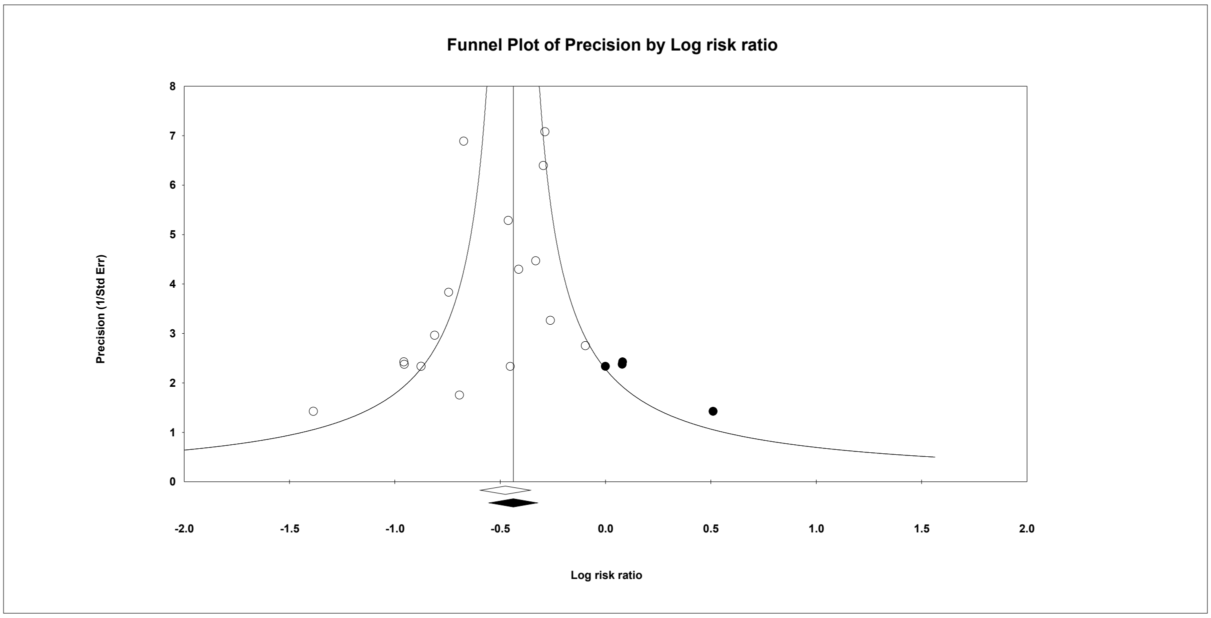

Supplementary Figure S79. Funnel plot for use of rescue anti-emetics compared with control including studies comparing metoclopramide and tropisetron combined with metoclopramide, dexamethasone and tropisetron combined with dexamethasone, droperidol and tropisetron combined with droperidol. The horizontal axis demonstrates the log of risk ratio, and the vertical axis shows the inverse of the standard error.

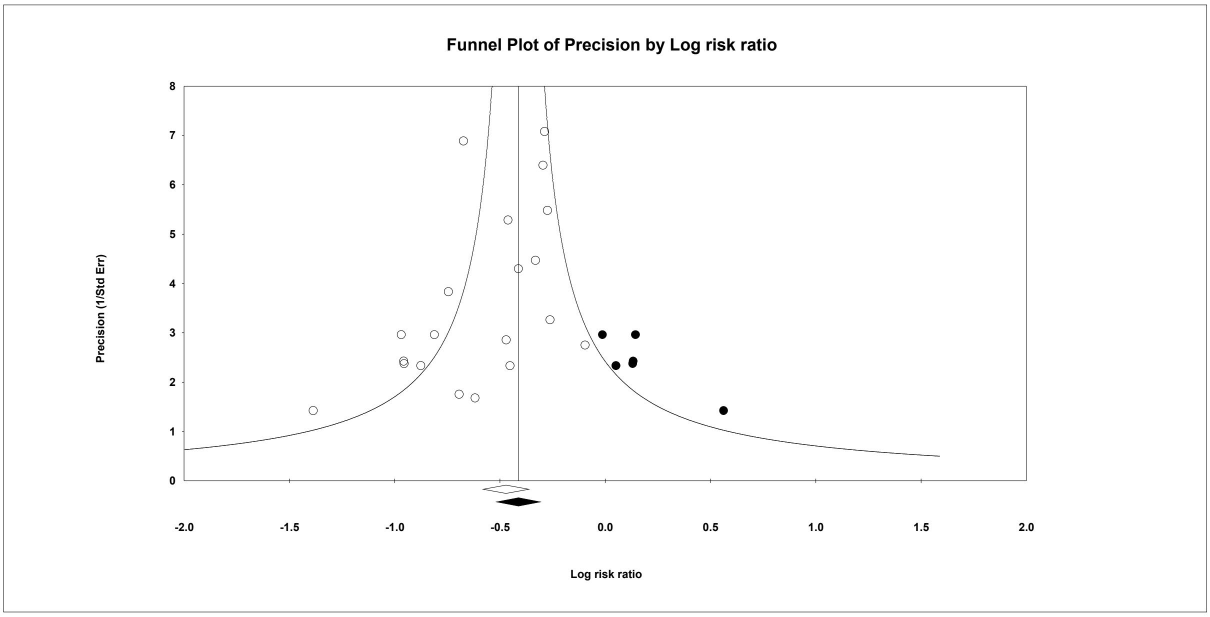

Supplement: Supplementary file 1 [file jpm-14-00797-s001.zip › jpm-3091008-supplementary.pdf]
